# In the Chalcogenoxide Elimination Panorama: Systematic Insight into a Key Reaction

**DOI:** 10.1021/acs.joc.2c01454

**Published:** 2022-08-11

**Authors:** Andrea Madabeni, Simone Zucchelli, Pablo A. Nogara, João B. T. Rocha, Laura Orian

**Affiliations:** † Dipartimento di Scienze Chimiche, Università degli Studi di Padova, Via Marzolo 1, 35131 Padova, Italy; ‡ Departamento de Bioquímica e Biologia Molecular, Universidade Federal de Santa Maria (UFSM), Santa Maria, 97105-900, RS, Brazil

## Abstract

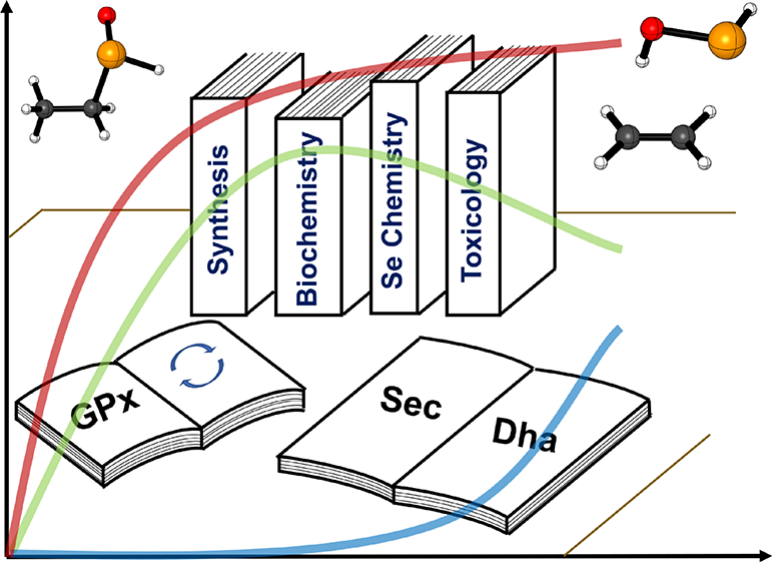

The selenoxide elimination is a well-known reaction in organochalcogen chemistry, with wide synthetic, biological, and toxicological implications. In this work, we apply benchmarked density functional theory (DFT) calculations to investigate different aspects of the title reaction in three (bio)chemically relevant models, spanning minimal systems of theoretical interests as well as biological or synthetic organochalcogenides. The activation strain analysis (ASA) methodology is employed along a suitable reaction coordinate to obtain insight into the role of the chalcogen and of the oxidation state, to pinpoint the factors that tune the elimination reactivity of the investigated systems. Lastly, we computationally validate the hypothesis that telluroxides eliminate more slowly than selenoxides because of a detrimental hydration process that leads to unreactive hydrates.

## 1 Introduction

**Scheme 1:**
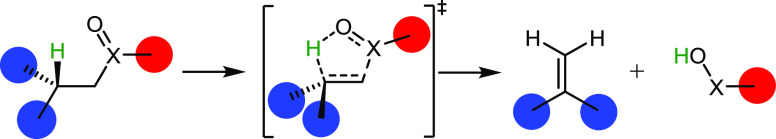
General Selenoxide (X = Se) or Chalcogenoxide (X = S, Se, Te) Elimination Reaction Circles are organic groups or peptide/protein chains. The transition state for the reaction is represented between squared parentheses.

The so-called selenoxide elimination is a convenient reaction to easily introduce a C=C bond in an organic scaffold.^1^ It requires a selenylating agent and a suitable oxidant to generate *in situ* the selenoxide moiety. Moreover, the actual C=C bond formation proceeds smoothly at room temperature or even below 0 °C.^1^ The reaction can be part of green synthetic protocols, based on selenylation–deselenylation catalytic cycles, in which hydrogen peroxide can be used as the oxidant.^2−4^ This elimination was serendipitously discovered by Jones et al. in 1970,^5^ and its scope was further analyzed by Sharpless *et al.* and Reich *et al.* in 1973.^6−9^ The mechanism of the reaction is well recognized to be an E_i_ elimination, in which the selenoxide moiety abstracts one proton in β, leading to a selenenic acid and to the desired C=C bond formation^5−7^ (Scheme 1). Within the oxidizing conditions in which the reaction occurs, the selenenic acid usually undergoes further reactivity and is thus undetected.

The same reaction for sulfoxides and telluroxides is known too^10,11^ but usually proceeds at higher temperatures. Indeed, while selenoxides are recognized to eliminate much faster than the analogous sulfoxides, telluroxides usually eliminate somewhat slower than analogous selenoxides,^12,13^ an aspect which was recognized already by Sharpless in 1975.^14^ To the best of our knowledge, this behavior was rationalized by formulating two hypotheses, i.e., (i) due to the longer X=O bond for Te than for Se, the β-proton cannot be properly abstracted because of geometric constrains; and (ii) the higher tendency of telluroxides to form hydrates transforms Te=O in Te–OH, thus preventing the elimination. However, a unique conclusion was never reached.^12^

The same reactions for the highly oxidized systems (i.e., sulfones, selenones, and tellurones) are much less investigated, even if all of these systems formally have a chalcogen=oxygen bond, which may promote elimination. In a combined experimental and theoretical study by Cubbage *et al*.,^15^ sulfones were demonstrated to eliminate via the E_i_ mechanism only at very high temperatures (above 400 °C). On the other hand, while selenones and tellurones might decompose above 100 °C, to the best of our knowledge, their decomposition mechanism was never investigated.^1,16^

Chalcogenoxide eliminations occur also in biological chemistry.^17,18^ In general, the chalcogenoxide elimination can alter the function of cysteine (Cys) and selenocysteine (Sec) containing proteins, such as albumin,^19^ glyceraldehyde-3-phosphate dehydrogenase,^20^ and peroxiredoxins,^18^ leading to potential toxic effects facilitating protein cross-linking, protein–protein aggregation, and protein aging due to the formation of dehydroalanine (DHA).^21^ Moreover, in 2010, Cho *et al.* proposed that in conditions of oxidative stress, the selenocysteine (Sec) of the selenoenzyme glutathione peroxidase (GPx) might undergo deselenylation via a selenoxide elimination reaction of an unknown intermediate (likely a seleninic acid), leading to the DHA residue. Orian *et al.*(22) and, more recently, Masuda *et a*l.^23^ proved that a bypass mechanism exists to prevent DHA formation in a fully functional enzyme and in a peptide mimic, based on the formation of a Se–N bond within the catalytic pocket. However, in the absence of a suitable partner for the formation of the Se–N bond, such as it happens by disruption of the protein architecture after tryptic digestion,^22^ DHA formation can still occur in highly oxidizing conditions. Moreover, while *in vivo* the protein architecture should protect selenocysteine deselenylation, in 2019, Reddy *et al.*(24) discovered that small-molecule inhibitors of the selenoenzyme thioredoxin reductase (TrxR) can bind to Sec leading to an oxidation–elimination mechanism, with consequent DHA formation. A similar mechanism for methylmercury toxified Sec and Cys was also computationally proposed and investigated by some of us.^25^ Thus, the interest in the sulfoxide/selenoxide elimination within thiol/selenoenzymes biochemistry remains alive.

Despite its role in organic and biological chemistry, the chalcogenoxide elimination reaction was investigated *in silico* by density functional theory (DFT) calculations only for very specific systems^26−31^ and without a properly benchmarked level of theory. With this regard, Mcdougall *et al.*(27) compared the results of the popular B3LYP density functional to highly correlated *ab initio* methods for the investigation of selenoxide elimination, but no other density functionals were tested, nor B3LYP performance was investigated for sulfoxides or telluroxides elimination reactions. Thus, in this work, we aim at filling some gaps in the understanding of the title reaction. Once assessed the most suitable DFT method(s) to computationally tackle the title reaction, also by evaluating the degree of error that comes with using a less accurate protocol, our scope is manifold: (1) To quantify and rationalize the effect of the chalcogen on the reaction and that of its oxidation state (OS) in bioinspired chalcogenoxide eliminations; (2) to provide an explanation to why telluroxides eliminate somewhat more slowly than selenoxides, a question that, to the best of our knowledge, has remained open in the last 40 years.

## 2 Results and Discussion

**Scheme 2:**
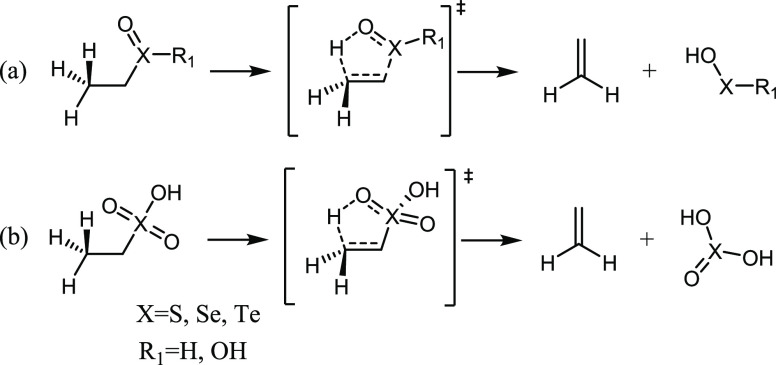
General Scheme of the Elimination Reactions in Organochalcogenoxides (Minimal Models)

With the aim of obtaining seminal insight for a rigorous analysis of the chalcogenoxide elimination and for a clear and quantitative understanding of the effect of the chalcogen and of the OS on this reaction, we chose to study first a minimal model, that is, the simplest compounds that can theoretically undergo the β-elimination process. (Scheme 2).

**Scheme 3:**
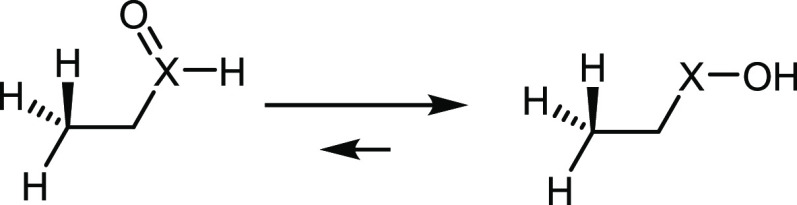
Tautomeric Equilibrium between a Chalcogenoxide (Left) and a Chalcogenenic Acid (Right) The equilibrium is so shifted to the right that only the chalcogenenic acid is present in biological conditions (e.g., in the catalytic cycle of GPx).^32^

For the elimination process to occur, one β-proton must be preserved in all the models. Thus, in the simplest compounds, the chalcogenoxide moiety bears an ethyl substituent on. Keeping in mind the biological problem of selenocysteine deselenylation,^17^ the R_1_ substituent (Schemes 1 and 2a) was chosen to be either H, in the lowest OS (0) or OH in the intermediate OS (+2). These two states correspond to the oxidation state of chalcogenenic and chalcogeninic acids, respectively. Since the real chalcogenenic acid does not have a chalcogen=oxygen bond, in order to investigate the whole range of oxidation states, the tautomer was used to obtain theoretical insight and trends. (Scheme 3).

Notably, this system is also the simplest model for a general chalcogenoxide elimination as exploited in synthetic organic chemistry, where R_1_ is usually an alkyl or aryl function. Lastly, the reaction represented in Scheme 2b proceeds from the highest oxidation state possible (+4) and is the model of a general chalcogenonic acid elimination. For the OSs 0 and +2, the reaction can proceed along two enantiomeric pathways due to the presence of the chalcogenoxide stereogenic center. Since the two pathways have the same activation energy, only one of them was investigated for all chalcogens and OSs.

Then, the elimination chemistry of the oxidized cysteine (Cys), Sec, and tellurocysteine (Tec) was investigated. In this case, for the OS 0 and +2, two diastereoisomeric compounds can undergo elimination because of the combination of the chalcogen (X = S, Se, Te) and the C_α_ stereogenic centers. Due to the biochemical importance of the substrates and for completeness, both pathways were investigated.

Lastly, we investigated a case of general interest in mechanistic organochalcogen chemistry, that is, understanding why selenoxides eliminate faster than sulfoxides, but telluroxides tend to be more inert toward the analogous elimination pathway.

### 2.1 Minimal Model Elimination Reactions

As described in the Computational Methods (See **4**), the reactants (R), transition states (TS), and products (P) of the title reaction were preliminarily optimized with OLYP and OPBE functionals, and accurate energetics have been obtained running single points with CCSD(T) and M06 functional, respectively. The choice of the M06//OPBE protocol is described in detail in the SI. Geometries and energies for the reaction depicted in Scheme 2 were computed for X = S, Se, and Te and for R_1_ = H, OH, in order to encompass all chalcogens and to span all the relevant oxidation states. Since in a previous structural benchmark on organochalcogenides, the OLYP functional provided geometries in excellent agreement with crystallographic data,^33^ OLYP-optimized coordinates were used to perform highly correlated single point energy calculations at the CCSD(T) level of theory as described in the Computational Methods. The results of these calculations are shown in Table 1.

**Table 1:** Activation (Δ*E*^‡^) and Reaction (Δ*E*_r_) Energies (kcal mol^–1^) for the β-Elimination Reaction of Chalcogenoxides (OS 0), Chalcogeninic Acids (OS +2), and Chalcogenonic Acids (OS +4)^a^

	Δ*E*^‡^			Δ*E*_r_		
OS	S	Se	Te	S	Se	Te
0	31.22(31.36)	23.70(23.66)	21.42(21.89)	11.23(16.17)	1.16(1.71)	–3.09(−2.03)
+2	38.80(37.91)	29.95(28.42)	26.91(25.92)	26.80(31.10)	14.34(13.67)	8.27(7.88)
+4	57.52(58.93)	37.86(34.93)	30.84(27.65)	22.75(28.86)	–9.83(−12.99)	–24.29(−28.45)

a Electronic energies computed at CCSD(T), (M06//OPBE).

**Figure 1:**
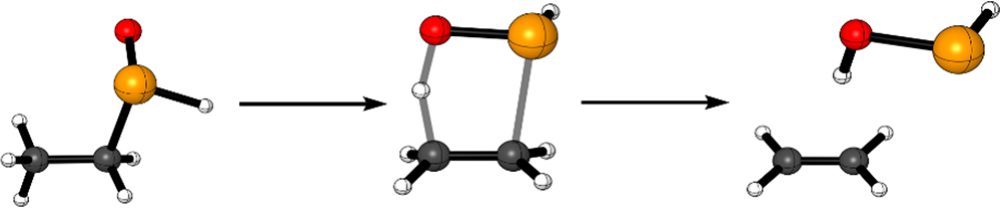
Representative reactant, transition state, and product of a chalcogenoxide elimination (X =Se, OS = 0); level of theory: ZORA-OPBE/TZ2P.

In all reactions, at the transition state, the oxygen atom of the chalcogenoxide moiety abstracts the β-proton, leading to the concerted breaking of the C–X bond and to the formation of a C=C bond. (Figure 1).

Focusing on the activation energies Δ*E*^‡^, the elimination becomes kinetically more favored going from S to Se and to Te, for all the OSs under investigation. The lowering in activation energy is significantly larger going from S to Se, while it becomes less dramatic when moving from Se to Te, even if it remains relevant. Particularly, in the OS 0, while the activation energy is lowered of ca. 8 kcal mol^–1^ going from sulfoxide to selenoxide, the telluroxide has an activation energy 2 kcal mol^–1^ lower than the latter.

Conversely, changing the OS from 0 to +2 and +4 leads to an increase in the activation energy independently of the nature of the chalcogen. The effect is remarkable for S, for which between the OS 0 and +4, there is an increase in activation energy of over 20 kcal mol^–1^, and far less remarkable for Te, with the telluronic acid having an activation energy ca. 9 kcal mol^–1^ higher than the telluroxide. Se displays an intermediate behavior, with an appreciable increase in activation energy upon increasing the OS of the chalcogen, but far from the dramatic behavior of the S analogs. In the OS +2, all systems display an activation energy 5–8 kcal/mol higher than that of the chalcogenoxide. These results agree with the experimental behavior of chalcogenoxides and chalcogenones, that is, while the elimination reactions for sulfoxides, selenoxides, and telluroxides are well-known, the analogous reactions for the highly oxidized systems proceed only at far higher temperatures or are not known to occur. Quite interestingly, these results suggest that the telluroxides should eliminate faster or as fast as selenoxides, in contrast to the experimental results. Thus, at least for these minimal models, we conclude that the geometrical features of the Te–O bond are not causing the experimentally observed kinetic inertia since for all our fully optimized systems, the activation energy for the telluroxide elimination is lower than for the Se corresponding case. Further details are reported in Section 3.4.

Similar trends were obtained for the reaction energies Δ*E*_r_, which in general become more negative when going from S to Se and to Te and more positive when increasing the OS. The only notable exception is an inversion in the expected trend when going from the OS +2 to the OS +4. In this case, while the activation energy increases, the reaction energy decreases, that is, the reaction becomes more favored. However, the high activation energy in the highest OS likely precludes the process anyway.

### 2.2 Cysteine, Selenocysteine, and Tellurocysteine Elimination Reactions

In order to expand the scope of our investigation to more realistic systems, we focused first on the problem of selenoproteins’ deselenylation. It must be stressed that while for Cys, all the OSs are available in a biological environment,^9^ because the oxidation to higher OSs (+2 and +4) has activation energies similar to the first oxidation^9,34^ (0), selenium is somewhat more resistant toward overoxidation. While the oxidation to seleninic acid (OS + 2) is possible, the oxidation to selenonic acid is three order of magnitude slower than the oxidation to the corresponding sulfonic acids.^9^ For the sake of completeness, however, the elimination behavior of Cys, Sec, and Tec was investigated spanning the same OSs of the minimal model, i.e., 0, +2, and +4. The results are shown in Table 2.

**Table 2:** Activation (Δ*E*^‡^) and Reaction (Δ*E*_r_) Energies (kcal mol^–1^) for the β-Elimination Reaction of Chalcogenoxides (OS 0), Chalcogeninic Acids (OS +2), and Chalcogenonic Acids (OS +4)^a^

	OS	configuration	Δ*E*^‡^	Δ*E*_r_
Cys	0	RR	22.34	6.65
		RS	28.34	11.59
	+2	RR	30.27	22.76
		RS	32.51	23.70
	+4	R	52.73	18.87
Sec	0	RR	18.54	–2.86
		RS	20.81	–1.35
	+2	RR	21.36	7.17
		RS	25.85	9.44
	+4	R	30.10	–23.04
Tec	0	RR	17.81	–4.66
		RS	18.28	–4.20
	+2	RR	19.13	3.72
		RS	26.83	8.51
	+4	R	24.82	–35.32

a Electronic energies computed at the M06//OPBE level of theory.

First, it can be noticed that the amino acid model follows essentially the same trends described for the minimal model. M06 predicts a slightly lower activation energy for the elimination of Tec in the OS +4 than in OS +2. However, from the benchmark (Figure S2), the M06 density functional somewhat underestimates the activation energy of systems in the highest OS. Indeed, the M06-2X density functional (Table S4), which is the best performer in the OS +4, predicts the expected increase in activation energy increasing the OS. Thus, also for the amino acid model, we confirm that the activation energy decreases increasing the size of the chalcogen (i.e., from S to Te), while it increases with increasing the oxidation state of the chalcogen (i.e., from 0 to +4). The two diastereoisomers present moderately different activation energies, with the RS diastereoisomer systematically displaying the highest one. This is likely due to the different stability of the diastereoisomeric reactants. In fact, the diastereoisomer displaying the highest elimination barrier is also the more stable between the two, e.g., in the OS 0, Cys (*R*,*S*) shows an activation energy ca. 6 kcal mol^–1^ higher than Cys (*R*,*R*), and the (*R*,*S*) diastereoisomer is ca. 5 kcal mol^–1^ more stable than the (*R*,*R*) one. Indeed, the energies of the two diastereoisomeric TSs are quite close (ca. 1 kcal mol^–1^ for Cys in the OS 0). While for the other OS and chalcogens, the effect can be less remarkable, the higher activation energy for the (*R*,*S*) remains partly due to reactant stabilization. The absolute energies of reactants and transition states are reported in Table S5.

Despite following identical trends as the minimal model, all the amino acids display significantly lower activation energies for the elimination process than the minimal model at the same level of theory (Tables 1 and 2). Cys displays the strongest decrease, while Sec and Tec show a more modest but still appreciable lowering. This effect is more prominent along the RR pathway, but it is still appreciable even for the higher-barrier RS elimination. This behavior is likely due to the increased acidity of the β-proton (i.e., the acid α-proton with respect to the carboxyl moiety), which can be more properly abstracted by the chalcogenoxide moiety. Even so, it can be seen that while the chemical environment (i.e., RS/RR configuration, β-proton acidity, etc.) can tune the reaction, the overall behavior of the process is rooted in the nature of the chalcogen, with sulfur displaying the highest and tellurium displaying the lowest activation energy, respectively. Importantly, also in this system, no selenium–tellurium inversion in the height of the barrier is revealed. Thus, also within a more amino acid systems, telluroxides should eliminate intrinsically faster than (or as fast as) selenoxides and lead to more stable products as displayed by the reaction energies. The only exception is for the OS +2, RS pathway, for which a slightly higher activation energy for Te than for Se is predicted. The difference between the two activation energies is however less than 1 kcal mol^–1^ (M06//OPBE) and thus is considered negligible. Overall, the amino acid systems and the minimal models behave alike.

### 2.3 Analysis of the Trends

**Figure 2:**
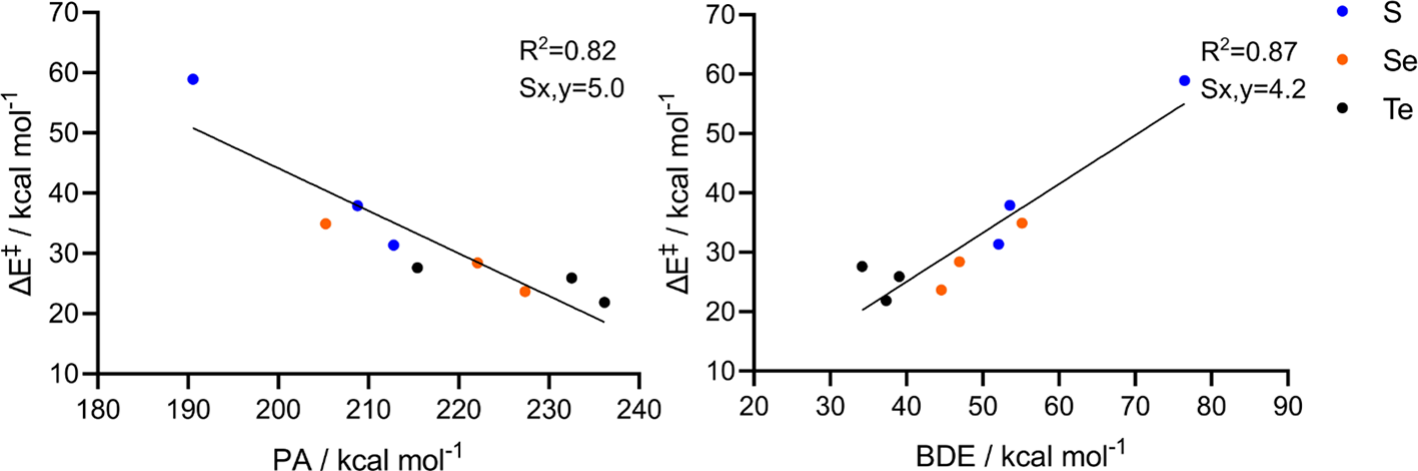
Linear correlation between activation energies and PA/BDE for the minimal model reactions (OS = 0, +2, +4). Statistical parameters (*R*^2^ and standard deviation, S*y*,*x*) are reported near the linear fit. Level of theory: M06//OPBE.

To the best of our knowledge, two main factors might concur to explain the increased reactivity of selenoxides over sulfoxides, i.e., the increased basicity of the selenoxide oxygen and the lower strength of the Se–C bond, when compared to the S–C bond, which help in the proton abstraction and in the X–C bond breaking occurring along the reaction, respectively.^1,9^ To quantify how the activation energy of the title reactions is related to the basicity of the chalcogenoxide and to the X–C bond strength, the Δ*E*^‡^ computed for the minimal models were plotted against the electronic proton affinity (PA) of the substrate (i.e., the capacity of the chalcogenoxide to abstract the β-proton) and against the electronic bond dissociation energy (BDE) of the C–X (X = S, Se, Te) bond in different substrates (i.e., the ease by which the carbon–chalcogen bond breaks). It is interesting to verify if these simple explanations can be extended also to telluroxides and to the whole plethora of OSs under investigation, and not just to the S and Se chalcogenoxides more commonly discussed in the experimental literature. The results are shown in Figure 2. For clarity, the systems are labeled by the chalcogen and oxidation states; thus, the sulfoxide is labeled S0, the sulfinic acid S2, and so on.

Overall, both the basicity of the chalcogenoxide, which extracts the proton along the reaction, and the strength of the C–X bond, which undergoes bond-breaking along the reaction, nicely correlate with the elimination activation energy. Particularly, considering the S and Se subgroups, the PA decreases and the BDE increases along the series OS 0, +2, and +4, in line with the increase in activation energy. Conversely, for all OSs, the PA increases and the BDE decreases when going from S to Se, in agreement with the lower activation energy required by all selenoxides to undergo elimination. For Te, the PA still decreases with increasing OSs. However, for Te, the BDE does not display a clear trend when plotted against the activation energy.

**Figure 3:**
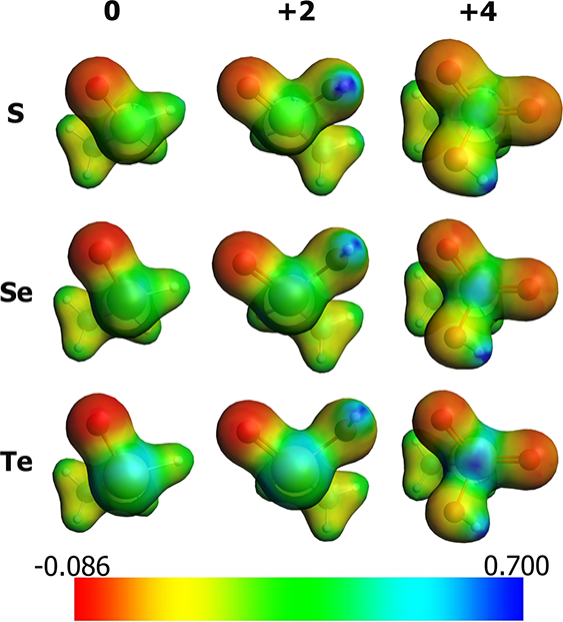
Molecular electrostatic potential (MEP) in a.u. for all the minimal model reactants. Columns are different OSs (0, +2, +4) while rows are different chalcogens (S, Se, Te). Areas in which the potential is more negative are of greater red intensity. Isodensity value: 0.04. Level of theory: M06 // OPBE. Hirshfeld partial charges on the oxygen atom can be found in the SI (Table S8).

Indeed, the PA trends correlate fairly well with the charge density analysis of the chalcogenoxide bond, that is, the X=O acquires a more charge separated character as the size of the chalcogen increases, leading to a more prominent negatively charged oxygen atom (i.e., more basic) than in their lighter analogs. Increasing the OS of the system leads to a more positively charged density on the chalcogen. Conversely, the charge density on the oxygen becomes less negative making the chalcogenoxide intrinsically less basic in high OSs despite the charge separated character of the bond. (Figure 3).

To provide a quantitative discussion on the effect of the chalcogen and OSs on the BDE, we performed ASA (see Computational Methods) on the BDEs previously shown (Table 3), using as fragments the two radical (unrestricted doublets) products of the bond dissociation event. This can help to rationalize the poor correlation between BDE and activation energies of telluroxide elimination, which is also affected by the softness of the fragments, while the bond strength should be more clearly represented by the actual Δ*E*_int_ of the bond. Indeed, increasing the size of the chalcogen along the series S, Se, and Te, the interaction energy of the C–X bond is weakened in agreement with the decrease in BDE. The situation is somewhat different for Te0, for which the BDE is lowered by an increase in Δ*E*_strain_. However, also in this case, with respect to Se0, Te0 has a (slightly) lower Δ*E*_int_, suggesting that telluroxides have an intrinsically weaker X–C bond than selenoxides and sulfoxides. Conversely, increasing the OS along the series S0, S2, and S4 and Se0, Se2, and Se4, Δ*E*_int_ becomes more and more stabilizing, and the BDE becomes higher. The situation is, also in this case, somewhat different for Te, for which the BDE displays an alternation effect due to the interplay between Δ*E*_strain_ and Δ*E*_int_. However, the Te–C bond in high OS remains intrinsically stronger as highlighted by a more negative Δ*E*_int_. Thus, while the BDE for the series Te0, Te2, and Te4 poorly correlates with the activation energy of the elimination process, the increase (in absolute values) in Δ*E*_int_ of the Te–C bond along the series agrees with the increasing activation energy of the process, as it does for the other two chalcogens.

**Table 3:** Activation Strain Analysis (ASA) and Energy Decomposition Analysis (EDA) of the X–C BDE (kcal mol^–1^)^a^

	BDE	BFE	Δ*E*_strain_	Δ*E*_int_
S0	52.06	–52.06	6.62	–58.68
Se0	44.57	–44.57	6.79	–51.36
Te0	37.32	–37.32	13.59	–50.91
S2	53.54	–53.54	7.90	–61.44
Se2	46.94	–46.94	8.59	–55.53
Te2	39.03	–39.03	15.70	–54.73
S4	76.45	–76.45	7.42	–83.87
Se4	55.13	–55.13	9.30	–64.43
Te4	34.16	–34.16	22.69	–56.85

a Both fragments are unrestricted doublets. Level of theory: M06//OPBE.

These results suggest indeed that both the chalcogenoxide basicity and the X–C bond strength (quantified as the BDE or, better, by the Δ*E*_int_) are phenomenologically valid explanations for the differences in reactivities of different chalcogens and oxidation states in chalcogenoxide elimination reactions and not just for S and Se in their lowest OS. However, no clear picture emerges about what factor actually controls the reactivity, if any, since the X=O basicity and the X–C bond strength appear to be somewhat intertwined.

**Figure 4:**
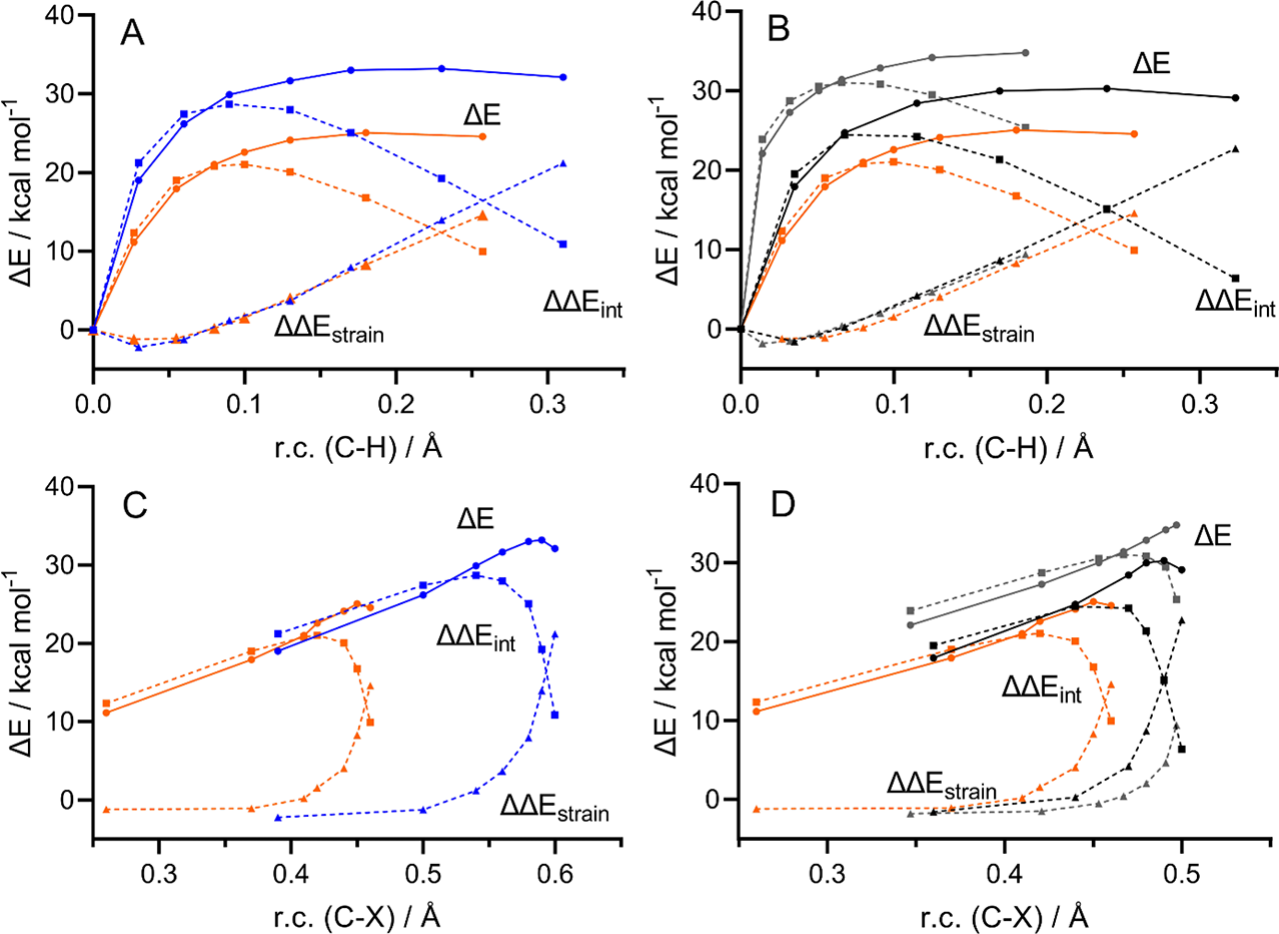
ASA along the reaction coordinate (r.c., C–H bond stretching (A, B) and C–X bond stretching (C, D)) for (A,C) the effect of the chalcogen, S0 (blue) vs Se0 (orange), and (B, D) the effect of the OS, Se0 (orange) vs Se2 (black) and Se4 (gray). Level of theory: M06//OPBE. The final point is the TS as identified along the intrinsic reaction coordinate at the OPBE level. The second-last point is at a slightly higher energy for S0, Se0, and Se2 after M06//OPBE single point. The reference point (r.c. 0.0) is the final point of the IRC profile. Along the C–X r.c., the reference point (0, 0) is left out of the plot for clarity, and the focus is on the region in the surrounding of the TS. Solid lines are IRC energies, while dashed lines are strain (triangles dots) and interaction (squared dots) energies.

Thus, to better rationalize the trends in activation energy, a few model cases were analyzed within the framework of the activation strain model (see Computational Methods). Particularly, this approach was used to compare the reactivity of S0 to Se0 and of Se0 to Se2 and Se4, thus obtaining insight in the role of the chalcogen and of the OS, respectively. The system was fragmented in the ethyl radical and in the chalcogenoxide moiety, both considered as unrestricted radical fragments (Figure 4).

The IRC profile was projected on two critical reaction coordinates (r.c.), i.e., the C–H and the X–C bond breaking. For well-behaved reactions, ASA along different r.c.s should provide similar or compatible results. However, chalcogenoxide eliminations appeared to have a somewhat pathological behavior likely due to different reasons: they are intramolecular reactions, thus making ASA *per se* more challenging; C–H and X–C bond breaking do not proceed simultaneously; the protophile and the leaving group of the elimination are the same function, thus their role can be envisioned to be somewhat entangled. Indeed, while between the two r.c.s, there are some similarities, the two analyses provide interestingly different results.

Comparing the S0 to the Se0 curves along the C–H r.c., the whole reaction profile of the selenoxide is lower in energy with respect to the sulfoxide. Since the two ΔΔ*E*_strain_ profiles are essentially superimposed, the lower reaction profile of Se0 is due to the less destabilizing ΔΔ*E*_int_, that is to the less prominent decrease in Δ*E*_int_ when going from the reactant to the TS. In the end, the shape of the ΔΔ*E*_int_ curve determines the reactivity. This term is likely due to the contribution of two concomitant main phenomena, the breaking of the X–C bond and the formation of the O–H bond, with the first one being predominant in the early stages of the reaction, when the ΔΔ*E*_int_ undergoes an abrupt increase despite a modest elongation of the C–H bond, and the second one being predominant later on, providing an extra stabilization, which lowers the interaction leading to the observed single-maximum profile. Indeed, as previously discussed, both increased basicity of selenoxide and the lower strength of the Se–C bond, when compared to the S–C bond, have been used in the literature to explain the increased reactivity of selenoxides with respect to sulfoxides.^1,9^ Both these aspects are captured by the shape of the interaction profile and appear to contribute to the less destabilizing ΔΔ*E*_int_ and, in the end, to a lower activation energy. A similar discussion can be made when comparing Se0 to Se2, for which a single-maximum profile is observed for ΔΔ*E*_int_. In this case, not only Se0 has a less destabilizing ΔΔ*E*_int_ but also a slightly lower ΔΔ*E*_strain_. However, the interaction energy remains the main difference at the origin of the different reactivity.

Along the X–C r.c., the ΔΔ*E*_int_ naturally has the same single-maximum shape. However, some details reveal a different picture. Particularly, the two ΔΔ*E*_strain_ profiles are not anymore superimposed, neither for the effect of the chalcogen (bottom, left) nor for the effect of the OS (bottom, right), and the reactions with the highest barrier (i.e., S0, and Se2) display a later increase in the strain energy compared to Se0. Indeed, this is due to the fact that along this r.c., the two ΔΔ*E*_int_ are initially almost superimposed (with S0 and Se2 displaying only a slightly more destabilizing interaction compared to Se0) and only in proximity of the TS, where the ΔΔ*E*_int_ starts to decrease because of the O–H interaction, the two curves begin to show strong differences. Indeed, Se0 ΔΔ*E*_int_ starts to decrease earlier compared to S0 (left) and compared to Se2 (right). This aspect, which is not clearly captured along the C–H r.c., suggests that while the softer Se0–C bond might provide some advance over the stronger S0–C and Se2–C bond, the increased reactivity of selenoxides over sulfoxides and of the lowest OS over the intermediate OS is mostly due to the point along the r.c. at which the interaction between the protophile and the β-hydrogen becomes relevant.

A different argument can be made when the highest OS (Se4) is analyzed and compared to the two lower ones. Indeed, while along the C–H r.c., the same picture can be seen; with a single-maximum profile for the interaction energy and a smooth increase in ΔΔ*E*_int_ going from Se0 to Se2 and to Se4, in this case, Se4 reaches the TS earlier than Se2, despite having an even higher ΔΔ*E*_int_ profile. Also in this case, the behavior of the reaction becomes clearer when it is observed along the X–C reaction coordinate: in this case, while the Se4 strain profile is the last one to undergo an increase in the proximity of the TS, its ΔΔ*E*_int_ is significantly higher compared to Se0 and Se2, which, as previously discussed, display closer ΔΔ*E*_int_ at similar r.c. values until the TS surrounding is reached. Thus, while the TS is still reached after the ΔΔ*E*_int_ decreases because of the onset of the O–H interaction, in this case, it is the overall highest ΔΔ*E*_int_ profile that leads to the higher activation energy. Thus, for the highest OS, the energy required for X–C bond-breaking becomes determinant over the protophilicity of the chalcogenoxide itself.

Overall, this combined PA/BDE correlation and ASA investigation suggest that the simple explanations commonly found in the literature for S0 and Se0 can also be phenomenologically extended to the higher OSs (+2 and +4). It is clear from the ΔΔ*E*_int_ profile that the X–C bond breaking and O–H bond formation effects are intertwined. However, despite both the basicity of the chalcogenoxide (quantified as the PA) and the strength (quantified as the BDE or, even better, by the Δ*E*_int_ of the X–C bond) correlating well with the whole plethora of reactions, ASA uncovered that for selenoxides, there is an earlier onset of the protophile−β-proton interaction with respect to sulfoxides (at the same X–C bond breaking r.c.), which is mainly responsible for the lower activation energy of the selenoxide over the sulfoxide elimination. A similar behavior also characterizes the OS 0 with respect to the OS +2, while for the OS +4, a stronger X–C bond significantly contributes to the heightening of the barrier.

### 2.4 Elimination of OS 0 Phenyl-Alkyl Chalcogenoxides

**Scheme 4:**
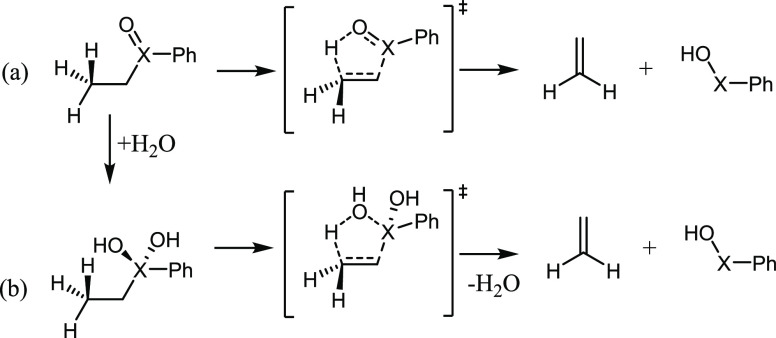
Direct Chalcogenoxide Elimination Mechanism of PhXEt (a) and Hydration Followed by a Dehydration Elimination Mechanism (b) as Investigated in This Study

Lastly, we applied the methodological and theoretical knowledge up to now obtained to investigate the elimination mechanism of phenyl-alkyl-chalcogenoxides to shed some light on why many telluroxides appear to eliminate somewhat more slowly than analogous selenoxides, in sharp contrast with the results of our calculations so far reported. Phenyl-alkyl species have been chosen because they are employed as redox catalysts^28,35^ and in organic synthesis as β-eliminating systems.^6,36^ Phenyl-ethyl-sulfoxide, selenoxide, and telluroxide (PhXEt) have been selected as model compounds since they are the smallest possible systems of this class that can theoretically undergo elimination. The elimination mechanism follows the same details previously explained, with a concerted transition state at which proton abstraction occurs along with the X–C bond breaking (Scheme 4a). The results (Δ*E*_elm_^‡^) are shown in Table 4.

**Table 4:** Activation Energies (kcal mol^–1^) Relative to the Direct Elimination Mechanism of PhXEt (Δ*E*_elm_^‡^), to the Elimination Mechanism of Their Hydrates (ΔE_hyd, elm_^‡^), and Reaction Energies^a^ for the Hydrates Formation (Δ*E*_*r*_^hyd^)

	Δ*E*_elm_^‡^	Δ*E*_*r*_^hyd^	Δ*E*_hyd, elm_^‡^
PhSEt	31.99	21.96	33.49
PhSeEt	25.22	–0.11	33.18
PhTeEt	23.52	–18.07	39.44

a Electronic energies computed at the M06//OPBE level of theory. Gibbs free energies follow the same qualitative trends and are available in the SI (Table S7).

As expected, also in this case, the sulfoxide displays the highest activation energy, with the selenoxide and telluroxide showing much lower and similar activation energies, i.e., also for phenyl-ethyl species, no intrinsic inertia toward the elimination seems to characterize telluroxides.

**Figure 5:**
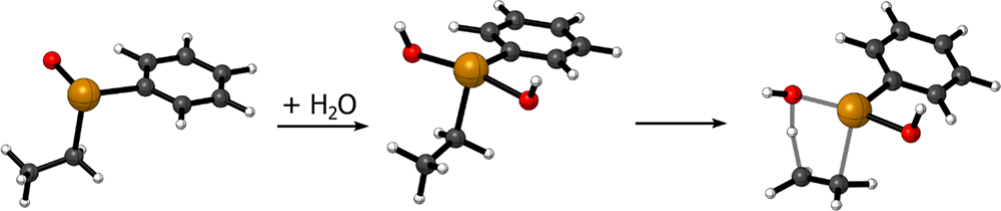
Representative phenyl-ethyl-chalcogenoxide, hydrate, and transition state for the hydrate elimination (X = Te).

In 1983, in their study on telluroxide elimination, Uemura *et al.* realized that all their “telluroxides” were in fact characterized as the corresponding hydrates^12^ and hypothesized that the hydration might lead to slowing down the reaction of telluroxides. Thus, we computed the reaction energy for the addition of one water molecule to PhXEt, and we tried to verify if the corresponding hydrates might undergo an elimination process even in the absence of a X = O bond (Scheme 4b and Figure 5).

Interestingly, in agreement with the studies of Uemura *et al*., PhTeEt undergoes a more favorable hydration than both sulfoxides and selenoxides.^12^ This is expected because descending along a group in the periodic table, the elements can host more favorably hypervalent interactions,^9^ and telluroxides have a more positive electrostatic potential around the chalcogen than selenoxides and sulfoxides (Figure 3). Indeed, the stability of the hydrates smoothly increases along the series S, Se, and Te. Unexpectedly, given the lack of X=O bonds, all the hydrates can still undergo an elimination mechanism, with a transition state similar to the one of the conventional chalcogenoxide elimination (Scheme 4b and Figure 5) in which one of the −OH functions of the hydrate promotes the abstraction of the β-proton. However, all these TSs are located on the PES at higher energies with respect to the correspondent chalcogenoxide elimination TSs, and all the activation energies are higher than the correspondent sulfoxide elimination, making the reaction much less favorable (Table 4). Since the hydration process is much more favorable for Te than for the lighter chalcogens, we conclude that it is the primarily responsible factor behind the relatively slow telluroxide elimination in water-rich environments.

## 3 Conclusions

In this work, we have investigated *in silico*, with highly correlated *ab initio* methods and properly benchmarked DFT protocols, various aspects of the so-called chalcogenoxide eliminations. The results of this study are manifold and can be summarized as follows:

1. DFT approaches and CCSD(T) provide the same qualitative conclusions about the behavior of the title reactions, that is, the activation energy of the process decreases increasing the size of the chalcogen (along the series S, Se, and Te), with a sharp decrease from S to Se and a moderate decrease from Se to Te, and smoothly increases increasing the OS of the chalcogen (along the series 0, +2, and +4). This behavior is shared among systems of different complexity and is thus rooted in the property of the chalcogen itself.
2. For Sec, the OS 0 gives the most favorable (from the kinetic point of view) elimination. However, since in biological environments the OS 0 is represented by a selenenic acid and not by a chalcogenoxide (the tautomeric equilibrium is shifted to the chalcogenenic acid side), overoxidation to seleninic acid is confirmed to be necessary for the elimination process to occur. Overoxidation to selenonic acid, besides being slow, would lead to an even higher activation energy for the elimination, further preventing the elimination from occurring. Conversely, Cys is known to be easily oxidized, even in biological media, to high OS (+2, +4) where the elimination is kinetically disfavored. However, oxidized disulfides might still be involved in a rich elimination chemistry as shown in previous studies.^18,20^
3. Both the chalcogenoxide basicity and the X–C bond strength correlate well with the computed activation energy of all chalcogens and all OSs. Activation strain analysis showed how these two effects are intertwined at the same time providing insight into how selenoxides react faster than sulfoxides because of an anticipated interaction between the protophile and the β-proton.
4. From our analyses, telluroxides are the best eliminating systems even in the higher OSs. Thus, we conclude that the known inertia of organotellurides toward elimination is not due to intrinsic geometric factors but to their more favorable hydration process, which disrupts the Te=O bond fundamental for an effective β-proton abstraction.

We believe that this investigation provides systematic insight into this fundamental reaction, encompassing simple models of theoretical interest as well as biological or synthetic compounds. In addition, the benchmarked level of theory can be used to quantitatively investigate the inhibition of selenoproteins by small molecules and the elimination chemistry of oxidized dichalcogenides,^18,19^ thus paving the route for a deeper mechanistic understanding of post-translational modifications in biological and toxicological chemistry, based on this fundamental organochalcogen reaction. In these cases, residues close to the Cys or Sec might affect (promoting or disfavoring) the reaction, and their role should be carefully assessed in future investigations.

## 4 Computational Methods

All DFT calculations were done with the Amsterdam Density Functional (ADF) software.^37,38^ The computational protocol and its benchmarking are thoroughly described in the Supporting Information (SI, see additional computational details and extended benchmark discussion and Tables S1–s3). In this section, only the protocols employed along the main text will be described. For all DFT mechanistic calculations, geometries were optimized employing the OPBE functional^39−41^ with the Slater type TZ2P basis set, combined with a small frozen core approximation to treat the core electrons. This basis set is of triple-ζ quality and is augmented with two sets of polarization functions on each atom. Scalar relativistic effects were included in all calculations within the zeroth-order regular approximation^42^ (ZORA) as implemented in ADF. Energies have been refined as single points employing the meta-hybrid M06 density functional,^43^ combined with an all-electron TZ2P basis set (TZ2P-ae). Thus, DFT energetics discussed along the manuscript are at the ZORA-M06/TZ2P-ae//ZORA-OPBE/TZ2P level of theory, which will be shortly denoted as M06//OPBE. The nature of all stationary points was verified by frequency analysis on ZORA-OPBE/TZ2P optimized geometries: all minima display only positive frequencies, while transition states display only one imaginary frequency associated with the motion along the reaction coordinate from reactants to products.

Highly correlated CCSD(T) energies were calculated by means of the DLPNO-CCSD(T) method,^44^ as implemented in the Orca 4.2.1 package.^45,46^ All-electron relativistic contracted basis set aug-cc-pVTZ-DK with Douglas–Kroll–Hess (DKH) scalar relativistic Hamiltonian was used for all atoms.^47,48^ Geometries optimized with the OLYP functional^41,49^ were used as a starting point for the calculation of highly-correlated energies. This level of theory is denoted as DLPNO-CCSD(T)/aug-cc-pVTZ-DK//ZORA-OLYP/TZ2P. Along the manuscript, it will be simply referred to as CCSD(T). Both the OPBE and OLYP functionals proved to well reproduce organochalcogenides geometries in a previous benchmark study.^33^ For the amino acid model, the conformation was chosen from a previously published paper of some of us^25^ based on the most stable conformer for Cys as identified by Wilke *et al*.^50^

To obtain quantitative insight on bond energies, the activation strain analysis (ASA)^51,52^ was performed. This method was useful in gaining insight into several substitution and elimination reactions.^53−57^ Within the framework of ASA, any ΔE value can be decomposed into strain (Δ*E*_strain_) and interaction (Δ*E*_int_) contributions relative to two chemically meaningful fragments. The terms can be evaluated at any point along a reaction coordinate (ζ), on a reaction profile computed by means of an intrinsic reaction coordinate calculation^58^ (IRC):
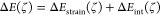
1where Δ*E*_strain_ represents the energy required to distort the fragments as they appear in the geometry under investigation, and Δ*E*_int_ is the stabilizing interaction between the two fragments.

While ASA was designed to investigate bimolecular reactions, it was extended to tackle also intramolecular reactions.^57,59^ In this case, both the strain and the interaction terms can be expressed as differences with respect to an initial reference, usually the reactant of the reaction:
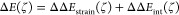
2

Conversely, when ASA is performed breaking a covalent bond, the bond dissociation energy (BDE) for such bond-breaking is related to the ASA terms by the equation:
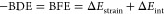
3that is, the sum of strain and interaction is equal to the BDE taken with negative sign, i.e., to the bond formation energy (BFE).

Molecular structures were illustrated using CYLview.^60^

## Supporting Information Available

Cartesian coordinates, electronic energies, and imaginary frequencies of the investigated compounds; additional computational details; extended benchmark results, activation and reaction energies computed with all the tested density functionals (PDF)
