# Supplementary material for: In the Chalcogenoxide Elimination Panorama: Systematic Insight into a Key Reaction
Source: J Org Chem. 2022 Aug 11;87(17):11766–75. doi: 10.1021/acs.joc.2c01454 (PMC9442651; doi:10.1021/acs.joc.2c01454)
Supplement: Supplementary file 1 — jo2c01454_si_001.pdf [file jo2c01454_si_001.pdf]

SUPPORTING INFORMATION

In the Chalcogenoxide Elimination Panorama: Systematic Insight into a Key Reaction

Andrea Madabeni,[a] Simone Zucchelli,[a] Pablo A. Nogara,[b] João B. T. Rocha,[b] Laura Orian [a,*]

[a] Dipartimento di Scienze Chimiche, Università degli Studi di Padova, Via Marzolo 1, 35131 Padova, Italy.

[b] Departamento de Bioquímica e Biologia Molecular, Universidade Federal de Santa Maria (UFSM), Santa Maria, 97105-900, RS, Brazil.

*Author to whom correspondence should be addressed, laura.orian@unipd.it

Tables of Contents

Additional Computational Details..........S3

Extended Benchmark Discussion..........S4

Table S1. Activation and reaction electronic energies (kcal mol^{-1}) for the β-elimination reaction of chalcogenoxides (OS 0), chalcogeninic acids (OS +2) and chalcogenonic acids (OS +4). Level of theory: ZORA-OPBE/TZP-ae; TZ2P(-ae); QZ4P-ae. S7

Table S2. Activation and reaction electronic energies (kcal mol^{-1}) for the for the β-elimination reaction of chalcogenoxides (minimal model) in OS 0, +2, +4. Level of theory: ZORA-xc/TZ2P(-ae)..........S8

Table S3. Activation and reaction electronic energies (kcal mol^{-1}) for the β-elimination reaction of chalcogenoxides (OS 0), chalcogeninic acids (OS +2) and chalcogenonic acids (OS +4). Level of theory: xc // OPBE. S8

Table S4. Activation ($\Delta E^{\ddagger}$) and reaction (ΔE_r) energies (kcal mol^{-1}) for the β-elimination reaction of chalcogenoxides (OS 0), chalcogeninic acids (OS +2) and chalcogenonic acids (OS +4).[a]..........S11

Table S5. Electronic energies (kcal mol^{-1}) for the cysteine, selenocysteine and tellurocysteine chalcogenoxide elimination in OS 0, +2, +4. Level of theory: M06 // OPBE. S11

Table S6: Activation and reaction Gibbs free energies (kcal mol^{-1}) for the minimal model reactions. Level of theory: M06 // OPBE...........S12

Table S7: Activation Gibbs free energies (kcal mol^{-1}) relative to the direct elimination mechanism of PhXEt ($\Delta \mathrm{Gelm}\ \ddagger$), to the elimination mechanism of their hydrates ($\Delta \mathrm{Ghyd, elm}\ \ddagger$), and reaction energies for the hydrates formation ($\Delta \mathrm{Grhyd}$). Level of theory: M06 // OPBE. S12

Table S8: Hirshfeld partial charges (a.u.) on the X = O oxygen atom of chalcogenoxides in different oxidation states. S12

Table S9: Cartesian coordinates (Å), energies (a.u.) and imaginary frequencies (cm^{-1}) of the optimized structures (minimal model). Level of theory: ZORA-M06-2X/TZ2P-ae. S13

Table S10: Cartesian coordinates (Å), energies (a.u.) and imaginary frequencies (cm^{-1}) of the optimized structures of the minimal model. Level of theory: ZORA-OPBE/TZ2P. S19

Table S11: Cartesian coordinates (Å), energies (a.u.) and imaginary frequencies (cm^{-1}) of the optimized structures of the minimal model. Level of theory: ZORA-OLYP/TZ2P..........S25

Table S12: Cartesian coordinates (Å), energies (a.u.) and imaginary frequencies (cm^{-1}) of the optimized structures of the minimal model. Level of theory: ZORA-B3LYP/TZ2P-ae. S31

Table S13: Cartesian coordinates (Å), energies (a.u.) and imaginary frequencies (cm^{-1}) of the optimized structures of the minimal model. Level of theory: ZORA-BLYP-D3(BJ)/TZ2P...........S37

Table 14: Cartesian coordinates (Å), energies (a.u.) and imaginary frequencies (cm^{-1}) of the optimized structures of the amino acid model. Level of theory: ZORA-OPBE/TZ2P...........S43

Table S15: Cartesian coordinates (Å), energies (a.u.) and imaginary frequencies (cm^{-1}) of the optimized structures for the phenyl alkyl model. Level of theory: ZORA-OPBE/TZ2P...........S53

Additional References S56

Additional Computational Details

For the benchmark, a total of five functionals (xc), i.e., two GGAs, OLYP[1–3] and OPBE;[4] one dispersion-corrected GGA, BLYP-D3(BJ);[1–3,5–8] one hybrid, B3LYP[9,10] and one meta-hybrid M06-2X[11,12], were preliminarily tested for the geometry optimization and energy calculations. The Slater type TZ2P basis set was used for all calculations. This basis set is of triple-ζ quality and augmented with two sets of polarization functions on each atom. For the three GGA, the small frozen core approximation was used, while for the hybrid and the metahybrid, all-electron calculations were performed since frozen core approximation is not implemented in ADF for these functionals. The role of the basis set (TZP, TZ2P and QZ4P) and of frozen core approximation (no frozen core and small core approximation) was tested for the OPBE functional, by reoptimizing all the investigated geometries and computing activation and reaction energies (Table S1) Scalar relativistic effects were included in all calculations within the zeroth-order regular approximation[13] (ZORA) as implemented in ADF. This level of theory is denoted as ZORA-xc/TZ2P(-ae). Starting from the OPBE optimized geometries (see main text), single point energies have been computed with eighteen different density functionals, i.e. ten GGAs (one dispersion-corrected GGA), two meta-GGAs, three hybrids and three meta-hybrids. In detail, BLYP,[2] BP86,[5,14] HTBS,[15] PBE,[16] mPW,[17] PW91,[18] revPBE,[19] RPBE,[20] mPBE[21] were considered. In addition, the dispersion-corrected version of BP86 functional, BP86-D3(BJ), was also tested. TPSS[22,23] and SCAN[24] functionals were tested for the meta-GGAs category; PBE0,[25] OPBE0[4] and mPW1PW[17] were tested for the hybrid category (the popular B3LYP was preliminarily tested in the main text); M06,[11] M06-2X[11] and TPSSh[22] were tested for the meta-hybrid category. Frozen core (fc) approximation was not used, to allow for a rigorous comparison, since for hybrids and meta-hybrids fc is not available. All calculations are all-electron except when explicitly specified. Following this initial investigation, eighteen functionals were tested (M06-2X was included as the best performing preliminary functional, while the other four were excluded given their relatively poor performance) by running single-point energy calculations on ZORA-OPBE/TZ2P optimized geometries. All calculations were done without frozen core approximation to allow a rigorous comparison. In total, ten GGAs (one dispersion-corrected GGA), two meta-GGAs, three hybrids and three meta-hybrids were tested. The level of theory of these calculations is denoted as ZORA-xc/TZ2P-ae // ZORA-OPBE/TZ2P, and along the manuscript it will be referred to as xc // OPBE.

Extended Benchmark Discussion

The performances of DFT in reproducing CCSD(T) trends were tested as described in the additional computational details. The activation and reaction energies obtained with DFT employing the five preliminary functionals were then compared to the CCSD(T) computed reference values. The results are shown in the Table S2, while the deviation from CCSD(T) results is represented in Figure S1. While all five functionals recover the trends discussed for CCSD(T), with the exception of the heightening of the activation energy going from Te (+2) to Te (+4) which is recovered only by OPBE and M06-2X, it can be clearly seen that the cheaper functionals (i.e. GGAs or the dispersion corrected GGA) underestimate the activation energy for the reaction of sulfoxides, selenoxides and telluroxides, with BLYP-D3(BJ) providing the worst results, with errors larger than -15 kcal mol^{-1} in some cases. (Figure S1)

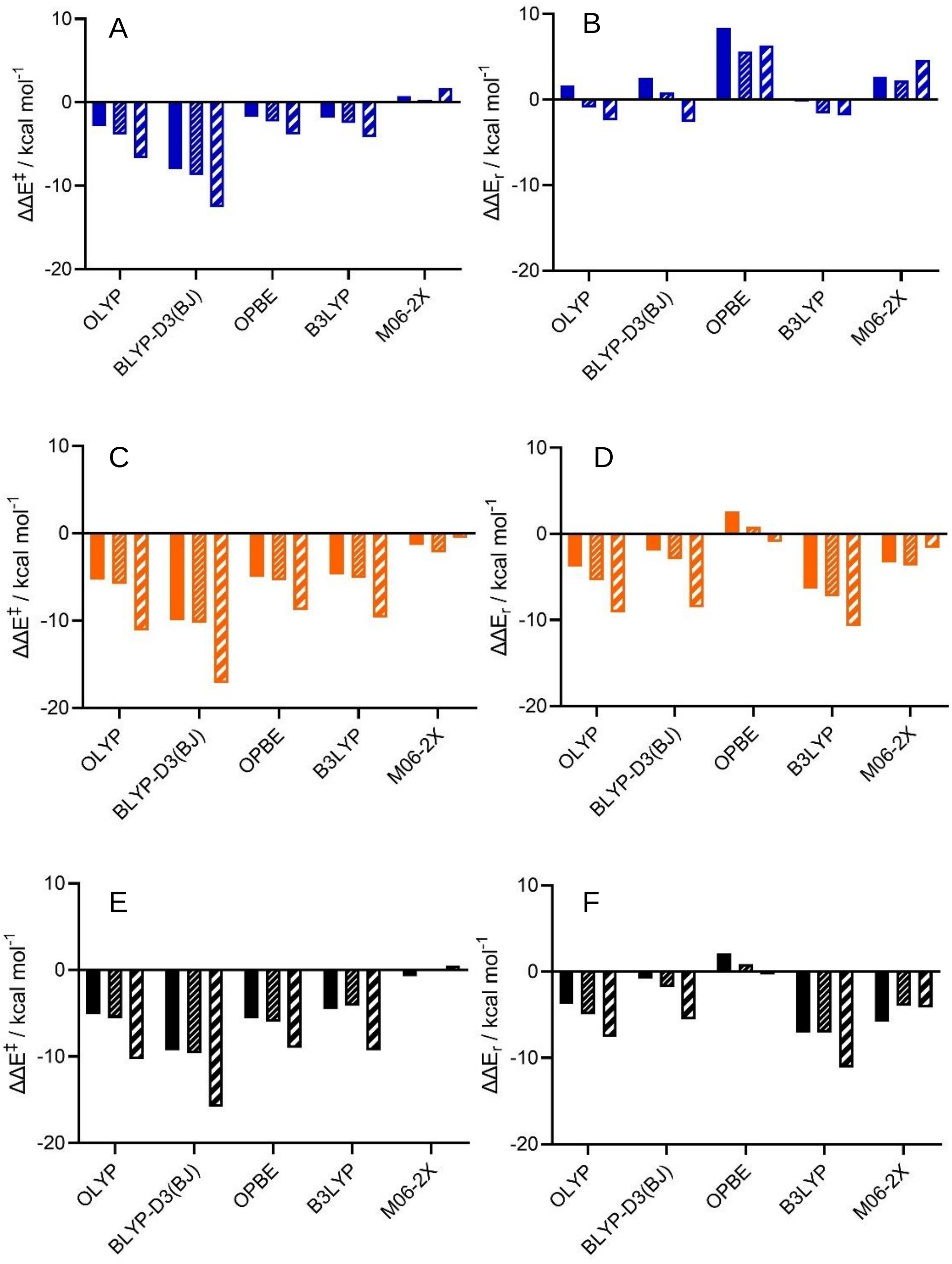

Figure S1. Deviation of the activation ($\Delta\Delta E^{\ddagger}$, A, C, E) and reaction ($\Delta\Delta E_r$, B, D, F) energies predicted at DFT (ZORA-xc/TZ2P) level of theory from CCSD(T) reference values. A negative value means that DFT underestimates the energy, while a positive value means that DFT overestimates the energy. Data are grouped on chalcogen basis: sulfur (A/B, blue), selenium (C/D, orange), tellurium (E/F, black). Bar filling is used to denote the OS: the lowest OS (0) is in solid color, the intermediate OS (+2) is dashed (thin lines), while the highest (+4) is dashed (thick lines).

For the other GGAs and B3LYP, the error generally increases going from S to Se, and from the lowest to the highest OS, with all the reactions in the OS +4 systematically displaying the strongest deviations from the CCSD(T) activation energies. The situation is rather different for M06-2X activation energies, that agree almost perfectly with the highly-correlated single points. With this functional, no great error arises when going from the OS 0 to the OS +4, and the $\Delta E^{\ddagger}$ of reactions involving Se shows deviation only slightly larger than those involving S.

A somewhat different picture describes deviations in reaction energies. In this case, the performance of the functionals appears to be somewhat less systematic, with some changes with the chalcogen and with the OS. Particularly, OPBE functional seems to be the worst performer for reactions involving S, but is the best performer for reactions involving Se. On the other hand, while B3LYP appears to be the worst performer for Se and Te, it is the best performer for S, with OLYP and BLYP-D3(BJ) giving similar results. In this case, M06-2X neither excels nor completely fails, predicting reaction energies within ca. ±5 kcal mol^{-1} with respect to CCSD(T), and always with the correct qualitative trend.

Considering these results, OLYP and OPBE functionals, benchmarked and popularly used to study S_N2 reactions[4,26,27] (such as chalcogenide oxidations[28,29]) and E2 reactions,[30,31] do not perform equally well for the quantitative description of chalcogenoxide elimination activation energies, even if they can still be used with some caution to understand the trends in the energetics in analogous elimination reactions, since CCSD(T) trends in activation and reaction energies are properly recovered also with the cheapest GGA or dispersion corrected GGA functionals.

In this preliminary analysis, OPBE appears to be the best performing GGA (Figure S1). OPBE functional was already found to perform very well for geometry optimization of organochalcogenides.[32]

Thus, starting from OPBE optimized geometries, 17 functionals were tested for single-point energy calculations as described. Activation and reaction energies computed at xc // OPBE level of theory clearly show that no cheap GGA functional can properly describe the title reactions. (Table S3) In contrast, the hybrid OPBE0, and the metrahybrid M06 and M06-2X provides good to excellent performances. (Figure S2)

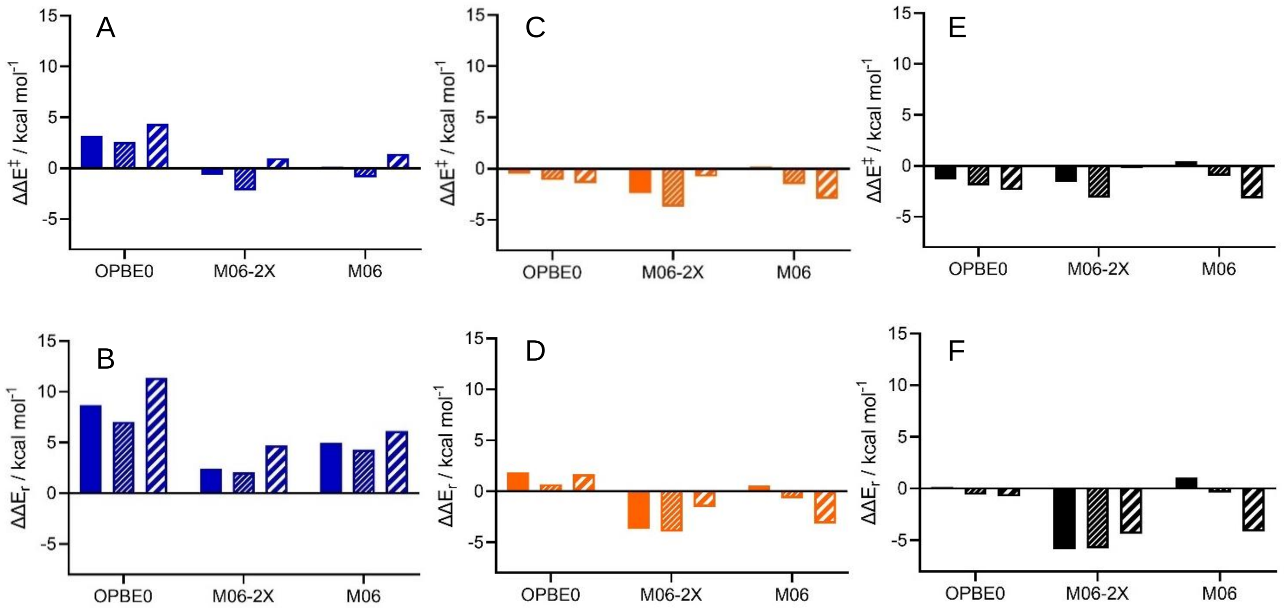

Figure S2. Deviation of the activation ($\Delta\Delta E^{\ddagger}$, A, C, E) and reaction ($\Delta\Delta E_r$, B, D, F) energies predicted at DFT (xc // OPBE) level of theory from CCSD(T) reference values. A negative value means that DFT underestimates the energy, while a positive value means that DFT overestimates the energy. Data are grouped on chalcogen basis: sulfur (A/B, blue), selenium (C/D, orange), tellurium (E/F, black). Bar filling is used to denote the OS: the lowest OS (0) is in solid color, the intermediate OS (+2) is dashed (thin lines), while the highest (+4) is dashed (thick lines).

Particularly, M06 // OPBE appears to be the best performing protocol for investigating reactions in the lowest oxidation state (OS 0) regardless of the chalcogen involved, while M06-2X // OPBE is the best approach to compute the activation energies in the highest OS (+4) for all chalcogens. OPBE0 // OPBE, on the other hand, gives a quite satisfying description of all Se reactions, with errors in reaction energies below 2.0 kcal mol^{-1} and errors in activation energies below 1.5 kcal mol^{-1} for all OSs.

Moreover, all three protocols predict activation and reaction energies that correlate very well against CCSD(T) ones, (Figure S3) with very similar R^2 values in the range 0.97-0.99 for both activation and reaction energies and mean absolute errors of ca. 2.00 kcal mol^{-1} or lower for activation energies and between 2 – 4 kcal mol^{-1} for reaction energies. Thus, in our opinion, all these three approaches can be employed to investigate the title reaction since the trends are qualitatively and quantitatively reproduced with the hybrid (OPBE0) as well as with the two meta-hybrids (M06 and M06-2X) functionals, and each of the three functionals outperforms the other two in a specific subset of reactions, with M06 being in average the best among the three.

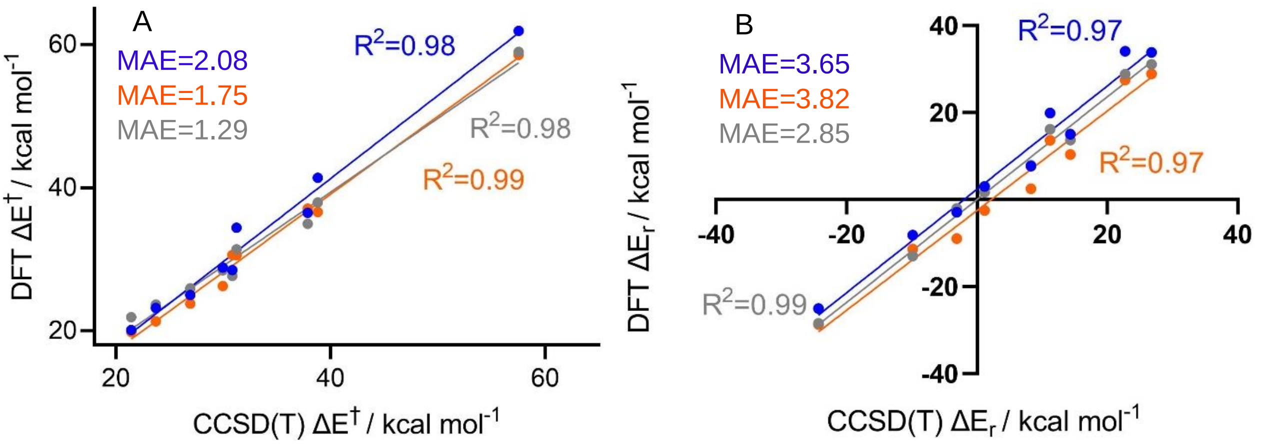

Figure S3. Correlation between xc // OPBE and CCSD(T) activation (A) and reaction energies (B). Blue dots: OPBE0 // OPBE; orange dots: M06-2X // OPBE; grey dots: M06 // OPBE. Statistical parameters (MAE: Mean Absolute Error, and R^2) are reported near the linear fit.

For our ongoing investigation, we employ M06 // OPBE (main text) and M06-2X // OPBE as main methods.

Table S1. Activation and reaction electronic energies (kcal mol^{-1}) for the β-elimination reaction of chalcogenoxides (OS 0), chalcogeninic acids (OS +2) and chalcogenonic acids (OS +4). Level of theory: ZORA-OPBE/TZP-ae; TZ2P(-ae); QZ4P-ae.

basis	OS	S	Se	Te	S	Se	Te
TZP-ae	0	28.97	17.65	13.42	19.33	2.74	-5.37
	+2	35.91	23.28	18.15	31.89	13.91	4.48
	+4	53.00	27.66	20.71	28.96	-11.78	-27.20
			$\Delta E^{\ddagger}$			ΔE_r	
basis	OS	S	Se	Te	S	Se	Te
TZ2P-ae	0	29.42	18.85	16.14	19.70	4.14	-0.74
	+2	36.46	24.69	21.24	32.47	15.61	9.38
	+4	53.60	28.99	21.98	29.29	-10.79	-25.01
			$\Delta E^{\ddagger}$			ΔE_r	
basis	OS	S	Se	Te	S	Se	Te
TZ2P-sc	0	29.49	18.73	15.84	19.60	3.75	-0.94
	+2	36.50	24.59	20.96	32.41	15.16	9.17
	+4	53.71	29.05	21.86	29.08	-10.74	-24.64
			$\Delta E^{\ddagger}$			ΔE_r	
basis	OS	S	Se	Te	S	Se	Te
QZ4P-ae	0	30.77	19.17	16.66	21.28	4.16	0.00
	+2	37.66	24.91	21.77	33.73	15.31	10.00
	+4	54.58	29.14	22.32	30.01	-11.01	-24.74

Increasing the basis set from TZP to TZ2P leads to recovering 1-2 kcal mol^{-1} in activation energy and has a somewhat more relevant impact on reaction energies, which are affected in the 1 – 4 kcal mol^{-1} range. Furtherly increasing the basis set to QZ4P leads to energetics which differs from those obtained with TZ2P of only fractions of kcal mol^{-1}. Moreover, the (small) frozen core approximation

as applied to TZ2P basis set does not seem to affect the energetics, with deviations from the TZ2P-ae are only a few fractions of kcal mol^{-1}. Thus, TZ2P basis set is deemed to be a reasonable compromise for the computation of chalcogenoxide elimination reactions, when small core approximation is available.

Table S2. Activation and reaction electronic energies (kcal mol^{-1}) for the for the β-elimination reaction of chalcogenoxides (minimal model) in OS 0, +2, +4. Level of theory: ZORA-xc/TZ2P(-ae)

		$\Delta E^{\ddagger}$			ΔE_r		
xc	OS	S	Se	Te	S	Se	Te
OLYP	0	28.39	18.38	16.27	12.91	-2.63	-6.82
	2	34.97	24.13	21.38	25.89	8.95	3.30
	4	50.85	26.71	20.53	20.31	-18.91	-31.83
		$\Delta E^{\ddagger}$			ΔE_r		
xc	OS	S	Se	Te	S	Se	Te
BLYP-D3(BJ)	0	23.22	13.75	12.16	13.77	-0.78	-3.87
	2	30.09	19.70	17.32	27.63	11.40	6.49
	4	44.95	20.70	15.07	20.17	-18.32	-29.82
		$\Delta E^{\ddagger}$			ΔE_r		
xc	OS	S	Se	Te	S	Se	Te
OPBE	0	29.49	18.73	15.84	19.60	3.75	-0.94
	2	36.50	24.59	20.96	32.41	15.16	9.17
	4	53.71	29.05	21.86	29.08	-10.74	-24.64
		$\Delta E^{\ddagger}$			ΔE_r		
xc	OS	S	Se	Te	S	Se	Te
B3LYP	0	29.36	19.00	16.88	11.00	-5.18	-10.12
	2	36.31	24.81	22.77	25.19	7.13	1.22
	4	53.38	28.23	21.58	20.91	-20.49	-35.39
		$\Delta E^{\ddagger}$			ΔE_r		
xc	OS	S	Se	Te	S	Se	Te
M06-2X	0	31.99	22.36	20.71	13.92	-2.16	-8.87
	2	38.77	27.78	27.06	29.04	10.69	4.29
	4	59.24	37.36	31.34	27.37	-11.48	-28.48

Table S3. Activation and reaction electronic energies (kcal mol^{-1}) for the β-elimination reaction of chalcogenoxides (OS 0), chalcogeninic acids (OS +2) and chalcogenonic acids (OS +4). Level of theory: xc // OPBE.

		$\Delta E^{\ddagger}$				ΔE_r	
xc	OS	S	Se	Te	S	Se	Te
BLYP	0	24.01	15.05	13.79	9.21	-5.51	-9.23
	+2	30.05	20.6	18.72	22.52	6.39	0.87
	+4	45.43	21.71	16.45	14.54	-24.50	-36.51
		$\Delta E^{\ddagger}$				ΔE_r	
xc	OS	S	Se	Te	S	Se	Te

BP86	0	24.10	14.35	12.37	15.85	0.77	-3.50
	+2	30.87	20.14	17.39	29.29	12.69	6.72
	+4	47.17	22.69	16.45	23.10	-16.68	-29.74
		$\Delta E^{‡}$				ΔE_r	
xc	OS	S	Se	Te	S	Se	Te
HTBS	0	24.52	14.30	11.96	18.25	2.95	-1.61
	+2	31.50	20.11	16.97	31.42	14.71	8.57
	+4	47.97	23.29	16.78	26.39	-13.67	-27.07
		$\Delta E^{‡}$				ΔE_r	
xc	OS	S	Se	Te	S	Se	Te
PBE	0	24.85	14.87	12.69	18.71	3.49	-0.86
	+2	31.81	20.76	17.78	32.27	15.54	9.48
	+4	48.29	23.68	17.22	26.28	-13.62	-26.82
		$\Delta E^{‡}$				ΔE_r	
xc	OS	S	Se	Te	S	Se	Te
mPW	0	24.91	15.17	13.36	16.04	0.99	-3.12
	+2	31.60	20.94	18.28	29.39	12.85	7.04
	+4	47.84	23.36	17.23	23.21	-16.59	-29.41
		$\Delta E^{‡}$				ΔE_r	
xc	OS	S	Se	Te	S	Se	Te
PW91	0	24.65	14.62	12.48	18.75	3.44	-0.88
	+2	31.59	20.49	17.56	32.36	15.52	9.48
	+4	48.08	23.31	16.87	26.25	-13.91	-27.13
		$\Delta E^{‡}$				ΔE_r	
xc	OS	S	Se	Te	S	Se	Te
revPBE	0	25.85	16.42	14.60	14.40	-0.32	-4.35
	+2	32.31	22.12	19.59	27.43	11.34	5.67
	+4	48.25	24.45	18.57	21.28	-17.59	-30.05
		$\Delta E^{‡}$				ΔE_r	
xc	OS	S	Se	Te	S	Se	Te
RPBE	0	26.14	16.81	15.04	14.20	-0.45	-4.41
	+2	32.52	22.48	20.04	27.22	11.22	5.62
	+4	48.35	24.74	18.95	20.86	-17.76	-30.08
		$\Delta E^{‡}$				ΔE_r	
xc	OS	S	Se	Te	S	Se	Te
mPBE	0	25.20	15.40	13.34	17.49	2.43	-1.81
	+2	32.01	21.24	18.41	30.92	14.38	8.45
	+4	48.31	23.96	17.68	24.81	-14.75	-27.71
		$\Delta E^{‡}$				ΔE_r	
xc	OS	S	Se	Te	S	Se	Te
TPSS	0	26.29	16.96	15.53	16.01	1.56	-1.89
	+2	32.75	22.73	20.61	29.33	13.34	8.10
	+4	49.50	25.22	19.18	24.28	-14.58	-27.03
		$\Delta E^{‡}$				ΔE_r	
xc	OS	S	Se	Te	S	Se	Te
SCAN	0	28.89	18.30	15.77	22.22	6.15	1.40
	+2	36.70	24.42	21.13	37.87	19.39	12.48
	+4	56.44	28.65	20.27	35.19	-8.54	-24.51
		$\Delta E^{‡}$				ΔE_r	

xc	OS	S	Se	Te	S	Se	Te
PBE0	0	31.00	20.14	17.39	19.15	2.37	-3.24
	+2	37.82	25.82	22.29	33.69	14.85	7.55
	+4	57.98	32.42	24.74	31.93	-10.44	-26.76
			$\Delta E^{\ddagger}$			ΔE_r	
xc	OS	S	Se	Te	S	Se	Te
OPBE0	0	34.39	23.18	20.08	19.92	3.02	-2.93
	+2	41.37	28.84	25.00	33.84	15.04	7.67
	+4	61.88	36.47	28.47	34.11	-8.14	-25.06
			$\Delta E^{\ddagger}$			ΔE_r	
xc	OS	S	Se	Te	S	Se	Te
mPW1PW	0	31.08	20.41	17.87	16.98	0.32	-5.11
	+2	37.73	25.98	22.71	31.34	12.66	5.53
	+4	57.60	32.16	24.75	27.47	-11.35	-28.68
			$\Delta E^{\ddagger}$			ΔE_r	
xc	OS	S	Se	Te	S	Se	Te
M06-2X	0	30.57	21.31	19.81	13.66	-2.51	-8.95
	+2	36.60	26.23	23.76	28.88	10.38	2.51
	+4	58.52	37.07	30.59	27.47	-11.35	-28.68
			$\Delta E^{\ddagger}$			ΔE_r	
xc	OS	S	Se	Te	S	Se	Te
M06	0	31.36	23.66	21.89	16.17	1.71	-2.03
	+2	37.91	28.42	25.92	31.10	13.67	7.88
	+4	58.93	34.93	27.65	28.86	-12.99	-28.45
			$\Delta E^{\ddagger}$			ΔE_r	
xc	OS	S	Se	Te	S	Se	Te
TPSSh	0	28.65	18.80	17.15	16.33	1.14	-2.90
	+2	35.11	24.59	22.18	30.07	13.14	7.33
	+4	53.29	28.61	22.04	26.60	-13.39	-27.15
			$\Delta E^{\ddagger}$			ΔE_r	
xc	OS	S	Se	Te	S	Se	Te
BP86-D3(BJ)	0	23.84	14.12	12.09	19.29	4.49	0.74
	+2	30.54	19.88	17.11	33.09	16.71	11.21
	+4	47.50	22.87	16.46	27.55	-12.14	-24.94

Table S4. Activation ($\Delta E^{\ddagger}$) and reaction (ΔE_r) energies (kcal mol^{-1}) for the β-elimination reaction of chalcogenoxides (OS 0), chalcogeninic acids (OS +2) and chalcogenonic acids (OS +4).[a]

	OS	Configuration	$\Delta E^{\ddagger}$	ΔE_r
Cys	0	RR	21.82 (22.34)	5.13 (6.65)
		RS	28.21 (28.34)	10.43 (11.59)
	+ 2	RR	28.97 (30.27)	20.92 (22.76)
		RS	31.54 (32.51)	22.03 (23.70)
	+ 4	R	52.51 (52.73)	17.59 (18.87)
Sec	0	RR	17.53 (18.54)	-6.05 (-2.86)
		RS	19.95 (20.81)	-4.33 (-1.35)
	+ 2	RR	19.96 (21.36)	4.17 (7.17)
		RS	24.96 (25.85)	6.85 (9.44)
	+ 4	R	32.52 (30.10)	-21.72 (-23.04)
Tec	0	RR	17.45 (17.81)	-10.61 (-4.66)
		RS	18.21 (18.28)	-9.83 (-4.20)
	+ 2	RR	19.00 (19.13)	-1.04 (3.72)
		RS	27.44 (26.83)	4.42 (8.51)
	+ 4	R	28.01 (24.82)	-35.58 (-35.32)

[a]Electronic energies computed at M06-2X // OPBE (M06 // OPBE) level of theory.

Table S5. Electronic energies (kcal mol^{-1}) for the cysteine, selenocysteine and tellurocysteine chalcogenoxide elimination in OS 0, +2, +4. Level of theory: M06 // OPBE.

		R	TS	P	DHA
Cys 0	RR	-2415.92	-2393.58	-515.03	-1894.24
	RS	-2420.86	-2392.52		
Sec 0	RR	-2387.41	-2368.87	-496.03	
	RS	-2388.92	-2368.11		
Tec 0	RR	-2365.99	-2348.18	-476.41	
	RS	-2366.45	-2348.17		
Cys 2	RR	-2642.94	-2612.67	-725.94	
	RS	-2643.88	-2611.37		
Sec 2	RR	-2610.93	-2589.57	-709.52	
	RS	-2613.20	-2587.35		
Tec 2	RR	-2596.19	-2577.06	-698.23	
	RS	-2574.15	-2574.15		
Cys 4	R	-2866.97	-2814.24	-953.86	
Sec 4	R	-2790.65	-2760.55	-919.45	
Tec 4	R	-2768.99	-2744.17	-910.07	

Table S6: Activation and reaction Gibbs free energies (kcal mol^{-1}) for the minimal model reactions. Level of theory: M06 // OPBE.

	$\Delta G^{\ddagger}$			ΔGr		
OS	S	Se	Te	S	Se	Te
0	28.27	21.31	19.92	2.88	-10.81	-14.07
+2	35.09	26.34	24.86	18.01	1.67	-2.91
+4	53.54	31.29	25.41	13.19	-27.33	-41.00

Table S7: Activation Gibbs free energies (kcal mol^{-1}) relative to the direct elimination mechanism of PhXEt ($\Delta G^{\ddagger}_{\mathrm{elm}}$), to the elimination mechanism of their hydrates ($\Delta G^{\ddagger}_{\mathrm{hyd,elm}}$), and reaction energies for the hydrates formation ($\Delta G_{\mathrm{r}}^{\mathrm{hyd}}$). Level of theory: M06 // OPBE.

	$\Delta G^{\ddagger}_{\mathrm{elm}}$	ΔG_{r}^{hyd}	$\Delta G^{\ddagger}_{\mathrm{hyd,elm}}$
PhSEt	28.42	33.51	29.57
PhSeEt	20.74	10.69	30.15
PhTeEt	19.33	-8.36	35.65

Table S8: Hirshfeld partial charges (a.u.) on the X = O oxygen atom of chalcogenoxides in different oxidation states.

	S	Se	Te
0	-0.373	-0.428	-0.471
2	-0.349	-0.400	-0.452
4	-0.294	-0.347	-0.398

Table S9: Cartesian coordinates (Å), energies (a.u.) and imaginary frequencies (cm^{-1}) of the optimized structures (minimal model). Level of theory: ZORA-M06-2X/TZ2P-ae.

HSOEt

E = -2.603634
G = -2.551961
N_{imag} = 0

S	0.900470000	-0.184160000	-0.654887000
C	0.773510000	-0.011223000	-2.457782000
H	-0.072061000	0.644721000	-2.657713000
H	1.695138000	0.470824000	-2.788091000
C	0.577903000	-1.385900000	-3.076558000
H	-0.312222000	-1.857525000	-2.663697000
H	1.433965000	-2.030691000	-2.878903000
H	0.454829000	-1.301054000	-4.154679000
O	-0.425308000	-0.630228000	-0.156605000
H	0.972478000	1.171434000	-0.482578000

HSO$_2$Et

E = -3.052357
G = -2.996529
N_{imag} = 0

S	-1.124629000	-0.504037000	0.186030000
O	-1.520805000	-0.499162000	-1.415817000
O	-0.702374000	0.839479000	0.572283000
H	-0.711607000	-0.603706000	-1.930638000
C	-2.852318000	-0.642875000	0.674535000
H	-2.828532000	-0.495542000	1.753777000
H	-3.344863000	0.213947000	0.214969000
C	-3.467261000	-1.975666000	0.282263000
H	-2.891588000	-2.808361000	0.686431000
H	-3.508102000	-2.077477000	-0.799594000
H	-4.480043000	-2.048362000	0.674325000

HSO$_3$Et

E = -3.495405
G = -3.434436
N_{imag} = 0

S	-0.799121000	-0.749125000	2.006285000
O	-1.043911000	-0.405773000	0.646639000
O	-0.036344000	-2.154182000	2.003651000
O	-1.869874000	-0.858903000	2.949517000
C	0.450302000	0.330123000	2.654210000
H	-0.055632000	1.292175000	2.733276000
H	0.667288000	-0.030845000	3.657766000
C	1.677051000	0.392606000	1.758300000
H	1.402211000	0.728661000	0.761347000
H	2.149107000	-0.584449000	1.678979000
H	2.397128000	1.091711000	2.178402000
H	-0.440182000	-2.716502000	2.679175000

HSeOEt

E = -2.538755

HSeO$_2$Et

E = -2.986216

G = -2.491158
N_{imag} = 0

Se	0.959934000	-0.195083000	-0.561664000
C	0.808951000	-0.017521000	-2.511492000
H	-0.039212000	0.642644000	-2.673934000
H	1.722123000	0.456747000	-2.868766000
C	0.579147000	-1.397469000	-3.102481000
H	-0.287790000	-1.863391000	-2.635934000
H	1.444217000	-2.042987000	-2.950857000
H	0.395030000	-1.324419000	-4.173308000
O	-0.552304000	-0.667605000	-0.108840000
H	0.968606000	1.295283000	-0.384219000

G = -2.934661
N_{imag} = 0

Se	-1.006601000	-0.543738000	0.226215000
O	-1.481775000	-0.584576000	-1.513579000
O	-0.637554000	0.996920000	0.563603000
H	-0.689368000	-0.474632000	-2.052054000
C	-2.878737000	-0.680076000	0.728042000
H	-2.878329000	-0.536543000	1.807486000
H	-3.325087000	0.192272000	0.254235000
C	-3.508612000	-1.994321000	0.300353000
H	-2.999019000	-2.847189000	0.748759000
H	-3.476856000	-2.102185000	-0.781511000
H	-4.550181000	-2.027692000	0.617014000

$HSeO_3Et$

E = -3.366323
G = -3.311441
N_{imag} = 0

Se	-0.856465000	-0.749880000	2.007708000
O	-1.138848000	-0.409628000	0.477055000
O	0.021145000	-2.268574000	2.057598000
O	-2.055793000	-0.907860000	3.054301000
C	0.512947000	0.415460000	2.687088000
H	0.015985000	1.381286000	2.758182000
H	0.718405000	0.037078000	3.685783000
C	1.717233000	0.422534000	1.761093000
H	1.429529000	0.736019000	0.760133000
H	2.169003000	-0.565373000	1.703054000
H	2.458976000	1.119661000	2.146785000
H	-0.494095000	-2.875226000	2.608765000

HTeOEt

E = -2.499384
G = -2.454722
N_{imag} = 0

Te	1.036274000	-0.184800000	-0.436996000
C	0.854424000	-0.028723000	-2.567823000
H	0.023566000	0.653906000	-2.729271000
H	1.766142000	0.412890000	-2.965387000
C	0.564849000	-1.410832000	-3.137056000
H	-0.302547000	-1.847973000	-2.645002000
H	1.412206000	-2.083174000	-3.003823000
H	0.354725000	-1.346410000	-4.203943000
O	-0.624689000	-0.770385000	-0.017842000

$HTeO_2Et$

E = -2.960916
G = -2.911544
N_{imag} = 0

Te	-0.809819000	-0.648776000	0.331982000
O	-1.084078000	-0.908492000	-1.574716000
O	-0.513330000	1.116862000	0.415060000
H	-1.143725000	-0.039176000	-1.994155000
C	-2.891872000	-0.714627000	0.765260000
H	-2.988405000	-0.556907000	1.839042000
H	-3.281503000	0.167011000	0.257344000
C	-3.549939000	-2.007729000	0.302688000
H	-3.147569000	-2.872915000	0.828951000

H	0.913752000	1.491700000	-0.264354000

H	-3.399699000	-2.158832000	-0.764628000
H	-4.622182000	-1.978178000	0.491739000

$HTeO_3Et$

E = -3.327297
G = -3.276403
N_{imag} = 0

Te	-0.961693000	-0.755429000	2.007580000
O	-1.260701000	-0.411323000	0.292624000
O	0.034315000	-2.386289000	2.093237000
O	-2.293897000	-0.956306000	3.167356000
C	0.569451000	0.502363000	2.713275000
H	0.118268000	1.490888000	2.775926000
H	0.786473000	0.145546000	3.717889000
C	1.766243000	0.450914000	1.770415000
H	1.482908000	0.752341000	0.764363000
H	2.187156000	-0.551258000	1.726887000
H	2.536640000	1.130328000	2.130922000
H	-0.467141000	-3.076277000	2.547073000

Transition states

$HSOEt^‡$

E = -2.552654
G = -2.506113
N_{imag} = -1196.554

S	0.615531000	-0.607256000	-0.681285000
C	0.697727000	-0.054457000	-2.925318000
H	-0.100085000	0.675277000	-2.909885000
H	1.688846000	0.376535000	-2.903212000
C	0.493121000	-1.325894000	-3.483676000
H	0.417918000	-2.017240000	-2.311994000
H	1.336845000	-1.805803000	-3.964033000
H	-0.471932000	-1.529778000	-3.931415000
O	0.417718000	-2.094233000	-1.065913000
H	-0.655488000	-0.157350000	-0.552668000

$HSO_2Et^‡$

E = -2.990574
G = -2.939643
N_{imag} = -1151.962

S	-0.759711000	-0.377310000	0.043980000
O	-0.289779000	-0.324263000	1.616145000
O	-0.703859000	-1.850612000	-0.363551000
H	0.674146000	-0.318069000	1.661140000
C	-2.969043000	-0.691636000	0.693802000
H	-2.766271000	-0.313052000	1.687541000
H	-3.422916000	0.022787000	0.020409000
C	-3.105107000	-2.059280000	0.466016000
H	-1.800732000	-2.239934000	-0.062851000
H	-3.722955000	-2.389116000	-0.359275000
H	-3.048669000	-2.731315000	1.312943000

$HSO_3Et^‡$

E = -3.401005
G = -3.348429
N_{imag} = -1452.626

S	-1.121716000	-0.167994000	-0.044338000
O	-1.329719000	0.580395000	-1.459338000
O	-0.818756000	-1.591335000	-0.430011000
H	-0.500974000	1.029954000	-1.688517000
C	-3.231352000	-0.794823000	0.807696000
H	-3.037978000	-0.371990000	1.784886000
H	-3.771684000	-0.145310000	0.130837000
C	-3.144191000	-2.164930000	0.579540000
H	-1.891196000	-2.102664000	0.004859000
H	-3.719920000	-2.581868000	-0.237186000
H	-2.967038000	-2.813238000	1.428087000
O	-0.026775000	0.507704000	0.620085000

HSeOEt‡

E = -2.503116

G = -2.459801

N_{imag} = -1241.307

Se	0.664109000	-0.571446000	-0.610955000
C	0.710185000	-0.050913000	-2.928678000
H	-0.084023000	0.683063000	-2.927525000
H	1.700356000	0.383202000	-2.951155000
C	0.491178000	-1.338872000	-3.474797000
H	0.419965000	-2.038763000	-2.360044000
H	1.327724000	-1.796798000	-3.989381000
H	-0.474186000	-1.514190000	-3.935125000
O	0.426609000	-2.184802000	-1.069193000
H	-0.741719000	-0.110682000	-0.482546000

$HSeO_2Et$‡

E = -2.941943

G = -2.894133

N_{imag} = -1259.306

Se	-0.890089000	-0.359284000	0.091687000
O	-0.243308000	-0.328175000	1.763543000
O	-0.916023000	-1.988474000	-0.290897000
H	0.714349000	-0.434122000	1.729770000
C	-3.058325000	-0.682793000	0.993777000
H	-2.767757000	-0.298757000	1.964008000
H	-3.607524000	0.021468000	0.382825000
C	-3.221027000	-2.066609000	0.797074000
H	-2.043265000	-2.309460000	0.159947000
H	-3.961463000	-2.386733000	0.074609000
H	-3.112266000	-2.706858000	1.663957000

$HSeO_3Et$‡

E = -3.306781

G = -3.258153

N_{imag} = -1362.078

Se	-1.003311000	-0.235649000	-0.024539000
O	-1.397199000	0.613808000	-1.526038000
O	-0.824244000	-1.808182000	-0.505883000
H	-0.587499000	1.052100000	-1.827728000
C	-3.192342000	-0.739062000	0.907109000
H	-2.974879000	-0.393633000	1.909475000
H	-3.688365000	-0.013566000	0.273325000
C	-3.204300000	-2.110822000	0.589079000
H	-2.059542000	-2.209089000	0.019738000
H	-3.871686000	-2.410211000	-0.211414000
H	-3.130499000	-2.800528000	1.421781000
O	0.372565000	0.438736000	0.471695000

HTeOEt‡

E = -2.466376
G = -2.425441
N_{imag} = -1547.67

Te	0.717691000	-0.546993000	-0.502026000
C	0.730457000	-0.042527000	-2.951886000
H	-0.060766000	0.694299000	-2.965512000
H	1.716914000	0.393305000	-3.027228000
C	0.494446000	-1.346000000	-3.478873000
H	0.430229000	-2.070205000	-2.392538000
H	1.319123000	-1.794860000	-4.019812000
H	-0.476931000	-1.505007000	-3.932176000
O	0.441467000	-2.281772000	-1.096153000
H	-0.872431000	-0.040440000	-0.363196000

$HTeO_2Et$‡

E = -2.917792
G = -2.872636
N_{imag} = -1394.095

Te	0.169944000	0.425500000	-0.571995000
C	-0.526265000	2.034732000	-2.325361000
H	-1.000502000	2.705954000	-1.618683000
H	0.428740000	2.390838000	-2.686560000
C	-1.330276000	1.206658000	-3.150879000
H	-1.193761000	0.019378000	-2.525611000
H	-0.963982000	1.011174000	-4.150810000
H	-2.401701000	1.345391000	-3.075203000
O	-0.802496000	-0.753233000	-1.598220000
O	-1.226124000	1.207360000	0.543259000
H	-1.495078000	0.614649000	1.252263000

$HTeO_3Et$‡

E = -3.277349
G = -3.232507
N_{imag} = -2056.57

Te	-0.176370000	0.794425000	-0.425664000
C	-0.614611000	2.178559000	-2.513030000
H	-1.225913000	2.953491000	-2.070419000
H	0.429681000	2.444566000	-2.630255000
C	-1.190285000	1.136442000	-3.288725000
H	-1.168101000	0.155326000	-2.516600000
H	-0.573497000	0.786183000	-4.109662000
H	-2.240644000	1.262688000	-3.525661000
O	-0.878326000	-0.627460000	-1.323886000
O	1.719867000	0.542072000	-0.640598000
H	2.102670000	0.119757000	0.139982000
O	-0.405568000	0.668451000	1.340318000

Products

Ethylene

E = -1.573611
G = -1.543121
N_{imag} = 0

C	0.000000000	0.000000000	-0.660928000
C	0.000000000	0.000000000	0.660928000
H	0.000000000	0.922016000	-1.227432000
H	0.000000000	-0.922016000	-1.227432000
H	0.000000000	0.922016000	1.227432000
H	0.000000000	-0.922016000	1.227432000

H_2SO

E = -1.007834
G = -1.008353
N_{imag} = 0

S	0.551958000	-0.579004000	-1.948494000
H	0.719272000	-1.794830000	-2.494444000
O	-0.006992000	-1.061996000	-0.462723000
H	0.747986000	-1.147734000	0.128303000

H_2SO_2

E = -1.432473
G = -1.428474
N_{imag} = 0

S	2.111079000	-2.340436000	0.000000000
O	2.695473000	-1.507982000	-1.280660000
O	2.695473000	-1.507982000	1.280660000
H	3.549930000	-1.868426000	1.543830000
H	3.549930000	-1.868426000	-1.543830000

H_2SO_3

E = -1.878181
G = -1.872683
N_{imag} = 0

S	-0.226382000	0.247576000	-3.321460000
O	-0.025617000	-0.112960000	-4.880753000
O	0.836256000	1.451440000	-3.168841000
O	0.346999000	-0.867060000	-2.591711000
H	0.671838000	-0.784517000	-4.960688000
H	1.699147000	1.079709000	-2.921744000

H_2SeO

E = -0.968588
G = -0.972090
N_{imag} = 0

Se	0.556809000	-0.513043000	-2.002865000
H	0.739044000	-1.856659000	-2.571693000
O	-0.027648000	-1.058789000	-0.384688000
H	0.744018000	-1.155073000	0.181889000

H_2SeO_2

E = -1.811006
G = -1.808685
N_{imag} = 0

Se	2.042761000	-2.402939000	0.000000000
O	2.713800000	-1.484126000	-1.373406000
O	2.713800000	-1.484126000	1.373406000
H	3.565762000	-1.861030000	1.616124000
H	3.565762000	-1.861030000	-1.616124000

H_2SeO_3

E = -1.811006
G = -1.808685
N_{imag} = 0

Se	-0.298748000	0.269677000	-3.305146000
O	-0.031127000	-0.138815000	-4.998869000
O	0.900360000	1.552116000	-3.148517000
O	0.384811000	-0.957530000	-2.528119000
H	0.623250000	-0.854077000	-5.024308000
H	1.723696000	1.142818000	-2.840241000

H_2TeO

E = -0.939915
G = -0.945713
N_{imag} = 0

Te	0.550843000	-0.427819000	-2.064682000
H	0.767167000	-1.947558000	-2.697693000
O	-0.035752000	-1.045531000	-0.292091000
H	0.729965000	-1.162656000	0.277110000

H_2TeO_2

E = -1.380460
G = -1.381103
N_{imag} = 0

Te	1.945131000	-2.467882000	0.000000000
O	2.739107000	-1.469612000	-1.474585000
O	2.739107000	-1.469612000	1.474585000
H	3.589270000	-1.843073000	1.724039000
H	3.589270000	-1.843073000	-1.724039000

H_2TeO_3

E = -1.799078
G = -1.798901
N_{imag} = 0

Te	-0.392767000	0.290190000	-3.276755000
O	-0.041663000	-0.187374000	-5.101689000
O	0.963188000	1.637062000	-3.106271000
O	0.376759000	-1.064888000	-2.425289000
H	0.607079000	-0.904069000	-5.142015000
H	1.789645000	1.243264000	-2.793177000

Table S10: Cartesian coordinates (Å), energies (a.u.) and imaginary frequencies (cm^{-1}) of the optimized structures of the minimal model. Level of theory: ZORA-OPBE/TZ2P.

Reactants:

HSOEt

E = -1.838526
G = -1.789445
N_{imag} = 0

S	0.883283000	-0.189967000	-0.626942000
C	0.743459000	-0.038417000	-2.440107000
H	-0.106255000	0.621821000	-2.648643000
H	1.667705000	0.470048000	-2.749310000
C	0.585860000	-1.392523000	-3.100297000
H	-0.319143000	-1.897093000	-2.748645000
H	1.443506000	-2.042920000	-2.895737000
H	0.504384000	-1.275234000	-4.186363000
O	-0.435774000	-0.556839000	-0.048887000
H	1.031678000	1.187320000	-0.526563000

HSO_2Et

E = -2.105208
G = -2.052069
N_{imag} = 0

S	-1.113042000	-0.493863000	0.157285000
O	-1.498222000	-0.408033000	-1.465806000
O	-0.677254000	0.827407000	0.628222000
H	-0.670544000	-0.587350000	-1.933309000
C	-2.852085000	-0.657182000	0.645343000
H	-2.813462000	-0.489154000	1.728090000
H	-3.364758000	0.196831000	0.187539000
C	-3.481633000	-1.990445000	0.299552000
H	-2.915060000	-2.827409000	0.722591000
H	-3.550321000	-2.133455000	-0.782125000
H	-4.495739000	-2.039107000	0.711183000

HSO_3Et

E = -2.374253

G = -2.316030

N_{imag} = 0

S	-0.821418000	-0.764702000	1.999013000
O	-1.088881000	-0.422703000	0.633092000
O	-0.087569000	-2.206174000	1.975130000
O	-1.874186000	-0.838235000	2.980762000
C	0.460738000	0.304577000	2.629056000
H	-0.055222000	1.267623000	2.726320000
H	0.678213000	-0.057617000	3.639416000
C	1.697307000	0.410285000	1.760690000
H	1.445311000	0.753157000	0.754064000
H	2.218154000	-0.547785000	1.681939000
H	2.386993000	1.134938000	2.206403000
H	-0.461419000	-2.697866000	2.721659000

HSeOEt

E = -1.782561

G = -1.737707

N_{imag} = 0

Se	0.932794000	-0.208876000	-0.523842000
C	0.769100000	-0.048366000	-2.495690000
H	-0.091292000	0.608038000	-2.658274000
H	1.682425000	0.465123000	-2.822156000
C	0.593193000	-1.406396000	-3.136011000
H	-0.294021000	-1.914461000	-2.744987000
H	1.462089000	-2.052927000	-2.968589000
H	0.464705000	-1.299435000	-4.219562000
O	-0.573390000	-0.565494000	0.028662000
H	1.053099000	1.308993000	-0.431046000

$HSeO_2Et$

E = -2.048818

G = -2.00009

N_{imag} = 0

Se	-0.996671000	-0.552955000	0.174900000
O	-1.460770000	-0.413407000	-1.595937000
O	-0.571591000	0.931848000	0.683332000
H	-0.620353000	-0.420369000	-2.075381000
C	-2.894009000	-0.693349000	0.675387000
H	-2.871939000	-0.488760000	1.751759000
H	-3.357517000	0.158662000	0.168149000
C	-3.531141000	-2.023209000	0.339882000
H	-3.017276000	-2.859871000	0.826742000
H	-3.539999000	-2.205260000	-0.738431000
H	-4.570856000	-2.035092000	0.688162000

$HSeO_3Et$

E = -2.254115

G = -2.202215

N_{imag} = 0

Se	-0.873806000	-0.777028000	1.997589000
O	-1.177623000	-0.448940000	0.458327000
O	-0.022322000	-2.352731000	2.055030000
O	-2.059997000	-0.875668000	3.081581000
C	0.537586000	0.394909000	2.664499000
H	0.010884000	1.351372000	2.756695000
H	0.741540000	0.001368000	3.664945000

C	1.744636000	0.458670000	1.760623000
H	1.473517000	0.785631000	0.753530000
H	2.252722000	-0.506752000	1.692900000
H	2.453379000	1.185197000	2.175034000
H	-0.582494000	-2.880532000	2.646793000

HTeOEt

E = -1.752452
G = -1.710653
N_{imag} = 0

Te	0.992851000	-0.219434000	-0.386571000
C	0.789522000	-0.072048000	-2.543493000
H	-0.075568000	0.583896000	-2.684278000
H	1.688810000	0.446406000	-2.897786000
C	0.596729000	-1.427456000	-3.189793000
H	-0.285951000	-1.938081000	-2.790908000
H	1.463999000	-2.081752000	-3.044182000
H	0.450610000	-1.315610000	-4.271243000
O	-0.698845000	-0.582210000	0.155826000
H	1.076547000	1.492488000	-0.319066000

HTeO$_2$Et

E = -2.027944
G = -1.982966
N_{imag} = 0

Te	-0.847200000	-0.588340000	0.201166000
O	-1.375061000	-0.439238000	-1.707963000
O	-0.469531000	1.099638000	0.700132000
H	-0.569600000	-0.289138000	-2.220881000
C	-2.930875000	-0.735968000	0.713930000
H	-2.948510000	-0.514412000	1.787540000
H	-3.368283000	0.117407000	0.184389000
C	-3.591558000	-2.055703000	0.373662000
H	-3.125216000	-2.899534000	0.895021000
H	-3.559322000	-2.260000000	-0.700883000
H	-4.646964000	-2.036472000	0.672453000

HTeO$_3$Et

E = -2.221698215494857
G = -2.173802
N_{imag} = 0

Te	-0.968970000	-0.799592000	1.981356000
O	-1.271584000	-0.488636000	0.248993000
O	-0.036830000	-2.507810000	2.108654000
O	-2.305729000	-0.900382000	3.167688000
C	0.611196000	0.467243000	2.694013000
H	0.107372000	1.433017000	2.814123000
H	0.831707000	0.056659000	3.684776000
C	1.799290000	0.517871000	1.760963000
H	1.516962000	0.862078000	0.762224000
H	2.288003000	-0.455870000	1.667871000
H	2.534950000	1.223796000	2.165222000
H	-0.608343000	-3.072877000	2.651665000

Transition states

$HSOEt^{‡}$

E = -1.791535
G = -1.747369
N_{imag} = -690.977

S	0.621299000	-0.629890000	-0.633772000
C	0.697864000	-0.037448000	-2.982045000
H	-0.115589000	0.683163000	-2.934632000
H	1.693588000	0.395852000	-2.923345000
C	0.501666000	-1.310347000	-3.510141000
H	0.412713000	-1.973643000	-2.275596000
H	1.353234000	-1.838700000	-3.938566000
H	-0.471973000	-1.561513000	-3.931145000
O	0.404010000	-2.107664000	-1.100453000
H	-0.656612000	-0.160011000	-0.499706000

$HSO_2Et^{‡}$

E = -2.047034
G = -1.998381
N_{imag} = -436.564

S	-0.685762000	-0.365880000	0.062512000
O	-0.171914000	-0.289466000	1.633551000
O	-0.728487000	-1.873709000	-0.329342000
H	0.795532000	-0.305557000	1.609068000
C	-3.074621000	-0.709458000	0.718390000
H	-2.865399000	-0.332296000	1.716939000
H	-3.457448000	0.024035000	0.012187000
C	-3.175942000	-2.060522000	0.467108000
H	-1.770452000	-2.174606000	-0.035639000
H	-3.694325000	-2.411069000	-0.424739000
H	-3.086082000	-2.773272000	1.286265000

$HSO_3Et^{‡}$

E = -2.288666
G = -2.239037
N_{imag} = -1121.322

S	-1.114981000	-0.156582000	-0.066885000
O	-1.291575000	0.575923000	-1.521091000
O	-0.841714000	-1.613786000	-0.435259000
H	-0.465099000	1.067992000	-1.659909000
C	-3.272675000	-0.804296000	0.830611000
H	-3.050948000	-0.374020000	1.805832000
H	-3.823494000	-0.152308000	0.154823000
C	-3.159095000	-2.167675000	0.593992000
H	-1.901086000	-2.058192000	0.006129000
H	-3.717591000	-2.603301000	-0.234209000
H	-2.932204000	-2.827973000	1.430452000
O	0.009161000	0.498116000	0.592114000

$HSeOEt^{‡}$

E = -1.752708
G = -1.711613
N_{imag} = -794.737

Se	0.665809000	-0.590524000	-0.587056000
C	0.711446000	-0.038338000	-2.964664000
H	-0.091383000	0.695890000	-2.941865000
H	1.709678000	0.395504000	-2.961617000
C	0.495406000	-1.325681000	-3.489546000
H	0.420016000	-2.005794000	-2.329840000

$HSeO_2Et^{‡}$

E = -2.009636
G = -1.964222
N_{imag} = -719.861

Se	-0.853727000	-0.351775000	0.120258000
O	-0.145910000	-0.304093000	1.787404000
O	-0.947661000	-2.000379000	-0.264233000
H	0.809382000	-0.391837000	1.663256000
C	-3.127971000	-0.701591000	1.025251000
H	-2.846025000	-0.322610000	2.006089000

H	1.336861000	-1.823812000	-3.972154000
H	-0.479890000	-1.536768000	-3.929541000
O	0.421023000	-2.194991000	-1.103486000
H	-0.748767000	-0.115687000	-0.449632000

H	-3.627620000	0.022948000	0.384219000
C	-3.268703000	-2.070407000	0.796738000
H	-2.015483000	-2.259127000	0.170512000
H	-3.940110000	-2.405145000	0.006322000
H	-3.142922000	-2.755794000	1.634619000

HSeO3Et‡

E = -2.207825
G = -2.161719
N_{imag} = -935.661

Se	-1.026349000	-0.236525000	-0.056360000
O	-1.350339000	0.617639000	-1.613309000
O	-0.842781000	-1.837655000	-0.517384000
H	-0.516352000	1.083569000	-1.789383000
C	-3.215694000	-0.736815000	0.918215000
H	-2.958775000	-0.384995000	1.916848000
H	-3.743679000	-0.011793000	0.299510000
C	-3.206050000	-2.108298000	0.597056000
H	-2.070009000	-2.188894000	0.013468000
H	-3.884405000	-2.429688000	-0.195052000
H	-3.091051000	-2.806382000	1.427664000
O	0.344185000	0.423736000	0.495329000

HTeOEt‡

E = -1.727205
G = -1.688543
N_{imag} = -855.795

Te	0.712690000	-0.562841000	-0.486540000
C	0.735608000	-0.032269000	-2.976323000
H	-0.059552000	0.711295000	-2.976761000
H	1.733094000	0.401403000	-3.027263000
C	0.500201000	-1.332938000	-3.490459000
H	0.429214000	-2.040878000	-2.366431000
H	1.330313000	-1.820174000	-4.003811000
H	-0.480285000	-1.520996000	-3.930364000
O	0.430652000	-2.292341000	-1.131183000
H	-0.891735000	-0.050460000	-0.340264000

HTeO$_2$Et‡

E = -1.994550
G = -1.951254
N_{imag} = -848.914

Te	0.140122000	0.434115000	-0.537534000
C	-0.545892000	2.071376000	-2.371407000
H	-1.026145000	2.763285000	-1.679585000
H	0.445068000	2.388125000	-2.693671000
C	-1.323437000	1.227995000	-3.188395000
H	-1.179769000	0.039390000	-2.482858000
H	-0.924681000	0.948685000	-4.163662000
H	-2.407735000	1.327380000	-3.136574000
O	-0.838659000	-0.723701000	-1.619387000
O	-1.255641000	1.190396000	0.634852000
H	-1.424731000	0.541354000	1.330420000

HTeO$_3$Et‡

E = -2.186857
G = -2.142527
N_{imag} = -707.937

Te	-0.133355000	0.790972000	-0.457365000
C	-0.613903000	2.190528000	-2.535783000
H	-1.223461000	2.957062000	-2.058019000
H	0.425848000	2.477870000	-2.695535000
C	-1.207786000	1.135542000	-3.284937000
H	-1.159798000	0.163094000	-2.517298000
H	-0.620369000	0.781077000	-4.135019000
H	-2.275798000	1.244678000	-3.484641000
O	-0.875011000	-0.648689000	-1.336815000
O	1.791390000	0.463359000	-0.622751000
H	2.076579000	0.145683000	0.248477000
O	-0.405435000	0.713323000	1.315487000

Products

Ethylene

E = -1.174318
G = -1.145266
N_{imag} = 0

C	0.000000000	0.000000000	-0.664603000
C	0.000000000	0.000000000	0.664603000
H	0.000000000	0.927052000	-1.236331000
H	0.000000000	-0.927052000	-1.236331000
H	0.000000000	0.927052000	1.236331000
H	0.000000000	-0.927052000	1.236331000

H_2SO

E = -0.632976
G = -0.634126
N_{imag} = 0

S	0.556827000	-0.578855000	-1.938258000
H	0.721942000	-1.799811000	-2.503630000
O	-0.020912000	-1.061721000	-0.453121000
H	0.754368000	-1.143177000	0.117652000

H_2SO_2

E = -0.879244
G = -0.876005
N_{imag} = 0

S	2.134586000	-2.338611000	0.000000000
O	2.688384000	-1.500091000	-1.305462000
O	2.688384000	-1.500091000	1.305462000
H	3.545266000	-1.877229000	1.550700000
H	3.545266000	-1.877229000	-1.550700000

H_2SO_3

E = -1.153595
G = -1.149393
N_{imag} = 0

S	-0.215525000	0.244434000	-3.324082000
O	-0.035974000	-0.119626000	-4.908176000
O	0.844487000	1.478683000	-3.159023000
O	0.348044000	-0.879128000	-2.581139000
H	0.662837000	-0.794640000	-4.963962000
H	1.698372000	1.084466000	-2.908817000

H_2SeO

E = -0.602264

H_2SeO_2

E = -0.850341

G = -0.606432

N_{imag} = 0

Se	0.562856000	-0.512191000	-1.992137000
H	0.741343000	-1.861514000	-2.577788000
O	-0.043925000	-1.059961000	-0.373562000
H	0.751950000	-1.149898000	0.166130000

G = -0.849786

N_{imag} = 0

Se	2.066271000	-2.402569000	0.000000000
O	2.706510000	-1.473766000	-1.402568000
O	2.706510000	-1.473766000	1.402568000
H	3.561297000	-1.871576000	1.615557000
H	3.561297000	-1.871576000	-1.615557000

H_2SeO_3

E = -1.096063

G = -1.808685

N_{imag} = 0

Se	-0.285588000	0.263833000	-3.306069000
O	-0.045796000	-0.151524000	-5.039445000
O	0.914325000	1.591683000	-3.132045000
O	0.384997000	-0.972738000	-2.514340000
H	0.611188000	-0.867544000	-5.030324000
H	1.723116000	1.150480000	-2.822976000

H_2TeO

E = -0.579634

G = -0.586078

N_{imag} = 0

Te	0.561225000	-0.425479000	-2.051028000
H	0.767415000	-1.953095000	-2.702048000
O	-0.057545000	-1.052803000	-0.278665000
H	0.741129000	-1.152187000	0.254386000

H_2TeO_2

E = -0.839019

G = -0.840300

N_{imag} = 0

Te	1.975002000	-2.472030000	0.000000000
O	2.732156000	-1.451505000	-1.502903000
O	2.732156000	-1.451505000	1.502903000
H	3.581286000	-1.859107000	1.715626000
H	3.581286000	-1.859107000	-1.715626000

H_2TeO_3

E = -1.086645

G = -1.087809

N_{imag} = 0

Te	-0.379951000	0.287821000	-3.281728000
O	-0.054260000	-0.192990000	-5.145951000
O	0.977635000	1.679738000	-3.097357000
O	0.389925000	-1.077136000	-2.419916000
H	0.592283000	-0.916644000	-5.126333000
H	1.776608000	1.233399000	-2.773912000

Table S11: Cartesian coordinates (Å), energies (a.u.) and imaginary frequencies (cm^{-1}) of the optimized structures of the minimal model. Level of theory: ZORA-OLYP/TZ2P

Reactants

HSOEt

E = -1.769872

G = -1.721144

N_{imag} = 0

S	0.886206000	-0.184592000	-0.611851000
C	0.746073000	-0.035847000	-2.446255000
H	-0.105186000	0.620950000	-2.650985000
H	1.668929000	0.470565000	-2.757633000

HSO_2Et

E = -2.027787

G = -1.975195

N_{imag} = 0

S	-1.097728000	-0.494797000	0.162208000
O	-1.489404000	-0.409920000	-1.479985000
O	-0.661419000	0.833566000	0.632390000
H	-0.658457000	-0.561960000	-1.955995000

C	0.585805000	-1.397845000	-3.106952000
H	-0.318178000	-1.901175000	-2.753851000
H	1.443663000	-2.047136000	-2.904033000
H	0.502880000	-1.280323000	-4.192163000
O	-0.442502000	-0.553283000	-0.037446000
H	1.031013000	1.194882000	-0.510325000

C	-2.857889000	-0.657089000	0.650361000
H	-2.823277000	-0.486920000	1.731361000
H	-3.366009000	0.193102000	0.184386000
C	-3.490466000	-1.997931000	0.304377000
H	-2.927227000	-2.833393000	0.732846000
H	-3.554955000	-2.143889000	-0.776147000
H	-4.505289000	-2.042529000	0.712762000

HSO_3Et

E = -2.281745

G = -2.224105

N_{imag} = 0

S	-0.832695000	-0.766307000	1.999798000
O	-1.099441000	-0.429897000	0.626102000
O	-0.084838000	-2.219553000	1.983688000
O	-1.891605000	-0.839925000	2.983712000
C	0.463675000	0.315494000	2.633278000
H	-0.049120000	1.278580000	2.727805000
H	0.682990000	-0.048987000	3.640635000
C	1.705770000	0.416636000	1.758190000
H	1.455161000	0.763954000	0.753866000
H	2.220570000	-0.543426000	1.677449000
H	2.397917000	1.136127000	2.206731000
H	-0.470362000	-2.727198000	2.716291000

HSeOEt

E = -1.714984

G = -1.670566

N_{imag} = 0

Se	0.937414000	-0.201800000	-0.504183000
C	0.770910000	-0.046524000	-2.504594000
H	-0.089177000	0.608042000	-2.664582000
H	1.684459000	0.463667000	-2.830268000
C	0.592648000	-1.412079000	-3.144946000
H	-0.294826000	-1.918133000	-2.754649000
H	1.460743000	-2.058173000	-2.976641000
H	0.465534000	-1.305315000	-4.228038000
O	-0.579896000	-0.565159000	0.049635000
H	1.050893000	1.321672000	-0.413228000

$HSeO_2Et$

E = -1.973795

G = -1.925701

N_{imag} = 0

Se	-0.977707000	-0.553044000	0.181514000
O	-1.448479000	-0.425732000	-1.612435000
O	-0.551928000	0.944647000	0.685827000
H	-0.609668000	-0.392802000	-2.098193000
C	-2.902570000	-0.693036000	0.682401000
H	-2.883823000	-0.488579000	1.757148000
H	-3.361315000	0.155221000	0.168448000
C	-3.540902000	-2.030098000	0.344000000
H	-3.028420000	-2.865280000	0.833222000
H	-3.546606000	-2.212204000	-0.733339000
H	-4.580703000	-2.040855000	0.689972000

$HSeO_3Et$

E = -2.165769

G = -2.114660

N_{imag} = 0

Se	-0.891470000	-0.779526000	1.999787000
O	-1.198008000	-0.459108000	0.447307000
O	-0.016841000	-2.368150000	2.069245000
O	-2.088947000	-0.875199000	3.088547000
C	0.545508000	0.411225000	2.670436000
H	0.020250000	1.366628000	2.757234000
H	0.748583000	0.014635000	3.667722000
C	1.755766000	0.465889000	1.759044000
H	1.486276000	0.796196000	0.753727000
H	2.256395000	-0.502321000	1.691002000
H	2.468718000	1.187065000	2.174420000
H	-0.588207000	-2.921837000	2.629074000

HTeOEt

E = -1.687642

G = -1.646228

N_{imag} = 0

Te	0.996033000	-0.212919000	-0.364435000
C	0.789761000	-0.071766000	-2.550728000
H	-0.075313000	0.581657000	-2.689917000
H	1.688862000	0.444623000	-2.903718000
C	0.597021000	-1.433761000	-3.200528000
H	-0.284642000	-1.945052000	-2.802991000
H	1.464999000	-2.085950000	-3.055623000
H	0.451515000	-1.319969000	-4.281172000
O	-0.709185000	-0.576255000	0.180166000
H	1.079652000	1.505590000	-0.302549000

$HTeO_2Et$

E = -1.956182

G = -1.909584

N_{imag} = 0

Te	-0.825587000	-0.583021000	0.213043000
O	-1.358650000	-0.471942000	-1.718323000
O	-0.456715000	1.126948000	0.688842000
H	-0.566821000	-0.268976000	-2.238425000
C	-2.937287000	-0.735824000	0.725792000
H	-2.958368000	-0.523831000	1.799612000
H	-3.371018000	0.117966000	0.197651000
C	-3.600503000	-2.059892000	0.372806000
H	-3.136231000	-2.906223000	0.890377000
H	-3.565096000	-2.256184000	-0.702130000
H	-4.655844000	-2.040780000	0.669321000

$HTeO_3Et$

E = -2.139122

G = -2.092000

N_{imag} = 0

Te	-0.989889000	-0.802000000	1.984126000
O	-1.298418000	-0.501381000	0.236998000
O	-0.030339000	-2.517068000	2.125674000
O	-2.338655000	-0.898470000	3.177185000
C	0.619305000	0.482983000	2.698746000
H	0.117441000	1.448139000	2.812632000
H	0.837427000	0.069990000	3.686832000
C	1.812061000	0.524310000	1.760122000
H	1.532997000	0.872239000	0.762917000
H	2.292983000	-0.452248000	1.667497000

H	2.551306000	1.224438000	2.166630000
H	-0.608197000	-3.115434000	2.628187000

Transition states

HSOEt‡

E = -1.724630
G = -1.681268
N_{imag} = -947.285

S	0.616276000	-0.642266000	-0.599550000
C	0.703089000	-0.027084000	-2.998359000
H	-0.109774000	0.691141000	-2.953717000
H	1.698155000	0.402024000	-2.937355000
C	0.502712000	-1.321507000	-3.496222000
H	0.408131000	-1.975624000	-2.314383000
H	1.352948000	-1.835385000	-3.943806000
H	-0.463870000	-1.555412000	-3.941981000
O	0.392102000	-2.119615000	-1.076256000
H	-0.659450000	-0.156437000	-0.467679000

HSO_2Et‡

E = -1.972063
G = -1.924821
N_{imag} = -897.183

S	-0.672849000	-0.370955000	0.048323000
O	-0.156910000	-0.274184000	1.638310000
O	-0.710051000	-1.878744000	-0.335115000
H	0.812860000	-0.300103000	1.623647000
C	-3.079643000	-0.702662000	0.715538000
H	-2.879495000	-0.325159000	1.713597000
H	-3.474345000	0.023926000	0.011597000
C	-3.146659000	-2.066263000	0.455242000
H	-1.813659000	-2.199479000	-0.026371000
H	-3.699646000	-2.412398000	-0.416738000
H	-3.094500000	-2.765746000	1.288301000

HSO_3Et‡

E = -2.200711
G = -2.152137
N_{imag} = -1230.495

S	-1.028431000	-0.224176000	-0.118556000
O	-1.253766000	0.577280000	-1.555095000
O	-0.918924000	-1.690686000	-0.543324000
H	-0.410439000	1.028644000	-1.737677000
C	-3.260808000	-0.749371000	0.934270000
H	-2.940447000	-0.379600000	1.904201000
H	-3.785786000	-0.031021000	0.311050000
C	-3.225620000	-2.107251000	0.617780000
H	-2.044832000	-2.078885000	-0.019581000
H	-3.875676000	-2.464493000	-0.180340000
H	-3.017858000	-2.816123000	1.418404000
O	0.201174000	0.319519000	0.465484000

HSeOEt‡

E = -1.685686
G = -1.645254
N_{imag} = -899.872

Se	0.662368000	-0.599419000	-0.541261000
C	0.715274000	-0.027252000	-2.991715000

$HSeO_2Et$‡

E = -1.935338
G = -1.890901
N_{imag} = -1000.123

Se	-0.651562000	-0.328128000	-0.010148000
O	-0.093899000	-0.276039000	1.737575000

H	-0.089847000	0.700791000	-2.964128000
H	1.712634000	0.402397000	-2.980134000
C	0.497886000	-1.334060000	-3.485545000
H	0.417664000	-2.003952000	-2.363444000
H	1.338240000	-1.823788000	-3.978202000
H	-0.471575000	-1.536627000	-3.941700000
O	0.411461000	-2.207534000	-1.077912000
H	-0.753786000	-0.110720000	-0.405266000

O	-0.713986000	-1.981119000	-0.410006000
H	0.869877000	-0.378719000	1.712933000
C	-3.054533000	-0.700539000	0.708515000
H	-2.856588000	-0.336473000	1.712635000
H	-3.499774000	0.026868000	0.035255000
C	-3.131611000	-2.075016000	0.436886000
H	-1.874921000	-2.258101000	-0.059397000
H	-3.746911000	-2.398047000	-0.402219000
H	-3.101804000	-2.763904000	1.280440000

HSeO3Et‡

E = -2.123199

G = -2.077982

N_{imag} = -762.759

Se	-0.968524000	-0.215988000	-0.057843000
O	-1.329598000	0.658874000	-1.625784000
O	-0.802895000	-1.826913000	-0.528164000
H	-0.496787000	1.114968000	-1.839754000
C	-3.269870000	-0.756176000	0.952127000
H	-3.004422000	-0.417276000	1.950146000
H	-3.779321000	-0.024610000	0.330200000
C	-3.215662000	-2.123560000	0.594451000
H	-2.111397000	-2.198239000	0.031146000
H	-3.896475000	-2.437906000	-0.198531000
H	-3.112572000	-2.832030000	1.417897000
O	0.426111000	0.442693000	0.470726000

HTeOEt‡

E = -1.661712

G = -1.623607

N_{imag} = -933.114

Te	0.712818000	-0.569694000	-0.436853000
C	0.736970000	-0.022430000	-3.007302000
H	-0.061720000	0.713610000	-2.997665000
H	1.733692000	0.407726000	-3.048101000
C	0.501986000	-1.339540000	-3.492237000
H	0.429275000	-2.035191000	-2.394704000
H	1.330905000	-1.821797000	-4.011726000
H	-0.474195000	-1.524626000	-3.942266000
O	0.425836000	-2.301882000	-1.109474000
H	-0.895248000	-0.046340000	-0.288979000

$HTeO_2Et$‡

E = -1.922111

G = -1.879597

N_{imag} = -1060.447

Te	0.160292000	0.409007000	-0.511593000
C	-0.552347000	2.094834000	-2.395368000
H	-1.032495000	2.777792000	-1.698845000
H	0.437813000	2.406236000	-2.717268000
C	-1.325166000	1.218699000	-3.192860000
H	-1.186281000	0.061742000	-2.511569000
H	-0.930346000	0.952740000	-4.173193000
H	-2.408299000	1.332197000	-3.151733000
O	-0.829237000	-0.739713000	-1.610653000
O	-1.241198000	1.174700000	0.684026000
H	-1.434278000	0.520226000	1.371227000

$HTeO_3Et$‡

E = -2.106398

G = -2.062707

N_{imag} = -545.639

Te	-0.130280000	0.771878000	-0.398351000
C	-0.626244000	2.225258000	-2.588496000
H	-1.235839000	2.982559000	-2.102303000
H	0.417340000	2.495746000	-2.732071000
C	-1.217148000	1.141562000	-3.307162000
H	-1.172767000	0.194558000	-2.554102000
H	-0.632926000	0.786362000	-4.159234000
H	-2.283760000	1.252235000	-3.513411000
O	-0.876286000	-0.666101000	-1.302207000
O	1.817287000	0.451919000	-0.575807000
H	2.119971000	0.107359000	0.281365000
O	-0.400480000	0.671066000	1.387554000

Products

Ethylene

E = -1.139577

G = -1.110555

N_{imag} = 0

C	0.000000000	0.000000000	-0.665590000
C	0.000000000	0.000000000	0.665590000
H	0.000000000	0.925342000	-1.237272000
H	0.000000000	-0.925342000	-1.237272000
H	0.000000000	0.925342000	1.237272000
H	0.000000000	-0.925342000	1.237272000

H_2SO

E = -0.609715

G = -0.611198

N_{imag} = 0

S	0.557597000	-0.576954000	-1.948942000
H	0.722589000	-1.800816000	-2.510450000
O	-0.021518000	-1.062113000	-0.446368000
H	0.753557000	-1.143681000	0.128403000

H_2SO_2

E = -0.846951

G = -0.844209

N_{imag} = 0

S	2.125559000	-2.343165000	0.000000000
O	2.689676000	-1.499353000	-1.317030000
O	2.689676000	-1.499353000	1.317030000
H	3.548487000	-1.875690000	1.565824000
H	3.548487000	-1.875690000	-1.565824000

H_2SO_3

E = -1.109808

G = -1.106208

N_{imag} = 0

S	-0.220152000	0.240723000	-3.318406000
O	-0.037950000	-0.125500000	-4.918571000
O	0.850596000	1.487461000	-3.153358000
O	0.340356000	-0.887178000	-2.569929000
H	0.658871000	-0.804814000	-4.985982000
H	1.710520000	1.103496000	-2.898952000

H_2SeO

E = -0.579597

G = -0.584105

N_{imag} = 0

H_2SeO_2

E = -0.819948

G = -0.819863

N_{imag} = 0

Se	0.563562000	-0.509742000	-2.004089000
H	0.742552000	-1.863659000	-2.588306000
O	-0.044442000	-1.059943000	-0.364896000
H	0.750552000	-1.150220000	0.179934000

Se	1.628044000	-2.630243000	-1.699057000
O	1.879773000	-1.909896000	-3.349186000
O	2.605692000	-1.528312000	-0.633543000
H	3.497642000	-1.904701000	-0.594150000
H	2.661446000	-2.344327000	-3.722060000

H_2SeO_3

E = -1.056320
G = -1.056072
N_{imag} = 0

Se	-0.289583000	0.259553000	-3.300209000
O	-0.049827000	-0.160440000	-5.053032000
O	0.921729000	1.603552000	-3.122812000
O	0.373698000	-0.985471000	-2.497095000
H	0.606348000	-0.880081000	-5.061004000
H	1.739877000	1.177075000	-2.811046000

H_2TeO

E = -0.558928
G = -0.565671
N_{imag} = 0

Te	0.561370000	-0.422649000	-2.063266000
H	0.768970000	-1.956052000	-2.714457000
O	-0.057156000	-1.052162000	-0.269922000
H	0.739040000	-1.152701000	0.270289000

H_2TeO_2

E = -0.811349
G = -0.813051
N_{imag} = 0

Te	1.965419000	-2.475689000	0.000000000
O	2.733594000	-1.452201000	-1.519611000
O	2.733594000	-1.452201000	1.519611000
H	3.584639000	-1.856581000	1.740391000
H	3.584639000	-1.856581000	-1.740391000

H_2TeO_3

E = -1.050276
G = -1.051973
N_{imag} = 0

Te	-0.384707000	0.283729000	-3.275630000
O	-0.059105000	-0.202453000	-5.157640000
O	0.983883000	1.690125000	-3.087444000
O	0.375973000	-1.091928000	-2.399254000
H	0.590859000	-0.925974000	-5.159005000
H	1.795337000	1.260688000	-2.766223000

Table S12: Cartesian coordinates (Å), energies (a.u.) and imaginary frequencies (cm^{-1}) of the optimized structures of the minimal model. Level of theory: ZORA-B3LYP/TZ2P-ae.

Reactants

HSOEt

E = -2.109460
G = -2.059186
N_{imag} = 0

S	0.892074000	-0.182223000	-0.627575000
C	0.758794000	-0.024622000	-2.453091000
H	-0.087716000	0.630844000	-2.656913000
H	1.680141000	0.469071000	-2.773006000
C	0.582114000	-1.393371000	-3.094828000
H	-0.313372000	-1.883398000	-2.714137000

HSO_2Et

E = -2.441496
G = -2.387135
N_{imag} = 0

S	-1.105560000	-0.496789000	0.176800000
O	-1.501894000	-0.454506000	-1.451749000
O	-0.678856000	0.840901000	0.604518000
H	-0.685783000	-0.579858000	-1.955215000
C	-2.855171000	-0.650670000	0.665218000
H	-2.829032000	-0.493225000	1.744108000

H	1.438735000	-2.039286000	-2.896562000
H	0.479137000	-1.291289000	-4.175465000
O	-0.436489000	-0.587316000	-0.088950000
H	1.005283000	1.187785000	-0.490967000

H	-3.356301000	0.200531000	0.203009000
C	-3.480074000	-1.988012000	0.294427000
H	-2.916845000	-2.822716000	0.714666000
H	-3.527297000	-2.113996000	-0.785661000
H	-4.495306000	-2.043420000	0.688442000

HSO_3Et

E = -2.766837

G = -2.707426

N_{imag} = 0

S	-0.820088000	-0.758031000	2.003072000
O	-1.076394000	-0.421018000	0.635949000
O	-0.062267000	-2.189431000	1.997531000
O	-1.885484000	-0.849362000	2.966304000
C	0.457591000	0.325462000	2.643869000
H	-0.048322000	1.287853000	2.731126000
H	0.677261000	-0.036384000	3.647479000
C	1.693625000	0.406914000	1.758227000
H	1.433476000	0.748520000	0.758390000
H	2.186999000	-0.560296000	1.677979000
H	2.399131000	1.115197000	2.192655000
H	-0.457506000	-2.733927000	2.694965000

HSeOEt

E = -2.049620

G = -2.003623

N_{imag} = 0

Se	0.951197000	-0.193537000	-0.523928000
C	0.793073000	-0.032723000	-2.511719000
H	-0.057365000	0.626155000	-2.672398000
H	1.707206000	0.455917000	-2.851855000
C	0.585312000	-1.405911000	-3.126353000
H	-0.290038000	-1.892111000	-2.696017000
H	1.450744000	-2.052365000	-2.971609000
H	0.425305000	-1.316160000	-4.202079000
O	-0.569369000	-0.620676000	-0.022191000
H	1.002637000	1.317610000	-0.393345000

$HSeO_2Et$

E = -2.381599

G = -2.331570

N_{imag} = 0

Se	-0.983255000	-0.544303000	0.209710000
O	-1.464685000	-0.514938000	-1.568507000
O	-0.594223000	0.985337000	0.627161000
H	-0.652059000	-0.438763000	-2.087392000
C	-2.892373000	-0.686988000	0.711561000
H	-2.883512000	-0.518120000	1.788197000
H	-3.342271000	0.173869000	0.220276000
C	-3.525666000	-2.011899000	0.320757000
H	-3.019327000	-2.857827000	0.788745000
H	-3.507747000	-2.153597000	-0.758540000
H	-4.567003000	-2.034533000	0.646597000

$HSeO_3Et$

E = -2.640756

G = -2.588013

N_{imag} = 0

Se	-0.880514000	-0.765551000	2.004314000
O	-1.175655000	-0.443725000	0.454718000
O	0.005369000	-2.325078000	2.077509000
O	-2.086735000	-0.889964000	3.073323000
C	0.535940000	0.420575000	2.681618000
H	0.024791000	1.378703000	2.761475000
H	0.739320000	0.028502000	3.675639000
C	1.740331000	0.449143000	1.759690000
H	1.462017000	0.771141000	0.758078000
H	2.215823000	-0.527812000	1.695400000
H	2.468631000	1.157214000	2.158181000
H	-0.551296000	-2.917649000	2.607599000

HTeOEt

E = -2.016045

G = -1.973216

N_{imag} = 0

Te	1.025109000	-0.190380000	-0.390813000
C	0.828692000	-0.049984000	-2.564938000
H	-0.012416000	0.624287000	-2.712931000
H	1.738626000	0.421121000	-2.937516000
C	0.577754000	-1.423195000	-3.171047000
H	-0.299625000	-1.891777000	-2.725132000
H	1.430042000	-2.090757000	-3.032729000
H	0.399472000	-1.335702000	-4.244389000
O	-0.664816000	-0.692309000	0.089194000
H	0.975864000	1.514895000	-0.281193000

$HTeO_2Et$

E = -2.359351

G = -2.312190

N_{imag} = 0

Te	-0.779691000	-0.651411000	0.313726000
O	-1.035407000	-0.885924000	-1.634365000
O	-0.485137000	1.129408000	0.453424000
H	-1.091976000	0.001213000	-2.022020000
C	-2.905106000	-0.724715000	0.753701000
H	-2.976060000	-0.548609000	1.828134000
H	-3.290541000	0.154429000	0.237744000
C	-3.580102000	-2.020264000	0.323419000
H	-3.177968000	-2.884830000	0.854391000
H	-3.460246000	-2.192994000	-0.745793000
H	-4.649885000	-1.978062000	0.536204000

$HTeO_3Et$

E = -2.606878

G = -2.558528

N_{imag} = 0

Te	-0.986134000	-0.779768000	2.002378000
O	-1.295007000	-0.478255000	0.258491000
O	0.011787000	-2.451124000	2.141292000
O	-2.339053000	-0.921851000	3.180849000
C	0.604487000	0.502723000	2.708469000
H	0.124754000	1.476326000	2.794487000
H	0.818585000	0.114046000	3.702042000
C	1.796637000	0.493607000	1.764836000
H	1.516757000	0.816518000	0.763622000
H	2.247575000	-0.494926000	1.698261000
H	2.551081000	1.184145000	2.146144000
H	-0.553446000	-3.125942000	2.546676000

Transition states

$HSOEt^{‡}$

E = -2.062665
G = -2.017813
N_{imag} = -1162.901

S	0.615312000	-0.636331000	-0.610176000
C	0.701871000	-0.033864000	-2.983579000
H	-0.106546000	0.682333000	-2.944143000
H	1.691968000	0.395513000	-2.932537000
C	0.501176000	-1.325826000	-3.487709000
H	0.408752000	-1.990927000	-2.332604000
H	1.348102000	-1.820278000	-3.953045000
H	-0.459427000	-1.543416000	-3.944393000
O	0.393894000	-2.113893000	-1.061639000
H	-0.654901000	-0.153510000	-0.479575000

$HSO_2Et^{‡}$

E = -2.383637
G = -2.334720
N_{imag} = -1202.516

S	-0.695082000	-0.375956000	0.035355000
O	-0.205627000	-0.284358000	1.625597000
O	-0.696662000	-1.871039000	-0.346712000
H	0.761334000	-0.300133000	1.652288000
C	-3.040632000	-0.699005000	0.708522000
H	-2.828523000	-0.324733000	1.700608000
H	-3.460272000	0.022696000	0.021633000
C	-3.123134000	-2.064195000	0.453676000
H	-1.827185000	-2.226985000	-0.037737000
H	-3.716004000	-2.398873000	-0.390849000
H	-3.083111000	-2.749219000	1.293919000

$HSO_3Et^{‡}$

E = -2.681772
G = -2.631291
N_{imag} = -1365.669

S	-1.070438000	-0.140587000	-0.068351000
O	-1.253578000	0.593595000	-1.529430000
O	-0.810172000	-1.590517000	-0.431020000
H	-0.440521000	1.097802000	-1.699843000
C	-3.317843000	-0.821509000	0.847772000
H	-3.097985000	-0.396077000	1.817098000
H	-3.846733000	-0.172051000	0.163979000
C	-3.152229000	-2.178529000	0.587777000
H	-1.947983000	-2.086513000	0.034762000
H	-3.723127000	-2.609352000	-0.228652000
H	-2.959867000	-2.835436000	1.429768000
O	0.059177000	0.523074000	0.572741000

$HSeOEt^{‡}$

E = -2.019335
G = -1.977450
N_{imag} = -1070.172

$HSeO_2Et^{‡}$

E = -2.342060
G = -2.295986
N_{imag} = -1220.814

Se	0.660904000	-0.592349000	-0.544440000
C	0.714699000	-0.032850000	-2.983813000
H	-0.087295000	0.691325000	-2.958475000
H	1.706597000	0.396649000	-2.976702000
C	0.498039000	-1.337311000	-3.480773000
H	0.416907000	-2.014190000	-2.377527000
H	1.335736000	-1.812067000	-3.983464000
H	-0.465318000	-1.527651000	-3.945249000
O	0.409191000	-2.202285000	-1.064403000
H	-0.749261000	-0.109471000	-0.414554000

Se	-0.828914000	-0.356612000	0.078414000
O	-0.155751000	-0.287134000	1.772834000
O	-0.911532000	-2.009197000	-0.277893000
H	0.800411000	-0.418012000	1.714850000
C	-3.126810000	-0.690532000	1.015307000
H	-2.824741000	-0.308416000	1.981235000
H	-3.641203000	0.020343000	0.382944000
C	-3.243614000	-2.070445000	0.788959000
H	-2.066027000	-2.294930000	0.183833000
H	-3.958708000	-2.398337000	0.041220000
H	-3.149810000	-2.726527000	1.648597000

$HSeO_3Et^{‡}$

E = -2.595768

G = -2.548874

N_{imag} = -855.202

Se	-0.958626000	-0.210749000	-0.043677000
O	-1.346500000	0.652368000	-1.589866000
O	-0.791561000	-1.808969000	-0.517115000
H	-0.523218000	1.086991000	-1.866022000
C	-3.267755000	-0.758197000	0.945132000
H	-3.018118000	-0.415237000	1.940139000
H	-3.756776000	-0.030865000	0.310497000
C	-3.212926000	-2.123683000	0.596121000
H	-2.109981000	-2.204870000	0.044035000
H	-3.887640000	-2.435294000	-0.197490000
H	-3.128875000	-2.822928000	1.423845000
O	0.440676000	0.455335000	0.451002000

$HTeOEt^{‡}$

E = -1.989151

G = -1.949652

N_{imag} = -1097.587

Te	0.715640000	-0.562291000	-0.432798000
C	0.734585000	-0.027511000	-3.007383000
H	-0.062797000	0.702502000	-2.995815000
H	1.725331000	0.403043000	-3.051146000
C	0.501367000	-1.344028000	-3.489180000
H	0.428471000	-2.042198000	-2.407659000
H	1.327844000	-1.813453000	-4.015601000
H	-0.469073000	-1.520678000	-3.944941000
O	0.424826000	-2.293664000	-1.095009000
H	-0.885995000	-0.041922000	-0.289868000

$HTeO_2Et^{‡}$

E = -2.323067

G = -2.279052

N_{imag} = -1264.772

Te	0.171018000	0.388960000	-0.518112000
C	-0.543797000	2.080451000	-2.381263000
H	-1.016669000	2.747002000	-1.671139000
H	0.435216000	2.400269000	-2.711562000
C	-1.324355000	1.213791000	-3.180332000
H	-1.199132000	0.059939000	-2.530523000
H	-0.940749000	0.977304000	-4.168097000
H	-2.401136000	1.345933000	-3.134508000
O	-0.831556000	-0.751309000	-1.598797000
O	-1.222635000	1.185105000	0.645783000
H	-1.467705000	0.560956000	1.340749000

$HTeO_3Et^‡$

E = -2.572483

G = -2.527343

N_{imag} = -628.462

Te	-0.139912000	0.769383000	-0.382258000
C	-0.633816000	2.224919000	-2.587440000
H	-1.244212000	2.976230000	-2.104874000
H	0.408833000	2.488227000	-2.711751000
C	-1.215135000	1.144835000	-3.311127000
H	-1.170457000	0.197885000	-2.566624000
H	-0.624794000	0.801330000	-4.158461000
H	-2.275183000	1.258866000	-3.526039000
O	-0.855440000	-0.661142000	-1.301360000
O	1.794988000	0.488686000	-0.567045000
H	2.131025000	0.094128000	0.252536000
O	-0.396996000	0.631154000	1.400243000

Products

Ethylene

E = -1.325471

G = -1.295669

N_{imag} = 0

C	0.000000000	0.000000000	-0.662251000
C	0.000000000	0.000000000	0.662251000
H	0.000000000	0.921454000	-1.231091000
H	0.000000000	-0.921454000	-1.231091000
H	0.000000000	0.921454000	1.231091000
H	0.000000000	-0.921454000	1.231091000

H_2SO

E = -0.766465

G = -0.767606

N_{imag} = 0

S	0.554908000	-0.577769000	-1.953472000
H	0.721098000	-1.798581000	-2.508583000
O	-0.012795000	-1.062182000	-0.451140000
H	0.749014000	-1.145033000	0.135839000

H_2SO_2

E = -1.075875

G = -1.072689

N_{imag} = 0

S	2.115833000	-2.342707000	0.000000000
O	2.693895000	-1.504207000	-1.304331000
O	2.693895000	-1.504207000	1.304331000
H	3.549131000	-1.871065000	1.566616000
H	3.549131000	-1.871065000	-1.566616000

H_2SO_3

E = -1.408040

G = -1.403805

N_{imag} = 0

S	-0.223206000	0.242794000	-3.318707000
O	-0.030584000	-0.120848000	-4.902235000
O	0.846362000	1.470802000	-3.160568000
O	0.342894000	-0.878229000	-2.579277000
H	0.657696000	-0.804113000	-4.985696000
H	1.709079000	1.103782000	-2.898716000

H_2SeO

E = -0.732397

G = -0.736529

N_{imag} = 0

H_2SeO_2

E = -1.044768

G = -1.044244

N_{imag} = 0

Se	0.560621000	-0.510247000	-2.008774000
H	0.740937000	-1.860968000	-2.584426000
O	-0.035426000	-1.059934000	-0.371384000
H	0.746092000	-1.152415000	0.187227000

Se	2.045825000	-2.406492000	0.000000000
O	2.711784000	-1.479148000	-1.402296000
O	2.711784000	-1.479148000	1.402296000
H	3.566246000	-1.864232000	1.635673000
H	3.566246000	-1.864232000	-1.635673000

H_2SeO_3

E = -1.347944

G = -1.347017

N_{imag} = 0

Se	-0.297092000	0.262863000	-3.299571000
O	-0.041484000	-0.153480000	-5.030869000
O	0.914364000	1.581813000	-3.132078000
O	0.374582000	-0.976457000	-2.505672000
H	0.613360000	-0.871217000	-5.055205000
H	1.738512000	1.170666000	-2.821804000

H_2TeO

E = -0.706699

G = -0.713044

N_{imag} = 0

Te	0.557230000	-0.422498000	-2.068003000
H	0.767736000	-1.953885000	-2.710449000
O	-0.046353000	-1.050897000	-0.278178000
H	0.733611000	-1.156285000	0.279274000

H_2TeO_2

E = -1.031938

G = -1.033225

N_{imag} = 0

Te	1.953687000	-2.473526000	0.000000000
O	2.737345000	-1.460319000	-1.503462000
O	2.737345000	-1.460319000	1.503462000
H	3.586754000	-1.849544000	1.744009000
H	3.586754000	-1.849544000	-1.744009000

H_2TeO_3

E = -1.337807

G = -1.338868

N_{imag} = 0

Te	-0.391864000	0.286298000	-3.274119000
O	-0.051326000	-0.197707000	-5.135135000
O	0.976771000	1.668108000	-3.094347000
O	0.373528000	-1.084305000	-2.405253000
H	0.597294000	-0.918950000	-5.160666000
H	1.797837000	1.260742000	-2.775676000

Table S13: Cartesian coordinates (Å), energies (a.u.) and imaginary frequencies (cm^{-1}) of the optimized structures of the minimal model. Level of theory: ZORA-BLYP-D3(BJ)/TZ2P.

Reactants

HSOEt

E = -1.755476

G = -1.707843

N_{imag} = 0

S	0.899361000	-0.183046000	-0.610734000
C	0.766651000	-0.017647000	-2.465562000
H	-0.082098000	0.645042000	-2.664084000
H	1.696662000	0.473543000	-2.782270000
C	0.580074000	-1.396389000	-3.099389000
H	-0.320522000	-1.880008000	-2.707019000

HSO_2Et

E = -2.011166

G = -1.959875

N_{imag} = 0

S	-1.088188000	-0.498441000	0.185813000
O	-1.511945000	-0.449704000	-1.481575000
O	-0.649949000	0.853210000	0.625107000
H	-0.680746000	-0.582722000	-1.977654000
C	-2.867134000	-0.647274000	0.674067000
H	-2.842586000	-0.485759000	1.757587000

H	1.438869000	-2.047560000	-2.897505000
H	0.471812000	-1.302436000	-4.185803000
O	-0.443277000	-0.613002000	-0.074085000
H	0.991171000	1.207700000	-0.485043000

H	-3.361588000	0.207168000	0.199162000
C	-3.485121000	-1.994215000	0.296779000
H	-2.915705000	-2.828890000	0.723003000
H	-3.521169000	-2.118362000	-0.789162000
H	-4.507988000	-2.056771000	0.685436000

HSO_3Et

E = -2.255623

G = -2.199416

N_{imag} = 0

S	-0.834180000	-0.755369000	1.998102000
O	-1.081423000	-0.415631000	0.610168000
O	-0.047353000	-2.215389000	1.991846000
O	-1.914994000	-0.852867000	2.972529000
C	0.461929000	0.338211000	2.653227000
H	-0.045758000	1.305274000	2.741991000
H	0.679321000	-0.035865000	3.658295000
C	1.698793000	0.409919000	1.756187000
H	1.432272000	0.755931000	0.753851000
H	2.182391000	-0.567653000	1.672754000
H	2.416567000	1.115189000	2.189415000
H	-0.449543000	-2.746253000	2.709181000

HSeOEt

E = -1.703921

G = -1.660639

N_{imag} = 0

Se	0.966061000	-0.185644000	-0.504712000
C	0.810383000	-0.021098000	-2.533737000
H	-0.032565000	0.655759000	-2.693028000
H	1.741943000	0.450770000	-2.866847000
C	0.576659000	-1.405878000	-3.127020000
H	-0.308230000	-1.870867000	-2.679013000
H	1.436712000	-2.066411000	-2.962965000
H	0.410978000	-1.332537000	-4.208887000
O	-0.555719000	-0.689442000	-0.009474000
H	0.952480000	1.351546000	-0.385811000

$HSeO_2Et$

E = -1.961296

G = -1.914401

N_{imag} = 0

Se	-0.963044000	-0.545241000	0.217966000
O	-1.478752000	-0.507111000	-1.600901000
O	-0.552378000	0.997665000	0.657515000
H	-0.649284000	-0.447313000	-2.114458000
C	-2.910740000	-0.681631000	0.721241000
H	-2.902089000	-0.507853000	1.802071000
H	-3.352761000	0.180995000	0.215091000
C	-3.530088000	-2.018233000	0.322865000
H	-3.014729000	-2.862089000	0.797601000
H	-3.498787000	-2.157802000	-0.761711000
H	-4.579469000	-2.053148000	0.641285000

$HSeO_3Et$

E = -2.145492

G = -2.096144

N_{imag} = 0

Se	-0.899499000	-0.763889000	2.004450000
O	-1.180391000	-0.437765000	0.426793000
O	0.038497000	-2.352022000	2.096720000
O	-2.132738000	-0.892707000	3.080073000
C	0.550126000	0.444416000	2.698063000
H	0.032064000	1.404620000	2.773867000
H	0.748651000	0.037948000	3.692509000
C	1.745492000	0.452656000	1.756963000
H	1.454099000	0.770256000	0.751854000
H	2.211291000	-0.534863000	1.696306000
H	2.489133000	1.161735000	2.141864000
H	-0.558703000	-2.954888000	2.588083000

HTeOEt

E = -1.681111
G = -2.454722
$N_{imag} = 0$

Te	1.049118000	-0.172126000	-0.374687000
C	0.863411000	-0.033312000	-2.590862000
H	0.043467000	0.672991000	-2.743921000
H	1.798964000	0.399453000	-2.961157000
C	0.559841000	-1.417502000	-3.161155000
H	-0.325017000	-1.849057000	-2.680200000
H	1.399740000	-2.108670000	-3.019234000
H	0.361132000	-1.351627000	-4.238415000
O	-0.629838000	-0.804559000	0.075949000
H	0.877885000	1.550607000	-0.277813000

HTeO$_2$Et

E = -2.960916
G = -1.640792
$N_{imag} = 0$

Te	1.049118000	-0.172126000	-0.374687000
C	0.863411000	-0.033312000	-2.590862000
H	0.043467000	0.672991000	-2.743921000
H	1.798964000	0.399453000	-2.961157000
C	0.559841000	-1.417502000	-3.161155000
H	-0.325017000	-1.849057000	-2.680200000
H	1.399740000	-2.108670000	-3.019234000
H	0.361132000	-1.351627000	-4.238415000
O	-0.629838000	-0.804559000	0.075949000
H	0.877885000	1.550607000	-0.277813000
Te	1.049118000	-0.172126000	-0.374687000

HTeO$_3$Et

E = -2.125367
G = -2.080207
$N_{imag} = 0$

Te	-1.008873000	-0.775302000	2.004401000
O	-1.294275000	-0.471351000	0.229847000
O	0.053606000	-2.457924000	2.174325000
O	-2.397374000	-0.929189000	3.179892000
C	0.619646000	0.527332000	2.728635000
H	0.136834000	1.504871000	2.812165000
H	0.828510000	0.120885000	3.721438000
C	1.797339000	0.491015000	1.760970000
H	1.501629000	0.810497000	0.757272000
H	2.232885000	-0.510253000	1.696677000
H	2.572469000	1.178106000	2.124483000
H	-0.544374000	-3.153189000	2.517442000

Transition states

HSOEt‡

E = -1.718466
G = -1.676352
N_{imag} = -1019.822

S	0.617815000	-0.647549000	-0.557046000
C	0.705000000	-0.015959000	-3.022412000
H	-0.113504000	0.694665000	-2.961290000
H	1.700006000	0.411484000	-2.951218000
C	0.504335000	-1.324575000	-3.496809000
H	0.409530000	-1.985020000	-2.339082000
H	1.355276000	-1.832581000	-3.953554000
H	-0.461980000	-1.555928000	-3.948380000
O	0.387033000	-2.135749000	-1.051012000
H	-0.663312000	-0.148989000	-0.448596000

HSO_2Et‡

E = -1.963209
G = -1.917658
N_{imag} = -1235.674

S	-0.655633000	-0.365401000	0.015852000
O	-0.174805000	-0.262979000	1.645464000
O	-0.684234000	-1.886574000	-0.361293000
H	0.801544000	-0.311226000	1.664420000
C	-3.081058000	-0.695007000	0.714230000
H	-2.846877000	-0.313522000	1.703791000
H	-3.487838000	0.028252000	0.013500000
C	-3.140738000	-2.066401000	0.456879000
H	-1.832610000	-2.229005000	-0.041401000
H	-3.726530000	-2.413611000	-0.394752000
H	-3.086120000	-2.756327000	1.299609000

HSO_3Et‡

E = -2.183988
G = -2.137128
N_{imag} = -1209.470

S	-1.028931000	-0.142158000	-0.057016000
O	-1.260092000	0.593034000	-1.560642000
O	-0.789474000	-1.623860000	-0.421374000
H	-0.436954000	1.096600000	-1.740974000
C	-3.349847000	-0.812714000	0.870660000
H	-3.123925000	-0.392248000	1.846414000
H	-3.852679000	-0.147909000	0.173994000
C	-3.172020000	-2.172629000	0.593373000
H	-1.968371000	-2.096788000	0.052870000
H	-3.735666000	-2.597289000	-0.239005000
H	-2.989923000	-2.843059000	1.434446000
O	0.146582000	0.522920000	0.543854000

HSeOEt‡

E = -1.682011
G = -1.642591
N_{imag} = -817.837

Se	0.663163000	-0.604066000	-0.491305000
C	0.718482000	-0.016149000	-3.020089000

$HSeO_2Et$‡

E = -1.929910
G = -1.886944
N_{imag} = -1097.946

Se	-0.608101000	-0.325695000	-0.050087000
O	-0.098923000	-0.256019000	1.742379000

H	-0.092551000	0.704160000	-2.973288000
H	1.716606000	0.410393000	-2.994005000
C	0.500437000	-1.337804000	-3.487981000
H	0.418063000	-2.009064000	-2.392197000
H	1.341392000	-1.823545000	-3.987435000
H	-0.468450000	-1.537759000	-3.950671000
O	0.404009000	-2.226202000	-1.051786000
H	-0.760951000	-0.100164000	-0.380643000

O	-0.689492000	-2.003395000	-0.425253000
H	0.867339000	-0.400487000	1.759196000
C	-3.074945000	-0.691533000	0.704283000
H	-2.835612000	-0.316121000	1.695277000
H	-3.517326000	0.029904000	0.023080000
C	-3.133479000	-2.072591000	0.440951000
H	-1.900730000	-2.278075000	-0.060303000
H	-3.766693000	-2.405430000	-0.382815000
H	-3.097738000	-2.749758000	1.295792000

$HSeO_3Et^{‡}$

E = -2.112501
G = -2.068635
N_{imag} = -577.266

Se	-0.908254000	-0.215075000	-0.010989000
O	-1.366554000	0.664225000	-1.589813000
O	-0.732354000	-1.844934000	-0.484208000
H	-0.537057000	1.096471000	-1.885600000
C	-3.300633000	-0.751693000	0.942726000
H	-3.071213000	-0.410527000	1.948630000
H	-3.753657000	-0.015636000	0.283324000
C	-3.224401000	-2.124924000	0.583709000
H	-2.122604000	-2.228960000	0.067405000
H	-3.883771000	-2.432710000	-0.232438000
H	-3.175811000	-2.829417000	1.417739000
O	0.515011000	0.477080000	0.456114000

$HTeOEt^{‡}$

E = -1.661737
G = -1.624512
N_{imag} = -815.819

Te	0.717710000	-0.572866000	-0.381899000
C	0.737525000	-0.012667000	-3.041131000
H	-0.068833000	0.713945000	-3.006586000
H	1.735127000	0.415764000	-3.066361000
C	0.503804000	-1.344977000	-3.496328000
H	0.431736000	-2.035706000	-2.422873000
H	1.333190000	-1.824238000	-4.021537000
H	-0.472681000	-1.531122000	-3.949565000
O	0.421776000	-2.317106000	-1.081510000
H	-0.899154000	-0.031228000	-0.261610000

$HTeO_2Et^{‡}$

E = -1.920476
G = -1.879261
N_{imag} = -1059.052

Te	0.191514000	0.355801000	-0.482623000
C	-0.549448000	2.114326000	-2.414485000
H	-1.017550000	2.766397000	-1.681284000
H	0.444120000	2.416177000	-2.733698000
C	-1.326348000	1.221387000	-3.194427000
H	-1.194614000	0.073849000	-2.541300000
H	-0.944411000	0.966925000	-4.184682000
H	-2.410363000	1.338827000	-3.142071000
O	-0.833127000	-0.772941000	-1.605020000
O	-1.226872000	1.187647000	0.692416000
H	-1.474402000	0.540006000	1.379373000

$HTeO_3Et^{‡}$

E = -2.101359
G = -2.058659

N_{imag} = -375.447

Te	-0.181115000	0.756717000	-0.336826000
C	-0.616730000	2.233347000	-2.598377000
H	-1.222118000	3.006225000	-2.132497000
H	0.442620000	2.460066000	-2.695278000
C	-1.213832000	1.141786000	-3.317313000
H	-1.210427000	0.202878000	-2.587080000
H	-0.613063000	0.782245000	-4.158799000
H	-2.270106000	1.285673000	-3.561538000
O	-0.913941000	-0.703154000	-1.255487000
O	1.795475000	0.527137000	-0.606126000
H	2.157163000	0.118420000	0.207532000
O	-0.375026000	0.603160000	1.477588000

Products

Ethylene

E = -1.133714

G = -1.105060

N_{imag} = 0

C	0.000000000	0.000000000	-0.666527000
C	0.000000000	0.000000000	0.666527000
H	0.000000000	0.925831000	-1.238838000
H	0.000000000	-0.925831000	-1.238838000
H	0.000000000	0.925831000	1.238838000
H	0.000000000	-0.925831000	1.238838000

H_2SO

E = -0.599814

G = -0.601947

N_{imag} = 0

S	0.559060000	-0.571282000	-1.964012000
H	0.723840000	-1.804661000	-2.520433000
O	-0.025283000	-1.063016000	-0.436707000
H	0.754607000	-1.144606000	0.143795000

H_2SO_2

E = -0.833417

G = -0.831593

N_{imag} = 0

S	2.109442000	-2.351102000	0.000000000
O	2.689630000	-1.497116000	-1.332842000
O	2.689630000	-1.497116000	1.332842000
H	3.556592000	-1.873958000	1.581267000
H	3.556592000	-1.873958000	-1.581267000

H_2SO_3

E = -1.089766

G = -1.087389

N_{imag} = 0

S	-0.231948000	0.239176000	-3.311030000
O	-0.042370000	-0.130100000	-4.934879000
O	0.857163000	1.502775000	-3.147983000
O	0.338763000	-0.896514000	-2.560483000
H	0.657271000	-0.817846000	-5.003394000
H	1.723362000	1.116697000	-2.887429000

H_2SeO

E = -0.571450

G = -0.576561

N_{imag} = 0

Se	0.564824000	-0.503414000	-2.019380000
H	0.744456000	-1.868648000	-2.600755000

H_2SeO_2

E = -0.809411

G = -0.810176

N_{imag} = 0

Se	2.040298000	-2.414313000	0.000000000
O	2.707444000	-1.471978000	-1.436560000

O	-0.048601000	-1.060225000	-0.353869000
H	0.751545000	-1.151277000	0.196647000

O	2.707444000	-1.471978000	1.436560000
H	3.573350000	-1.867491000	1.653788000
H	3.573350000	-1.867491000	-1.653788000

H_2SeO_3

E = -1.040965

G = -1.041821

N_{imag} = 0

Se	-0.301430000	0.259182000	-3.293900000
O	-0.056477000	-0.168161000	-5.071996000
O	0.929091000	1.621585000	-3.113375000
O	0.369321000	-0.999222000	-2.482319000
H	0.605934000	-0.892971000	-5.082834000
H	1.755803000	1.193775000	-2.800774000

H_2TeO

E = -0.553564

G = -0.560818

N_{imag} = 0

Te	0.562124000	-0.415791000	-2.077393000
H	0.770871000	-1.961765000	-2.727406000
O	-0.060437000	-1.052275000	-0.260095000
H	0.739665000	-1.153733000	0.287538000

H_2TeO_2

E = -0.804012

G = -0.806478

N_{imag} = 0

Te	1.949682000	-2.483280000	0.000000000
O	2.733480000	-1.450627000	-1.535781000
O	2.733480000	-1.450627000	1.535781000
H	3.592621000	-1.854359000	1.759539000
H	3.592621000	-1.854359000	-1.759539000

H_2TeO_3

E = -1.039177

G = -1.041861

N_{imag} = 0

Te	-0.398614000	0.285134000	-3.269859000
O	-0.065436000	-0.209097000	-5.172457000
O	0.989161000	1.704589000	-3.079246000
O	0.371638000	-1.105509000	-2.384635000
H	0.594844000	-0.934098000	-5.177209000
H	1.810647000	1.273168000	-2.761791000

Table 14: Cartesian coordinates (Å), energies (a.u.) and imaginary frequencies (cm^{-1}) of the optimized structures of the amino acid model. Level of theory: ZORA-OPBE/TZ2P.

Cysteine (Cys)
OS 0

Diastereoisomer RR

E = -3.114592

G = -3.039314

N_{imag} = 0

C	1.361043000	-3.364659000	7.422847000
C	2.672631000	-3.535233000	6.662354000
N	1.449242000	-2.899622000	8.803205000
H	2.283307000	-3.256833000	9.258633000
H	1.481511000	-1.888393000	8.857489000
H	0.779673000	-2.616275000	6.863802000
C	0.455888000	-4.621294000	7.361200000
O	0.372289000	-5.330911000	6.389202000

Diastereoisomer RS

E = -3.120126

G = -3.043733

N_{imag} = 0

C	1.357074000	-3.345061000	7.418504000
C	2.672048000	-3.509194000	6.661331000
N	1.441363000	-2.952876000	8.820300000
H	2.306359000	-3.297014000	9.231826000
H	1.412932000	-1.947029000	8.933901000
H	0.786364000	-2.572215000	6.881039000
C	0.454460000	-4.600307000	7.298716000
O	0.353074000	-5.250549000	6.284982000

O	-0.252299000	-4.793523000	8.477416000
H	0.085963000	-4.063042000	9.060775000
H	2.492137000	-3.838534000	5.628059000
H	3.236827000	-2.595746000	6.662688000
S	3.843303000	-4.725926000	7.426404000
H	3.160331000	-5.795679000	6.880186000
O	5.151415000	-4.598844000	6.734184000

O	-0.217955000	-4.838181000	8.419349000
H	0.151369000	-4.132112000	9.027624000
H	2.479793000	-3.880778000	5.647127000
H	3.204000000	-2.553705000	6.587626000
S	3.893320000	-4.671838000	7.347033000
H	2.996842000	-5.719821000	7.247909000
O	4.107115000	-4.389949000	8.798749000

OS +2

Diastereoisomer RR

E = -3.382538

G = -3.302564

N_{imag} = 0

C	1.284085000	-3.461861000	7.278048000
C	2.361815000	-4.044745000	6.367650000
N	1.710563000	-2.577001000	8.356484000
H	2.661455000	-2.786197000	8.640373000
H	1.678871000	-1.604646000	8.075034000
H	0.622766000	-2.884523000	6.615756000
C	0.340850000	-4.554349000	7.849181000
O	-0.054447000	-5.491828000	7.200895000
O	-0.014390000	-4.302254000	9.107679000
H	0.507755000	-3.474103000	9.305185000
H	1.883948000	-4.578119000	5.539939000
H	3.018505000	-3.271199000	5.954040000
S	3.471535000	-5.323948000	7.040223000
O	4.269929000	-4.221882000	8.033407000
H	4.778177000	-4.765904000	8.652355000
O	4.351808000	-5.683257000	5.924815000

Diastereoisomer RS

E = -3.381145

G = -3.299501

N_{imag} = 0

C	1.266456000	-3.477410000	7.343104000
C	2.359715000	-4.110934000	6.484443000
N	1.634429000	-2.786645000	8.566758000
H	2.332764000	-3.318146000	9.075955000
H	2.005774000	-1.862323000	8.382984000
H	0.787197000	-2.734887000	6.685835000
C	0.102673000	-4.459614000	7.627868000
O	-0.248331000	-5.314808000	6.852711000
O	-0.517399000	-4.189446000	8.775273000
H	0.043346000	-3.466254000	9.159406000
H	1.879369000	-4.736944000	5.723996000
H	2.983217000	-3.364032000	5.985841000
S	3.504038000	-5.247323000	7.346262000
O	4.684348000	-4.070061000	7.509891000
H	5.496166000	-4.556436000	7.714370000
O	2.931148000	-5.536321000	8.670754000

OS +4

Diastereoisomer R

E = -3.648052

G = -3.563229

N_{imag} = 0

C	1.311342000	-3.486139000	7.351891000
C	2.384362000	-4.173714000	6.500364000
N	1.674177000	-2.834306000	8.596587000
H	2.317316000	-3.401645000	9.138645000
H	2.102425000	-1.929042000	8.445133000
H	0.889185000	-2.707744000	6.698338000
C	0.092374000	-4.417293000	7.587281000
O	-0.274184000	-5.240353000	6.786853000
O	-0.545123000	-4.140799000	8.723343000
H	0.026877000	-3.444001000	9.134845000
H	1.886889000	-4.879029000	5.824970000
H	2.960019000	-3.464703000	5.898265000

S	3.590153000	-5.177306000	7.356739000
O	4.654446000	-4.068287000	7.870035000
H	5.439981000	-4.183308000	7.313948000
O	4.269967000	-5.994957000	6.388803000
O	2.992522000	-5.726587000	8.539199000

Selenocysteine (Sec)

OS 0

Diastereoisomer RR

E = -3.064330
G = -2.992225
N_{imag} = 0

C	1.312131000	-3.388869000	7.371770000
C	2.435144000	-3.764329000	6.416557000
N	1.708072000	-2.907955000	8.691531000
H	2.625123000	-3.273435000	8.947424000
H	1.754271000	-1.896806000	8.723874000
H	0.734994000	-2.595356000	6.873092000
C	0.275609000	-4.532397000	7.537100000
O	-0.103138000	-5.219831000	6.618677000
O	-0.164794000	-4.625977000	8.787045000
H	0.396006000	-3.932997000	9.247507000
H	2.028976000	-4.154713000	5.477742000
H	3.097100000	-2.915914000	6.223705000
Se	3.592337000	-5.208023000	7.139985000
H	4.551343000	-4.980246000	5.980873000
O	4.330086000	-4.527665000	8.451562000

Diastereoisomer RS

E = -3.065621
G = -2.993086
N_{imag} = 0

C	1.341395000	-3.328169000	7.402313000
C	2.631868000	-3.473108000	6.609552000
N	1.465896000	-2.993720000	8.815731000
H	2.356738000	-3.338103000	9.181465000
H	1.421502000	-1.994236000	8.972492000
H	0.760896000	-2.534389000	6.905799000
C	0.428173000	-4.574957000	7.260867000
O	0.286865000	-5.181279000	6.224606000
O	-0.200119000	-4.855503000	8.395889000
H	0.202464000	-4.172031000	9.014853000
H	2.427881000	-3.858218000	5.604480000
H	3.167970000	-2.521479000	6.532161000
Se	3.982529000	-4.708680000	7.365543000
H	2.977795000	-5.843663000	7.364797000
O	4.146302000	-4.283094000	8.955465000

OS +2

Diastereoisomer RR

E = -3.328293
G = -3.252265
N_{imag} = 0

C	1.247933000	-3.451915000	7.271115000
C	2.301433000	-4.029650000	6.336863000

Diastereoisomer RS

E = -3.330708
G = -3.254717
N_{imag} = 0

C	1.161215000	-3.414109000	7.367359000
C	2.458140000	-3.863771000	6.707755000

N	1.698211000	-2.569361000	8.340921000	N	1.263327000	-2.715656000	8.641480000
H	2.630693000	-2.828447000	8.648689000	H	2.044871000	-3.087976000	9.178987000
H	1.732289000	-1.603924000	8.036139000	H	1.414240000	-1.722305000	8.511568000
H	0.563581000	-2.871081000	6.633531000	H	0.676145000	-2.733705000	6.650190000
C	0.324792000	-4.543999000	7.871547000	C	0.132392000	-4.565565000	7.508548000
O	-0.019884000	-5.529445000	7.265065000	O	0.025826000	-5.464497000	6.708147000
O	-0.068135000	-4.242432000	9.106760000	O	-0.636415000	-4.415405000	8.582200000
H	0.446570000	-3.404393000	9.284980000	H	-0.214391000	-3.622970000	9.024120000
H	1.826111000	-4.538686000	5.493009000	H	2.268903000	-4.302731000	5.724102000
H	2.991982000	-3.271829000	5.955066000	H	3.178322000	-3.045536000	6.619615000
Se	3.486850000	-5.448869000	7.031830000	Se	3.415108000	-5.256737000	7.718181000
O	4.308488000	-4.236984000	8.164019000	O	4.932671000	-4.843711000	6.786087000
H	4.901101000	-4.769539000	8.713238000	H	5.548746000	-5.567941000	6.968244000
O	4.501210000	-5.685262000	5.788292000	O	3.575809000	-4.608968000	9.208869000

OS +4

Diastereoisomer R

E = -3.529472

G = -3.448890

N_{imag} = 0

C	1.205741000	-3.402581000	7.383195000
C	2.517257000	-3.739342000	6.682708000
N	1.263309000	-2.908011000	8.747864000
H	2.083999000	-3.260527000	9.236911000
H	1.285102000	-1.896314000	8.784731000
H	0.761403000	-2.617876000	6.751873000
C	0.167004000	-4.554425000	7.272147000
O	0.007629000	-5.193672000	6.262810000
O	-0.549837000	-4.688527000	8.382522000
H	-0.141006000	-4.005360000	8.982793000
H	2.337350000	-4.244006000	5.729452000
H	3.155226000	-2.861875000	6.541336000
Se	3.703388000	-4.959009000	7.655226000
O	5.197304000	-4.582726000	6.753014000
H	5.495487000	-5.450422000	6.434026000
O	3.393108000	-6.512483000	7.404089000
O	3.890264000	-4.392060000	9.150542000

Tellurocysteine (Tec)

OS 0

Diastereoisomer RR

E = -3.035861

G = -2.966670

N_{imag} = 0

C	1.274768000	-3.396115000	7.367023000
C	2.335371000	-3.771570000	6.340704000

Diastereoisomer RS

E = -3.036569

G = -2.966790

N_{imag} = 0

C	1.326104000	-3.308957000	7.393763000
C	2.599513000	-3.432308000	6.568042000

N	1.743687000	-2.866026000	8.641511000	N	1.473779000	-3.009480000	8.812916000
H	2.643160000	-3.284696000	8.890691000	H	2.370764000	-3.358671000	9.165865000
H	1.861640000	-1.860531000	8.608410000	H	1.429117000	-2.014219000	8.994642000
H	0.637776000	-2.630663000	6.895744000	H	0.732982000	-2.503592000	6.931105000
C	0.282754000	-4.554470000	7.646338000	C	0.407350000	-4.549997000	7.238185000
O	-0.082104000	-5.337406000	6.800302000	O	0.246121000	-5.131901000	6.190806000
O	-0.137461000	-4.552035000	8.906424000	O	-0.204414000	-4.852864000	8.376638000
H	0.425991000	-3.820381000	9.300543000	H	0.212903000	-4.183920000	9.003288000
H	1.872809000	-4.151768000	5.424551000	H	2.371733000	-3.811789000	5.566336000
H	2.979435000	-2.919519000	6.108216000	H	3.113906000	-2.469479000	6.477901000
Te	3.659400000	-5.345900000	7.042761000	Te	4.120081000	-4.756264000	7.367056000
H	4.827116000	-4.812901000	5.908783000	H	3.003745000	-6.043637000	7.407204000
O	4.248917000	-4.620529000	8.606443000	O	4.194470000	-4.233550000	9.112267000

OS +2

Diastereoisomer RR

E = -3.309378

G = -3.236009

N_{imag} = 0

C	1.212700000	-3.441059000	7.261707000
C	2.238983000	-4.011097000	6.292984000
N	1.684320000	-2.530054000	8.297706000
H	2.605320000	-2.808172000	8.624724000
H	1.756414000	-1.581120000	7.949913000
H	0.479429000	-2.882923000	6.657813000
C	0.352671000	-4.541362000	7.928531000
O	0.082531000	-5.591608000	7.394354000
O	-0.075829000	-4.181577000	9.135458000
H	0.407555000	-3.318594000	9.273952000
H	1.743516000	-4.458883000	5.425444000
H	2.943270000	-3.253193000	5.935382000
Te	3.515626000	-5.613961000	6.967387000
O	4.295371000	-4.347034000	8.306729000
H	4.915490000	-4.840875000	8.859354000
O	4.715858000	-5.624305000	5.629627000

Diastereoisomer RS

E = -3.312769

G = -3.238733

N_{imag} = 0

C	1.146761000	-3.383524000	7.354660000
C	2.403012000	-3.769021000	6.584677000
N	1.294824000	-2.677567000	8.616594000
H	2.008334000	-3.144313000	9.176002000
H	1.573832000	-1.713763000	8.473452000
H	0.539602000	-2.742452000	6.694524000
C	0.237347000	-4.601691000	7.610454000
O	0.310981000	-5.632960000	6.976199000
O	-0.646485000	-4.385064000	8.575135000
H	-0.314060000	-3.537722000	8.975943000
H	2.175545000	-4.014592000	5.542589000
H	3.163522000	-2.983892000	6.613421000
Te	3.339274000	-5.504350000	7.435815000
O	5.089248000	-4.685283000	6.963403000
H	5.779134000	-5.342867000	7.123670000
O	3.144038000	-5.112522000	9.188913000

OS +4

Diastereoisomer R

E = -3.499352

G = -3.424786

N_{imag} = 0

C	1.167278000	-3.389460000	7.363256000
C	2.371294000	-3.807759000	6.527428000
N	1.380510000	-2.841648000	8.686640000
H	2.049386000	-3.406752000	9.209791000
H	1.707134000	-1.883162000	8.661161000

H	0.674705000	-2.606992000	6.761639000
C	0.087041000	-4.499179000	7.428454000
O	-0.062679000	-5.325511000	6.563042000
O	-0.682203000	-4.389466000	8.507532000
H	-0.242021000	-3.665891000	9.023263000
H	2.066878000	-4.307308000	5.602665000
H	3.048388000	-2.976274000	6.309394000
Te	3.672991000	-5.228627000	7.485676000
O	5.296822000	-4.177727000	7.258924000
H	5.973623000	-4.793759000	6.937637000
O	3.974060000	-6.744925000	6.592654000
O	3.289519000	-5.224776000	9.236084000

Transition states

Cysteine (Cys)

OS 0

Diastereoisomer RR‡

E = -3.083027

G = -3.011276

N_{imag} = -422.916

C	1.180857000	-3.377912000	7.158706000
C	2.423058000	-3.773077000	6.631534000
N	-0.073510000	-3.841764000	6.620564000
H	-0.016668000	-4.057353000	5.631590000
H	-0.443514000	-4.654378000	7.101471000
H	1.379152000	-4.175051000	8.401880000
C	1.115437000	-2.023139000	7.807259000
O	2.067646000	-1.452136000	8.291403000
O	-0.125911000	-1.509024000	7.810412000
H	-0.645049000	-2.188062000	7.317490000
H	2.453547000	-4.459870000	5.788045000
H	3.245118000	-3.064249000	6.690502000
S	3.168388000	-5.237701000	8.217729000
O	1.860558000	-4.962185000	9.042987000
H	4.033786000	-4.312475000	8.726118000

Diastereoisomer RS‡

E = -3.081687

G = -3.010054

N_{imag} = -434.133

C	1.179569000	-3.372563000	7.159942000
C	2.437743000	-3.734094000	6.649890000
N	-0.054011000	-3.880560000	6.613713000
H	0.020686000	-4.116450000	5.630670000
H	-0.414133000	-4.688429000	7.110082000
H	1.391351000	-4.149527000	8.407284000
C	1.062943000	-2.010022000	7.792580000
O	1.987998000	-1.400034000	8.277917000
O	-0.195927000	-1.539066000	7.772747000
H	-0.685745000	-2.239559000	7.280660000
H	2.506863000	-4.391704000	5.785513000
H	3.245675000	-3.015420000	6.760576000
S	3.181154000	-5.215166000	8.230057000
O	1.886745000	-4.918137000	9.066203000
H	2.811766000	-6.301629000	7.486900000

OS +2

Diastereoisomer RR‡

E = -3.338185

G = -3.261795

N_{imag} = -237.662

C	1.167964000	-3.333329000	7.098664000
C	2.413497000	-3.702712000	6.611474000
N	-0.068286000	-3.850605000	6.578070000
H	0.030175000	-4.189345000	5.627699000
H	-0.457697000	-4.602685000	7.137404000

Diastereoisomer RS‡

E = -3.336744

G = -3.260480

N_{imag} = -246.121

C	1.175306000	-3.319521000	7.093469000
C	2.388315000	-3.703575000	6.536647000
N	-0.089296000	-3.833706000	6.637335000
H	-0.052605000	-4.143141000	5.672653000
H	-0.438598000	-4.601771000	7.200989000

H	1.437899000	-4.193635000	8.431225000
C	1.043254000	-2.007263000	7.797258000
O	1.959696000	-1.422959000	8.329010000
O	-0.213582000	-1.527004000	7.773165000
H	-0.697858000	-2.195639000	7.235191000
H	2.501395000	-4.408621000	5.788416000
H	3.241402000	-3.014362000	6.756534000
S	3.171310000	-5.320782000	8.207043000
O	2.730747000	-6.592752000	7.253874000
H	2.889195000	-7.395885000	7.771284000
O	1.910002000	-4.930752000	9.049292000

H	1.523704000	-4.140444000	8.424139000
C	1.085738000	-1.983583000	7.782577000
O	2.025373000	-1.385016000	8.252604000
O	-0.174679000	-1.512569000	7.823030000
H	-0.682746000	-2.194713000	7.324073000
H	2.412124000	-4.403991000	5.704905000
H	3.236298000	-3.029559000	6.629680000
S	3.264882000	-5.250394000	8.136187000
O	4.473365000	-4.240772000	8.584713000
H	4.910206000	-4.639788000	9.351707000
O	2.010132000	-4.901732000	9.007044000

OS +4

Diastereoisomer R‡

E = -3.577164

G = -3.500280

N_{imag} = -923.920

C	1.143373000	-3.340871000	7.121417000
C	2.387529000	-3.725583000	6.596527000
N	-0.106867000	-3.799374000	6.577427000
H	-0.060161000	-3.985916000	5.581908000
H	-0.470783000	-4.626839000	7.039097000
H	1.382950000	-4.182854000	8.321262000
C	1.078068000	-2.001788000	7.811365000
O	2.031912000	-1.440536000	8.297886000
O	-0.167810000	-1.502994000	7.842700000
H	-0.688477000	-2.168380000	7.334475000
H	2.448095000	-4.380344000	5.728187000
H	3.232423000	-3.055505000	6.748469000
S	3.057263000	-5.281909000	8.104983000
O	2.757134000	-6.754586000	7.475372000
H	3.399758000	-7.353034000	7.892331000
O	1.826816000	-5.022464000	8.982049000
O	4.299476000	-5.376264000	8.847713000

Selenocysteine (Sec)

OS 0

Diastereoisomer RR‡

E = -3.044680

G = -2.976393

N_{imag} = -685.642

C	1.175887000	-3.408032000	7.205165000
C	2.426187000	-3.800015000	6.638240000
N	-0.080328000	-3.841260000	6.637855000
H	-0.033542000	-3.978110000	5.634158000
H	-0.436558000	-4.691254000	7.060160000

Diastereoisomer RS‡

E = -3.043997

G = -2.975799

N_{imag} = -685.542

C	1.177987000	-3.391492000	7.211312000
C	2.451429000	-3.741841000	6.673710000
N	-0.049625000	-3.891552000	6.636932000
H	0.020582000	-4.061582000	5.639780000
H	-0.383561000	-4.737858000	7.084349000

H	1.316627000	-4.159483000	8.372035000
C	1.133356000	-2.026337000	7.799713000
O	2.099548000	-1.445833000	8.244070000
O	-0.104467000	-1.505340000	7.813790000
H	-0.635272000	-2.199295000	7.353399000
H	2.420219000	-4.446788000	5.761525000
H	3.229284000	-3.066034000	6.655070000
Se	3.217216000	-5.279005000	8.193951000
O	1.785046000	-5.003566000	9.078996000
H	4.109692000	-4.238026000	8.789563000

H	1.333787000	-4.106283000	8.395356000
C	1.065489000	-1.997319000	7.773793000
O	1.995546000	-1.364534000	8.220741000
O	-0.194555000	-1.532298000	7.750805000
H	-0.685217000	-2.259125000	7.296975000
H	2.498972000	-4.366843000	5.783057000
H	3.231852000	-2.986357000	6.741932000
Se	3.246872000	-5.212290000	8.244159000
O	1.822229000	-4.916506000	9.132149000
H	2.830887000	-6.406480000	7.439684000

OS +2

Diastereoisomer RR‡

E = -3.302279

G = -3.229311

N_{imag} = -446.269

C	1.171741000	-3.360977000	7.167351000
C	2.438376000	-3.731934000	6.665511000
N	-0.058951000	-3.875117000	6.618689000
H	0.019477000	-4.091081000	5.630788000
H	-0.390900000	-4.707734000	7.093541000
H	1.351664000	-4.130541000	8.424805000
C	1.055102000	-1.991898000	7.772766000
O	1.982982000	-1.368542000	8.240185000
O	-0.206366000	-1.526552000	7.760048000
H	-0.695350000	-2.240047000	7.284316000
H	2.504548000	-4.402632000	5.809323000
H	3.235521000	-2.995051000	6.745452000
Se	3.216830000	-5.267212000	8.231554000
O	2.741782000	-6.652377000	7.175410000
H	2.878932000	-7.447711000	7.709773000
O	1.813727000	-4.898926000	9.116089000

Diastereoisomer RS‡

E = -3.299445

G = -3.226799

N_{imag} = -485.027

C	1.177456000	-3.359608000	7.153975000
C	2.398889000	-3.756736000	6.565014000
N	-0.093195000	-3.839800000	6.664894000
H	-0.082415000	-4.028552000	5.668421000
H	-0.418116000	-4.673379000	7.142305000
H	1.429045000	-4.119886000	8.394815000
C	1.115220000	-1.985502000	7.767740000
O	2.071330000	-1.368549000	8.177721000
O	-0.142254000	-1.509887000	7.826276000
H	-0.663535000	-2.218942000	7.378509000
H	2.382997000	-4.388582000	5.677647000
H	3.233444000	-3.059285000	6.624531000
Se	3.256567000	-5.306278000	8.083285000
O	4.551902000	-4.178077000	8.598546000
H	4.943899000	-4.566031000	9.394388000
O	1.906285000	-4.925180000	9.043685000

OS +4

Diastereoisomer R‡

E = -3.494309

G = -3.421170

N_{imag} = -992.359

C	1.147970000	-3.359433000	7.198223000
C	2.403679000	-3.675347000	6.605474000
N	-0.087454000	-3.836963000	6.629593000
H	-0.025732000	-4.021897000	5.634906000
H	-0.435322000	-4.670216000	7.092539000
H	1.311274000	-4.049646000	8.296460000
C	1.051292000	-1.945514000	7.774499000
O	1.992996000	-1.337146000	8.217232000

O	-0.200464000	-1.481043000	7.748656000
H	-0.697999000	-2.202583000	7.291044000
H	2.461145000	-4.317190000	5.726619000
H	3.219991000	-2.965317000	6.734401000
Se	3.108164000	-5.277014000	8.147319000
O	2.735223000	-6.842516000	7.340007000
H	3.339478000	-7.470439000	7.770030000
O	1.763954000	-5.004447000	9.113379000
O	4.462503000	-5.542531000	8.982785000

Tellurocysteine (Tec)

OS 0

Diastereoisomer RR‡

E = -3.019898

G = -2.954042

N_{imag} = -795.527

C	1.165536000	-3.420388000	7.222246000
C	2.418081000	-3.803902000	6.614653000
N	-0.091615000	-3.831993000	6.639949000
H	-0.043328000	-3.953168000	5.634199000
H	-0.459273000	-4.682964000	7.049950000
H	1.270568000	-4.150596000	8.362320000
C	1.138396000	-2.027736000	7.796425000
O	2.112123000	-1.448082000	8.224776000
O	-0.095755000	-1.497985000	7.815047000
H	-0.633829000	-2.191577000	7.362613000
H	2.379038000	-4.400321000	5.702913000
H	3.204990000	-3.051035000	6.612935000
Te	3.281872000	-5.384524000	8.194155000
O	1.698232000	-5.030347000	9.117856000
H	4.277859000	-4.213759000	8.887651000

Diastereoisomer RS‡

E = -3.020093

G = -2.954400

N_{imag} = -785.411

C	1.170421000	-3.394277000	7.231603000
C	2.450923000	-3.733171000	6.658944000
N	-0.055276000	-3.898076000	6.656076000
H	0.013496000	-4.066829000	5.658511000
H	-0.385521000	-4.745121000	7.104543000
H	1.298075000	-4.066001000	8.402443000
C	1.062722000	-1.983024000	7.748996000
O	1.999124000	-1.338567000	8.167418000
O	-0.196928000	-1.517422000	7.729945000
H	-0.692511000	-2.254833000	7.299300000
H	2.465855000	-4.356278000	5.764761000
H	3.197407000	-2.940333000	6.655013000
Te	3.364536000	-5.213879000	8.309854000
O	1.763763000	-4.880495000	9.208744000
H	2.906589000	-6.584053000	7.428584000

OS +2

Diastereoisomer RR‡

E = -3.289338

G = -3.218804

N_{imag} = -659.574

C	1.164129000	-3.366615000	7.200951000
C	2.448848000	-3.724604000	6.674750000
N	-0.057264000	-3.876157000	6.628265000
H	0.026467000	-4.067071000	5.635484000
H	-0.390888000	-4.719143000	7.082747000
H	1.290975000	-4.095827000	8.434507000
C	1.054858000	-1.982960000	7.763464000
O	1.989984000	-1.352990000	8.211283000
O	-0.204112000	-1.509726000	7.750669000

Diastereoisomer RS‡

E = -3.285616

G = -3.215442

N_{imag} = -693.493

C	1.163226000	-3.384058000	7.179939000
C	2.386892000	-3.773958000	6.545290000
N	-0.109469000	-3.839244000	6.672641000
H	-0.102760000	-3.997019000	5.670547000
H	-0.444882000	-4.681013000	7.127438000
H	1.370095000	-4.125419000	8.386990000
C	1.121429000	-1.999678000	7.770789000
O	2.088414000	-1.385469000	8.160011000
O	-0.131175000	-1.511716000	7.842742000

H	-0.699694000	-2.228737000	7.291703000	H	-0.664215000	-2.218541000	7.406255000
H	2.486426000	-4.379992000	5.802718000	H	2.333103000	-4.328688000	5.608215000
H	3.206538000	-2.942027000	6.677285000	H	3.203609000	-3.051490000	6.587818000
Te	3.322239000	-5.248039000	8.298281000	Te	3.304099000	-5.417559000	8.046732000
O	2.803281000	-6.755093000	7.144556000	O	4.677045000	-4.148721000	8.620715000
H	2.889760000	-7.565961000	7.663750000	H	5.048801000	-4.466517000	9.454883000
O	1.727567000	-4.873390000	9.185187000	O	1.823304000	-4.955184000	9.080747000

OS +4

Diastereoisomer R‡

E = -3.474151

G = -3.402874

N_{imag} = -667.160

C	1.135624000	-3.350053000	7.225557000
C	2.393595000	-3.642969000	6.585812000
N	-0.093832000	-3.858866000	6.670838000
H	-0.037319000	-4.063711000	5.679926000
H	-0.427867000	-4.683733000	7.158169000
H	1.270046000	-3.954741000	8.314062000
C	1.026176000	-1.897441000	7.712594000
O	1.967122000	-1.257293000	8.108890000
O	-0.229254000	-1.450660000	7.673164000
H	-0.722542000	-2.203842000	7.263717000
H	2.422128000	-4.294801000	5.711462000
H	3.171866000	-2.881669000	6.629123000
Te	3.229249000	-5.261074000	8.216152000
O	2.778821000	-6.903005000	7.252253000
H	3.294003000	-7.610320000	7.671382000
O	1.720803000	-4.955045000	9.229544000
O	4.652079000	-5.730016000	9.200523000

Products

Dehydroalanine (DHA)

E = -2.469125

G = -2.415400

N_{imag} = 0

C	-0.847673000	-0.631163000	-0.440894000
C	0.246498000	-0.800231000	-1.192790000
N	-0.922671000	0.133358000	0.737184000
H	-0.041694000	0.572869000	0.974898000
H	-1.647969000	0.842859000	0.699336000
C	-2.085177000	-1.443445000	-0.737164000
O	-2.367223000	-1.894718000	-1.817458000
O	-2.873503000	-1.639563000	0.344906000
H	-2.399535000	-1.240851000	1.097183000

H	0.225320000	-1.512369000	-2.010460000
H	1.169830000	-0.260303000	-0.995141000

Table S15: Cartesian coordinates (Å), energies (a.u.) and imaginary frequencies (cm^{-1}) of the optimized structures for the phenyl alkyl model. Level of theory: ZORA-OPBE/TZ2P.

S

E= -4.36879005
Nimag=0

S	0.702976000	-0.021646000	-0.429614000
C	0.215009000	-0.092471000	-2.192161000
H	-0.676853000	0.536823000	-2.291863000
H	1.035281000	0.369686000	-2.753595000
C	-0.041767000	-1.521809000	-2.623307000
H	-0.837405000	-1.975438000	-2.025132000
H	0.856544000	-2.141751000	-2.528542000
H	-0.354649000	-1.542336000	-3.673239000
O	-0.486184000	-0.409853000	0.375305000
C	0.881344000	1.772559000	-0.354549000
C	-0.133651000	2.525807000	0.224133000
C	2.059304000	2.375632000	-0.790486000
C	0.019469000	3.904259000	0.333840000
C	2.201256000	3.754333000	-0.681212000
C	1.181069000	4.519947000	-0.122144000
H	-1.024247000	2.020293000	0.591530000
H	2.868198000	1.775105000	-1.205138000
H	-0.773241000	4.500047000	0.783832000
H	3.118157000	4.231276000	-1.023606000
H	1.298986000	5.598150000	-0.030058000

Se

E= -4.31404411
Nimag=0

Se	1.013714000	-0.266533000	-0.413122000
C	0.276357000	0.007447000	-2.238315000
H	-0.675183000	0.522522000	-2.075912000
H	0.971809000	0.685324000	-2.746107000
C	0.111556000	-1.315668000	-2.950860000
H	-0.539157000	-1.989183000	-2.384473000
H	1.072084000	-1.818511000	-3.110961000
H	-0.346147000	-1.157513000	-3.934841000
O	-0.212064000	-0.990313000	0.411515000
C	0.907605000	1.626432000	0.084546000
C	-0.155116000	2.027090000	0.881717000
C	1.908219000	2.517390000	-0.289492000
C	-0.231725000	3.355867000	1.289384000
C	1.822455000	3.844535000	0.121636000
C	0.752426000	4.263415000	0.907944000
H	-0.901223000	1.291088000	1.178531000
H	2.755812000	2.190010000	-0.891395000
H	-1.062993000	3.682103000	1.912805000
H	2.598693000	4.551299000	-0.167586000
H	0.691389000	5.300816000	1.232246000

Te

E= -4.28528366
Nimag=0

Te	1.027580000	-0.121299000	-0.515212000
C	0.060500000	-0.135609000	-2.460770000
H	-0.794210000	0.535477000	-2.329259000
H	0.766882000	0.324793000	-3.161329000
C	-0.356124000	-1.531875000	-2.872166000
H	-1.022759000	-1.983107000	-2.129958000
H	0.503927000	-2.198023000	-3.007251000
H	-0.897215000	-1.501377000	-3.826258000
O	-0.365030000	-0.504620000	0.583854000
C	1.079404000	2.021213000	-0.422422000
C	0.077594000	2.650693000	0.307806000
C	2.094648000	2.759310000	-1.025701000
C	0.081825000	4.038647000	0.416951000
C	2.091696000	4.147143000	-0.913078000
C	1.085118000	4.785499000	-0.193922000
H	-0.692178000	2.047196000	0.788698000
H	2.893820000	2.268138000	-1.581124000
H	-0.701415000	4.538289000	0.985469000
H	2.881757000	4.730155000	-1.384101000
H	1.087599000	5.870500000	-0.103870000

TS-S

E= -4.32291147
Nimag=-715.44

S	0.325444000	-0.602867000	-0.773759000
C	0.688516000	-0.130246000	-3.129845000
H	-0.240624000	0.422405000	-3.244300000
H	1.571117000	0.480841000	-2.950480000
C	0.813954000	-1.438993000	-3.583042000
H	0.690185000	-2.032367000	-2.307211000
H	1.802284000	-1.814537000	-3.848192000
H	-0.023584000	-1.898943000	-4.106986000
C	-1.426547000	-0.382087000	-0.684743000
C	-2.323338000	-1.421888000	-0.926470000
C	-1.898467000	0.884618000	-0.327966000
C	-3.689393000	-1.186481000	-0.818612000
C	-3.265910000	1.106484000	-0.225760000
C	-4.166720000	0.073510000	-0.471347000
H	-1.946273000	-2.407842000	-1.183847000
H	-1.199630000	1.695952000	-0.127164000
H	-4.387181000	-2.001600000	-1.004686000
H	-3.629141000	2.094647000	0.051748000
H	-5.237251000	0.249579000	-0.386496000
O	0.557559000	-2.104487000	-1.135943000

TS-Se

E= -4.28306160

TS-Te

E= -4.25786474

Nimag=-820.59

Se	0.454126000	-0.537671000	-0.730026000
C	0.711261000	-0.131986000	-3.130708000
H	-0.222610000	0.413473000	-3.248843000
H	1.593512000	0.499940000	-3.041001000
C	0.831359000	-1.459725000	-3.573030000
H	0.748492000	-2.067599000	-2.356079000
H	1.812315000	-1.801051000	-3.905117000
H	-0.014701000	-1.901981000	-4.099408000
O	0.644541000	-2.180507000	-1.138219000
C	-1.455954000	-0.317291000	-0.640937000
C	-2.318763000	-1.378687000	-0.893711000
C	-1.953580000	0.938397000	-0.294066000
C	-3.692111000	-1.173488000	-0.807327000
C	-3.328536000	1.131075000	-0.210462000
C	-4.200432000	0.077160000	-0.468386000
H	-1.913418000	-2.355437000	-1.146134000
H	-1.278837000	1.768952000	-0.086869000
H	-4.370216000	-2.002728000	-1.003765000
H	-3.718454000	2.111159000	0.059652000
H	-5.275854000	0.230003000	-0.400650000

Nimag=-886.77

Te	0.574953000	-0.640180000	-0.524244000
C	0.730771000	-0.034700000	-2.997786000
H	-0.135787000	0.623815000	-3.026889000
H	1.681663000	0.495203000	-2.959630000
C	0.665028000	-1.329073000	-3.567276000
H	0.586627000	-2.078975000	-2.445225000
H	1.575227000	-1.723441000	-4.020886000
H	-0.256909000	-1.603621000	-4.080972000
O	0.535112000	-2.366622000	-1.241354000
C	-1.502546000	-0.251641000	-0.339796000
C	-2.434039000	-1.206819000	-0.738804000
C	-1.925467000	0.965422000	0.194911000
C	-3.792690000	-0.934364000	-0.610011000
C	-3.286265000	1.227789000	0.321844000
C	-4.221636000	0.279936000	-0.082052000
H	-2.096508000	-2.159970000	-1.140316000
H	-1.205498000	1.717730000	0.517458000
H	-4.521315000	-1.681185000	-0.922544000
H	-3.614693000	2.178285000	0.739612000
H	-5.285555000	0.486674000	0.018582000

S-hyd

E= -4.85128943
Nimag=0

S	0.981063000	-0.203694000	-0.471874000
C	0.224071000	-0.111582000	-2.119290000
H	-0.781859000	0.280229000	-1.953801000
H	0.825451000	0.613797000	-2.671434000
C	0.192958000	-1.455953000	-2.813715000
H	-0.396800000	-2.183348000	-2.249120000
H	1.201733000	-1.849080000	-2.967267000
H	-0.274301000	-1.335079000	-3.798225000
O	-0.670379000	-0.628849000	0.159889000
C	0.878992000	1.533416000	0.086508000
C	-0.274679000	2.001331000	0.714861000
C	1.967163000	2.388363000	-0.083240000
C	-0.335885000	3.317151000	1.161218000
C	1.895860000	3.700570000	0.373102000
C	0.747253000	4.172596000	0.996801000
H	-1.115674000	1.330185000	0.851359000
H	2.858741000	2.021571000	-0.579770000
H	-1.244835000	3.669645000	1.646452000
H	2.754159000	4.356329000	0.234532000
H	0.697186000	5.199681000	1.354632000
O	2.587342000	0.039845000	-1.292479000
H	3.159647000	-0.507881000	-0.742984000
H	-0.446256000	-1.188804000	0.912276000

Se-hyd

E= -4.82859235
Nimag=0

Se	1.008967000	-0.299847000	-0.403076000
C	0.232400000	-0.148514000	-2.186705000
H	-0.760023000	0.272042000	-2.012494000
H	0.874160000	0.571555000	-2.698267000
C	0.178912000	-1.482312000	-2.895150000
H	-0.442554000	-2.200632000	-2.352628000
H	1.178653000	-1.903476000	-3.035731000
H	-0.265262000	-1.340701000	-3.888153000
O	-0.815971000	-0.691694000	0.205104000
C	0.885247000	1.581768000	0.150286000
C	-0.256803000	2.029047000	0.805190000
C	1.957809000	2.435429000	-0.082197000
C	-0.323551000	3.354670000	1.224786000
C	1.877551000	3.758202000	0.344090000
C	0.740652000	4.220972000	0.998030000
H	-1.079993000	1.340413000	0.973998000
H	2.836706000	2.060274000	-0.599001000
H	-1.218736000	3.707647000	1.734765000
H	2.715656000	4.428949000	0.160433000
H	0.684431000	5.255884000	1.331732000
O	2.741563000	-0.018480000	-1.282107000
H	3.346929000	-0.512306000	-0.715878000
H	-0.665792000	-1.258451000	0.971405000

Te-hyd

E= -4.82526283
Nimag=0

Te	0.967903000	-0.214815000	-0.359072000
C	0.122878000	-0.353521000	-2.310012000
H	-0.809085000	0.212296000	-2.233434000
H	0.837487000	0.177192000	-2.944221000
C	-0.104329000	-1.779494000	-2.766002000
H	-0.802630000	-2.309648000	-2.110693000
H	0.831699000	-2.344832000	-2.814986000

TS S-hyd

E= -4.80401898
Nimag=-941.71

C	1.026111000	0.067408000	-2.858477000
H	0.309717000	0.887007000	-2.889245000
H	2.011735000	0.386496000	-2.518473000
C	0.901986000	-1.012498000	-3.755491000
H	0.451377000	-1.934045000	-2.830330000
H	1.821579000	-1.465727000	-4.126090000
H	0.081872000	-0.981203000	-4.472324000

H	-0.538509000	-1.776208000	-3.773748000
O	-1.022229000	-0.166788000	0.272855000
C	1.064121000	1.919313000	-0.240425000
C	-0.007425000	2.627761000	0.296038000
C	2.209540000	2.581620000	-0.673102000
C	0.070256000	4.014585000	0.391693000
C	2.275375000	3.968714000	-0.572679000
C	1.209364000	4.686456000	-0.039966000
H	-0.895843000	2.093768000	0.623661000
H	3.034496000	2.011849000	-1.093311000
H	-0.769014000	4.570650000	0.807224000
H	3.169090000	4.488738000	-0.914869000
H	1.266448000	5.770978000	0.039046000
H	-1.023682000	-0.572347000	1.147865000
O	2.792764000	-0.248359000	-1.374318000
H	3.416839000	-0.663246000	-0.767250000

TS Se-hyd

E= -4.78448763
Nimag=-918.7

Se	0.479246000	-0.549543000	-0.640645000
C	0.928599000	0.000025000	-2.902710000
H	0.136610000	0.746302000	-2.935762000
H	1.902073000	0.418833000	-2.648305000
C	0.833162000	-1.173874000	-3.672452000
H	0.487967000	-2.051278000	-2.652210000
H	1.760190000	-1.603841000	-4.052994000
H	-0.029068000	-1.275922000	-4.331399000
C	-1.447246000	-0.356086000	-0.600662000
C	-2.272952000	-1.459422000	-0.387541000
C	-1.999878000	0.915135000	-0.750489000
C	-3.652220000	-1.283772000	-0.338069000
C	-3.380345000	1.076572000	-0.697767000
C	-4.211498000	-0.019885000	-0.493309000
H	-1.844102000	-2.448661000	-0.270439000
H	-1.352999000	1.776715000	-0.888205000
H	-4.291982000	-2.148829000	-0.170163000
H	-3.805382000	2.071897000	-0.818752000
H	-5.291276000	0.111159000	-0.449861000
O	0.169949000	-2.553103000	-1.591953000
H	0.968165000	-3.012574000	-1.301725000
O	0.838240000	1.213881000	-0.101056000
H	0.819129000	1.188871000	0.864382000

C	-1.331401000	-0.358662000	-0.746837000
C	-2.158202000	-1.422224000	-0.377466000
C	-1.893449000	0.895495000	-0.998873000
C	-3.531816000	-1.231099000	-0.281867000
C	-3.268083000	1.073764000	-0.902796000
C	-4.094053000	0.012701000	-0.546636000
H	-1.726452000	-2.395831000	-0.176108000
H	-1.257180000	1.738892000	-1.248035000
H	-4.165193000	-2.067860000	0.008115000
H	-3.693492000	2.054858000	-1.107976000
H	-5.170168000	0.157144000	-0.469017000
O	0.059524000	-2.476204000	-1.812773000
H	0.842556000	-2.942753000	-1.494686000
S	0.443451000	-0.570077000	-0.824581000
O	0.859259000	0.991502000	-0.236606000
H	0.858478000	0.903988000	0.725518000

TS Te-hyd

E= -4.77396504
Nimag=-868.02

Te	0.645700000	-0.506900000	-0.371400000
C	0.910600000	0.412500000	-2.793900000
H	0.145000000	1.167500000	-2.623400000
H	1.922700000	0.769000000	-2.608500000
C	0.677100000	-0.653900000	-3.676000000
H	0.405700000	-1.687500000	-2.738800000
H	1.532000000	-1.068200000	-4.211300000
H	-0.267400000	-0.666400000	-4.220000000
O	0.170300000	-2.338900000	-1.774100000
C	-1.462800000	-0.231400000	-0.288600000
C	-2.347800000	-1.298100000	-0.447300000
C	-1.953800000	1.051700000	-0.046900000
C	-3.719200000	-1.072300000	-0.373700000
C	-3.327000000	1.264600000	0.027400000
C	-4.214000000	0.205900000	-0.136700000
H	-1.975400000	-2.300400000	-0.633200000
H	-1.264400000	1.880100000	0.094600000
H	-4.404100000	-1.909900000	-0.498000000
H	-3.702100000	2.270000000	0.214100000
H	-5.287700000	0.375500000	-0.076400000
H	0.936800000	-2.917000000	-1.669700000
O	1.004900000	1.261000000	0.572600000
H	1.016700000	1.080900000	1.520800000

Additional References

(1) Handy, N. C.; Cohen, A. J. Left-Right Correlation Energy. *Mol. Phys.* **2001**, *99* (5), 403–412.

(2) Lee, C.; Yang, W.; Parr, R. G. Development of the Colle-Salvetti Correlation-Energy Formula into a Functional of the Electron Density. *Phys. Rev. B* **1988**, *37* (2), 785–789.

(3) Johnson, B. G.; Gill, P. M. W.; Pople, J. A. The Performance of a Family of Density Functional Methods. *J. Chem. Phys.* **1993**, *98* (7), 5612–5626.

(4) Swart, M.; Ehlers, A. W.; Lammertsma *, K. Performance of the OPBE Exchange-Correlation Functional. *Mol. Phys.* **2004**, *102* (23–24), 2467–2474.

(5) Becke, A. D. Density-Functional Exchange-Energy Approximation with Correct Asymptotic Behavior. *Phys. Rev. A* **1988**, *38* (6), 3098–3100.

(6) Becke, A. D.; Johnson, E. R. A Density-Functional Model of the Dispersion Interaction. *J. Chem. Phys.* **2005**, *123* (15), 154101.

(7) Grimme, S. Density Functional Theory with London Dispersion Corrections. *Wiley Interdiscip. Rev. Comput. Mol. Sci.* **2011**, *1* (2), 211–228.

(8) Johnson, E. R.; Mackie, I. D.; DiLabio, G. A. Dispersion Interactions in Density-Functional Theory. *J. Phys. Org. Chem.* **2009**, *22* (12), 1127–1135.

(9) Becke, A. D. Density-Functional Thermochemistry. III. The Role of Exact Exchange. *J. Chem. Phys.* **1993**, *98* (7), 5648–5652.

(10) Stephens, P. J.; Devlin, F. J.; Chabalowski, C. F.; Frisch, M. J. Ab Initio Calculation of Vibrational Absorption and Circular Dichroism Spectra Using Density Functional Force Fields. *J. Phys. Chem.* **1994**, *98* (45), 11623–11627.

(11) Zhao, Y.; Truhlar, D. G. The M06 Suite of Density Functionals for Main Group Thermochemistry, Thermochemical Kinetics, Noncovalent Interactions, Excited States, and Transition Elements: Two New Functionals and Systematic Testing of Four M06-Class Functionals and 12 Other Function. *Theor. Chem. Acc.* **2008**, *120* (1–3), 215–241.

(12) Zhao, Y.; Truhlar, D. G. A New Local Density Functional for Main-Group Thermochemistry, Transition Metal Bonding, Thermochemical Kinetics, and Noncovalent Interactions. *J. Chem. Phys.* **2006**, *125* (19), 194101.

(13) Van Lenthe, E.; Baerends, E. J.; Snijders, J. G. Relativistic Total Energy Using Regular Approximations. *J. Chem. Phys.* **1994**, *101* (11), 9783–9792.

(14) Perdew, J. P.; Yue, W. Accurate and Simple Density Functional for the Electronic Exchange Energy: Generalized Gradient Approximation. *Phys. Rev. B* **1986**, *33* (12), 8800–8802.

(15) Haas, P.; Tran, F.; Blaha, P.; Schwarz, K. Construction of an Optimal GGA Functional for Molecules and Solids. *Phys. Rev. B* **2011**, *83* (20), 205117.

(16) Perdew, J. P.; Burke, K.; Ernzerhof, M. Generalized Gradient Approximation Made Simple. *Phys. Rev. Lett.* **1996**, *77* (18), 3865–3868.

(17) Adamo, C.; Barone, V. Exchange Functionals with Improved Long-Range Behavior and Adiabatic Connection Methods without Adjustable Parameters: The MPW and MPW1PW Models. *J. Chem. Phys.* **1998**, *108* (2), 664–675.

(18) Perdew, J. P.; Chevary, J. A.; Vosko, S. H.; Jackson, K. A.; Pederson, M. R.; Singh, D. J.; Fiolhais, C. Atoms, Molecules, Solids, and Surfaces: Applications of the Generalized Gradient Approximation for Exchange and Correlation. *Phys. Rev. B* **1992**, *46* (11), 6671–6687.

(19) Zhang, Y.; Yang, W. Comment on “Generalized Gradient Approximation Made Simple.” *Phys. Rev. Lett.* **1998**, *80* (4), 890–890.

(20) Hammer, B.; Hansen, L. B.; Nørskov, J. K. Improved Adsorption Energetics within Density-Functional Theory Using Revised Perdew-Burke-Ernzerhof Functionals. *Phys. Rev. B* **1999**, *59* (11), 7413–7421.

(21) Adamo, C.; Barone, V. Physically Motivated Density Functionals with Improved Performances: The Modified Perdew–Burke–Ernzerhof Model. *J. Chem. Phys.* **2002**, *116* (14), 5933–5940.

(22) Staroverov, V. N.; Scuseria, G. E.; Tao, J.; Perdew, J. P. Comparative Assessment of a New Nonempirical Density Functional: Molecules and Hydrogen-Bonded Complexes. *J. Chem. Phys.* **2003**, *119* (23), 12129–12137.

(23) Tao, J.; Perdew, J. P.; Staroverov, V. N.; Scuseria, G. E. Climbing the Density Functional Ladder: Nonempirical Meta–Generalized Gradient Approximation Designed for Molecules and Solids. *Phys. Rev. Lett.* **2003**, *91* (14), 146401.

(24) Sun, J.; Ruzsinszky, A.; Perdew, J. P. Strongly Constrained and Appropriately Normed Semilocal Density Functional. *Phys. Rev. Lett.* **2015**, *115* (3), 036402.

(25) Adamo, C.; Barone, V. Toward Reliable Density Functional Methods without Adjustable Parameters: The PBE0 Model. *J. Chem. Phys.* **1999**, *110* (13), 6158–6170.

(26) Hamlin, T. A.; Swart, M.; Bickelhaupt, F. M. Nucleophilic Substitution (S_N2): Dependence on Nucleophile, Leaving Group, Central Atom, Substituents, and Solvent. *ChemPhysChem* **2018**, *19* (11), 1315–1330.

(27) Bento, A. P.; Solà, M.; Bickelhaupt, F. M. Ab Initio and DFT Benchmark Study for Nucleophilic Substitution at Carbon (S_N2@C) and Silicon (S_N2@Si). *J. Comput. Chem.* **2005**, *26* (14), 1497–1504.

(28) Bortoli, M.; Bruschi, M.; Swart, M.; Orian, L. Sequential Oxidations of Phenylchalcogenides by H_2O_2: Insights into the Redox Behavior of Selenium via DFT Analysis. *New J. Chem.* **2020**, *44* (17), 6724–6731.

(29) Bortoli, M.; Zaccaria, F.; Tiezza, M. D.; Bruschi, M.; Guerra, C. F.; Matthias Bickelhaupt, F.; Orian, L. Oxidation of Organic Diselenides and Ditellurides by H_2O_2 for Bioinspired Catalyst Design. *Phys. Chem. Chem. Phys.* **2018**, *20* (32), 20874–20885.

(30) Wolters, L. P.; Ren, Y.; Bickelhaupt, F. M. Understanding E2 versus S*N*2 Competition under Acidic and Basic Conditions. *ChemistryOpen* **2014**, *3* (1), 29–36..

(31) Vermeeren, P.; Hansen, T.; Jansen, P.; Swart, M.; Hamlin, T. A.; Bickelhaupt, F. M. A Unified Framework for Understanding Nucleophilicity and Protophilicity in the S_N2/E2 Competition. *Chem. - A Eur. J.* **2020**, *26* (67), 15538–15548.

(32) Zaccaria, F.; Wolters, L. P.; Fonseca Guerra, C.; Orian, L. Insights on Selenium and Tellurium Diaryldichalcogenides: A Benchmark DFT Study. *J. Comput. Chem.* **2016**, *37*, 1672–1680.
